# Investigation of the genome, taxonomy, and distribution of pigeon gammacoronavirus: insights into its relationships with other avian gammacoronaviruses

**DOI:** 10.3389/fmicb.2025.1647728

**Published:** 2025-09-03

**Authors:** Sumei Tan, Xiu Wang, Qingye Zhuang, Huanyu Gong, Ruixu Chen, Jiming Chen, Dahai Liu, Ming Liao

**Affiliations:** ^1^School of Animal Science and Technology, Foshan University, Foshan, China; ^2^Zhaoqing Agricultural School, Zhaoqing, China; ^3^Shandong Vocational Animal Science and Veterinary College, Weifang, China; ^4^College of Animal Science and Technology, Zhongkai University of Agriculture and Engineering, Guangzhou, China

**Keywords:** pigeon, coronavirus, epidemiology, evolution, genome, detection, taxonomy

## Abstract

**Introduction:**

Various coronaviruses (CoVs) are pathogenic to humans and animals. Most pathogenic CoVs belong to the *Orthocoronavirinae* subfamily, which comprises the genera of *Alphacoronavirus*, *Betacoronavirus*, *Gammacoronavirus*, and *Deltacoronavirus*. Pigeon gammacoronavirus (PgCoV) is prevalent in pigeons but remains poorly characterized.

**Methods:**

In this study, the first complete genome sequence of PgCoV was obtained through high-throughput sequencing and systematically analyzed along with other CoV genomic sequences.

**Results:**

PgCoVs exhibited significant differences from other avian gammacoronaviruses in genomic structure, phylogenetic relationships, and N-glycosylation sites in the S protein. These differences warrant classifying PgCoVs and some Australian chicken gammacoronaviruses as two new species and justify removing one existing chicken gammacoronavirus species (*Gammacoronavirus pulli*), according to the demarcation criteria set by the International Committee on Taxonomy of Viruses. Sequence analysis also revealed that both minor mutations (e.g., nucleotide substitutions) and major mutations (e.g., frameshift mutations and genomic recombination) play key roles in the evolution of gammacoronaviruses. An epidemiological survey revealed a high prevalence of PgCoVs and other avian gammacoronaviruses in their respective poultry flocks, as well as cross-species transmission of these viruses. Furthermore, evidence supporting the PgCoV replication in intestinal and kidney tissues of pigeons was identified, indicating potential pathogenicity in the digestive and urinary systems. A specific, sensitive, and reproducible fluorescent RT-PCR assay for PgCoV detection was developed.

**Discussion:**

This study expands our understanding of the genome, taxonomy, and distribution of PgCoVs and other avian gammacoronaviruses, which is significant for risk assessment, detection, and control of these viruses.

## Introduction

Coronaviruses (CoVs) are spherical, pleomorphic, enveloped viruses with a diameter of 80–160 nm, with a linear, positive-sense, and single-stranded RNA genome. They are assigned to the order *Nidovirales* and the family *Coronaviridae*, which currently comprises 4 subfamilies, 6 genera, 54 species, and some unclassified viruses (Woo et al., 2023). CoVs can infect mammals, birds, amphibians, and fish (Woo et al., 2023). CoV infections cause diverse clinical manifestations, ranging from asymptomatic to severe fatal disease (Rafique et al., 2024; Zhang et al., 2021). Severe acute respiratory syndrome coronavirus (SARS-CoV), severe acute respiratory syndrome coronavirus 2 (SARS-CoV-2), and Middle East respiratory syndrome-related coronavirus (MERS-CoV) are highly pathogenic to humans, causing significant epidemics and the catastrophic COVID-19 pandemic (Wu et al., 2021). Furthermore, infectious bronchitis virus (IBV), porcine epidemic diarrhea virus (PEDV), and numerous other CoVs cause severe diseases in domestic animals (Rafique et al., 2024; Zhang et al., 2021). Most pathogenic CoVs belong to the *Orthocoronavirinae* subfamily, which encompasses the genera of *Alphacoronavirus*, *Betacoronavirus*, *Gammacoronavirus*, and *Deltacoronavirus*.

Pigeons (*Columbia livia*) are globally prevalent and holds significance for both environmental and social reasons. Pigeons are also an important poultry species in China and other countries (Chang et al., 2023). Pigeon gammacoronaviruses (PgCoVs) are known to be prevalent among pigeons and distinct from chicken gammacoronaviruses (CgCoVs), duck gammacoronaviruses (DgCoVs), and goose gammacoronaviruses (GgCoVs), despite belonging to the same genus (Jonassen et al., 2005; Zhigailov et al., 2022; Zhuang et al., 2020).

Currently, the International Committee on Taxonomy of Viruses (ICTV) classifies *Gammacoronavirus* into three subgenera: *Cegacovirus*, *Brangacovirus*, and *Igacovirus*. *Cegacovirus* includes the species *G. delphinapteri* circulating in marine mammals, *Brangacovirus* includes the species of *G. brantae*, corresponding to GgCoVs. *Igacovirus* includes three species, *G. galli*, *G. pulli*, and *G. anatis* (Woo et al., 2023)*. G. anatis* corresponds to DgCoVs, while *G. galli* and *G. pulli* correspond to CgCoVs (commonly referred to as IBVs). In addition to gammacoronaviruses, diverse deltacoronaviruses circulate in pigeons and other domestic or wild birds (Lau et al., 2018; Woo et al., 2023).

PgCoVs remain unclassified due to a lack of available genome sequences (Woo et al., 2023). Additionally, key aspects of PgCoVs, such as their genomic features, flock-level prevalence, and detection methods, remain poorly characterized. This study aimed to address these knowledge gaps.

## Materials and methods

### Sample collection and treatment

**Figure 1:**
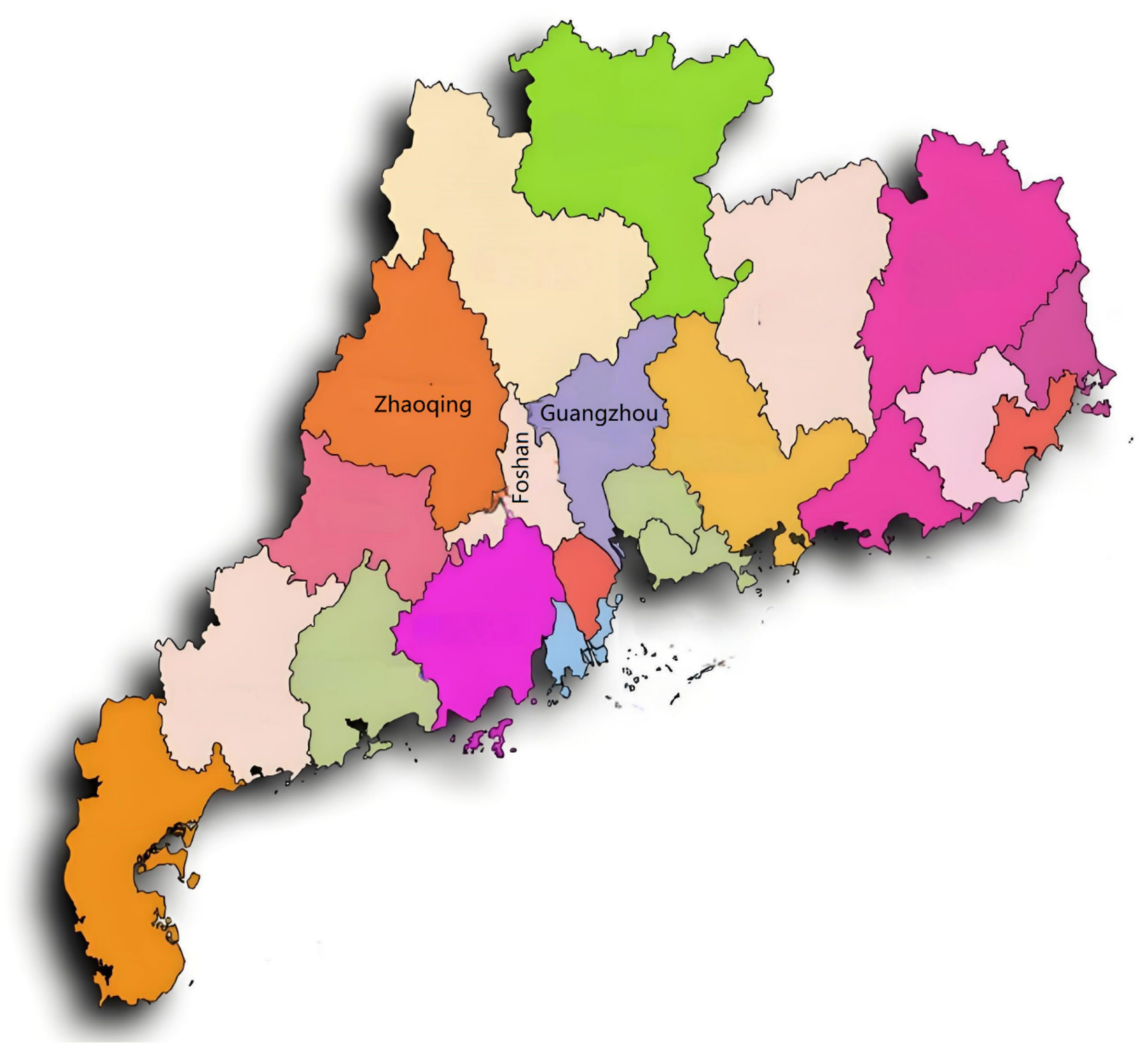
Locations of the three cities (Guangzhou, Zhaoqing, and Foshan in Guangdong Province) where the poultry samples were collected.

A total of 1,228 g of fresh pigeon feces was collected from 70 pigeon cages at 8 live bird markets in Foshan City and 50 pigeon cages at 6 live bird markets in Zhaoqing City in April 2024 for high-throughput sequencing (HTS). All fecal samples were collected from apparently healthy, white squabs of the American King Pigeon breed. Both cities are adjacent to Guangzhou City, China (Figure 1). The samples were pooled, equally divided into eight aliquots, and gently yet thoroughly mixed with 5 volumes of PBS (pH 7.2). The mixture was then centrifuged at 10,000 × g at 4 °C for 15 min; the supernatant was collected, and the pellet was removed. This process was repeated three times to completely remove the pellet. The supernatant was then gently yet thoroughly mixed with PEG 6000 (1 g/mL, final) at 4 °C for 6 h to precipitate viral particles. The mixtures were then centrifuged at 10,000 × g at 4 °C for 30 min, and the pellet was collected for RNA extraction.

Additional fecal samples were collected from 174 flocks of poultry at 12 live bird markets in the three cities of Foshan, Zhaoqing, and Guangzhou in China (Figure 1), including 44 pigeon flocks, 60 chicken flocks, 50 duck flocks, and 20 goose flocks for an epidemiological survey. Eight fresh fecal samples were collected from each flock and pooled as a flock sample. Each flock sample was mixed gently and thoroughly with 2 volumes of PBS (pH 7.2), and the mixture was then centrifuged at 10,000 g at 4 °C for 30 min, and 1 mL supernatant per sample was collected for RNA extraction.

The tissue samples from the brain, liver, spleen, lung, kidney, and intestine of 20 pigeons, which were randomly selected from four markets in Foshan City, were collected to determine the tissue distribution of PgCoVs. Each tissue sample comprised ten tissue fragments (approximately 2–3 mm in diameter) from the corresponding organ. Samples were washed using 1 mL PBS (pH 7.2) five times through centrifugation at 2,000 × g at 4 °C for 1 min, and the supernatant was discarded after each wash.

### RNA extraction

Total RNA of the samples treated above was extracted using TRIzol LS reagent (Invitrogen, United States) and subsequently purified using the RNeasy Plus Mini Kit (Qiagen, Germany), according to manufacturers’ instructions.

### HTS and data analysis

The HTS RNA library preparation was performed as described previously (Zhang et al., 2019). Briefly, RNA extracted from HTS samples was quantified using NanoDrop 2000 (Thermo Fisher, United States). The extracted RNA was then pooled into two HTS pooled samples, corresponding to samples from Foshan and Zhaoqing cities, respectively. Bacterial ribosomal RNA (rRNA) was depleted using the Ribo-Zero-Gold Kit (Illumina, United States). The remaining RNA was fragmented, reverse-transcribed, adapted, and purified using the TruSeq Total RNA Library Preparation Kit (Illumina, United States). Library quality was assessed using the Qubit High-Sensitivity RNA/DNA Assay (Thermo Fisher, China) and the Agilent 2100 Bioanalyzer (Agilent, United States). Paired-end sequencing (150-bp) was performed on the Illumina Hiseq 2500 platform.

HTS raw reads were processed using the FASTP software (v0.22.0) to remove low-quality reads, adapter sequences, barcode sequences, and sequences with poor quality at the ends of the raw reads, yielding in clean reads (Chen et al., 2018). The sequencing quality of the clean reads was analyzed using the FastQC software (v0.12.0) (de Sena Brandine and Smith, 2021). Clean reads were assembled into contigs using the MEGAHIT software (v1.2.9) (Li et al., 2015) with the default k-mer parameter, and the minimal contig length was set as 30 nt. Contigs were aligned against the NCBI non-redundant protein database (nr) using the DIAMOND software (v2.1.8) to identify homologous sequences (Buchfink et al., 2021), with the *E*-value set to 0.01. DAA files were visualized using the MEGAN software (v6.24.25) for taxonomic annotation (Huson et al., 2016). Identified viral contigs were aligned using online BLAST to verify the viral annotation information and calculate sequence similarity.

### Genomic sequence analysis

Contigs and reads were mapped to selected reference sequences using the Geneious software (Kearse et al., 2012), to identify genomic sequencing gaps, sequencing depth, and putative ORFs. The structures and functions of these ORFs were characterized by searching for conserved domains via NCBI (Wang et al., 2023), and viral genomic structures were plotted using in-house Python scripts (Supplementary Text S1).

Genomic recombination events were detected using eight methods (RDP, GENECONV, BootScan, MaxChi, Chimera, SiScan, PhyIPro, and LARD) incorporated in the RDP5 software (v5.58) (Martin et al., 2021). Events positive (*p* < 0.05) by at least four of these methods were further evaluated using the SimPlot software (v3.5.1) (Lole et al., 1999).

N-glycosylation sites on viral glycoproteins were predicted using the NetNGlyc 1.0 software (Gupta and Brunak, 2002). Differences in glycosylation sites were calculated as the count of different sites divided by the total number of sites. Transmembrane helices in the proteins were predicted using the DeepTMHMM software (Hallgren et al., 2022).

To evaluate the potential roles of frameshift mutations (FSMs) in the divergence of arteriviruses, the relevant sequences were aligned using the stringent parameters (gap opening penalty = 5.0 and gap extension penalty = 1.0) and the stringent E-INS-i mode, which is slow and suitable for less than 200 sequences with multiple conserved domains and long gaps, in MAFFT (Katoh et al., 2019). Then, insertions of deletions (indels) of nucleotides and FSMs in the aligned sequences were calculated using in-house Python scripts (Supplementary Text S1).

Information entropy (*H*) at each genomic site was calculated to characterize the variation and diversity of these sites (Santoni, 2024). Geneious was used to map HTS clean reads to the CoV/PG/FS/2024 genome and generate a BAM file, and the software SAMtools was used to read the BAM file and generate a pileup file. In-house Python scripts (Supplementary Text S1) were then used to read the pileup file and compute *H* values using the following formula:


$$H=–(P_{1}\times \log_{2}P_{1}+P_{2}\times \log_{2}P_{2}+P_{3}\times \log_{2}P_{3}+\ldots \ldots +P_{n}\times \log_{2}P_{n})$$

*P*_n_ denotes the frequency of the *n*th base call (e.g., A, T, C, G or N) at the genomic site. H values were mainly presented with means ± standard deviations (SD).

### Sample detection using RT-PCR

Fecal and tissue samples for the epidemiological survey or tissue distribution analysis were detected using a previously reported semi-nested RT-PCR assay targeting the viral RdRp gene for the detection of orthocoronaviruses (Xiu et al., 2020). Briefly, the assay included two amplification steps. The first step was performed using the One-step All-Ready RT-PCR Kit (Biotephy, China) in a 25 μL reaction system containing 2 μL RNA template, 1 μL of each primer (pan-CoV_outF and pan-CoV_R), and 21 μL reaction mix containing dNTPs, buffer, reverse transcriptase, Taq enzyme, the monoclonal antibody against Taq enzyme, and RNase inhibitor. The reaction started with the incubation at 50 °C for 30 min for reverse transcription, followed by inactivation of the reverse transcriptase at 94 °C for 2 min. Then, 40 cycles amplification at 94 °C for 15 s, 53.4 °C for 30 s, and 68 °C for 1 min. The second amplification step was conventional PCR, using 1 μL of the first RT-PCR product as template with the primers pan-CoV_inF and pan-CoV_R. To avoid possible contamination, positive and negative controls were included in each run of the assay. Positive amplicons (approximately 600 bp) were subject to Sanger sequencing.

### Establishment of a real-time RT-PCR assay

A Taqman real-time RT-PCR assay for the detection of PgCoVs was developed using the orthocoronavirus-conserved primers extended from the pan-CoV_inF and pan-CoV_R reported previously (Xiu et al., 2020) and a PgCoV-specific probe, which was conjugated with fluorescein amidite (FAM) and black hole quencher (BHQ1) at its terminals (Table 1). This assay started in turn with the incubation at 50 °C for 30 min for reserve transcription, the incubation at 94 °C for 2 min to inactivate the reverse transcriptase, and 5 cycles of amplification at 94 °C for 15 s, 53.4 °C for 30 s, and 68 °C for 1 min. This was followed by 35 cycles of amplification at 94 °C for 15 s, 53.4 °C for 30 s, and 68 °C for 1 min. The specificity, sensitivity, and repeatability of the assay were evaluated using samples from the epidemiological survey.

**Table 1:** Primers and probe for the TaqMan real-time RT-PCR detection of PgCoVs.

Name	Sequences
CoVFF	TGGGTTGGGAYTAYCCHAARTGTGA
CoVRR	GTGTGCTGIGARCARAAYTCATGIGG
PG-Probe	FAM-AAGCCTGGTGGGACTAGTAGTGGTGATGC-BHQ1

### Phylogenetic and taxonomic analysis

Amino acid sequences of five conserved domains (3CLpro, NiRAN, RdRp, ZBD, and HEL1) from CoV genomes were aligned online using the E-INS-i mode in MAFFT (Katoh et al., 2019). The best phylogenetic tree model was then analyzed using the ModelFinder tool in the PhyloSuite package (Kalyaanamoorthy et al., 2017; Zhang et al., 2020). The genetic lineage relationships were subsequently analyzed using the IQ-TREE program (Minh et al., 2020), based on the best phylogenetic tree model and the maximum likelihood method. Bootstrap support values were calculated with 1,000 replicates. At least one sequence per each classified species was selected for this analysis.

Taxonomic analysis of CoVs was performed according to the demarcation criteria for *Coronaviridae* established by ICTV (Woo et al., 2023) and other features of CoVs.

### Virus designations

Viruses were designated with their species names and the relevant GenBank accession numbers with or without additional information. Unclassified viruses were designated with their GenBank accession numbers and strain names.

## Results

### HTS and sequence mapping

The HTS generated 160,480,256 raw reads and 160,366,600 clean reads with high sequencing quality (Q20 ≥ 98.1% and Q30 ≥ 94.3%). From the clean reads, 26,662 contigs were assembled. Mapping of these contigs using DIAMOND BLASTx (*E*-value was set as 0.01) against the NCBI nr database revealed that 308 contigs were assigned to *Coronaviridae*. All 308 *Coronaviridae* contigs were confirmed as gammacoronavirus sequences through online BLAST, with no deltacoronavirus sequences identified. This suggested that gammacoronaviruses were relatively more abundant than deltacoronaviruses in the collected pigeon fecal samples.

The complete genomic sequence of a pigeon gammacoronavirus, which was designated as CoV/PG/FS/2024, was assembled. Of the 160,366,600 clean reads, 2,310,663 (1.44%) mapped to the genomic sequence using the software Geneious. Sequencing depth across the genome ranged from 2 to 46,327 (mean = 11,557). This sequence (GenBank accession number PQ037257) was deposited in GenBank at least 6 months earlier than the second complete genomic sequence of PgCoVs (Łukaszuk et al., 2025).

**Figure 2:**
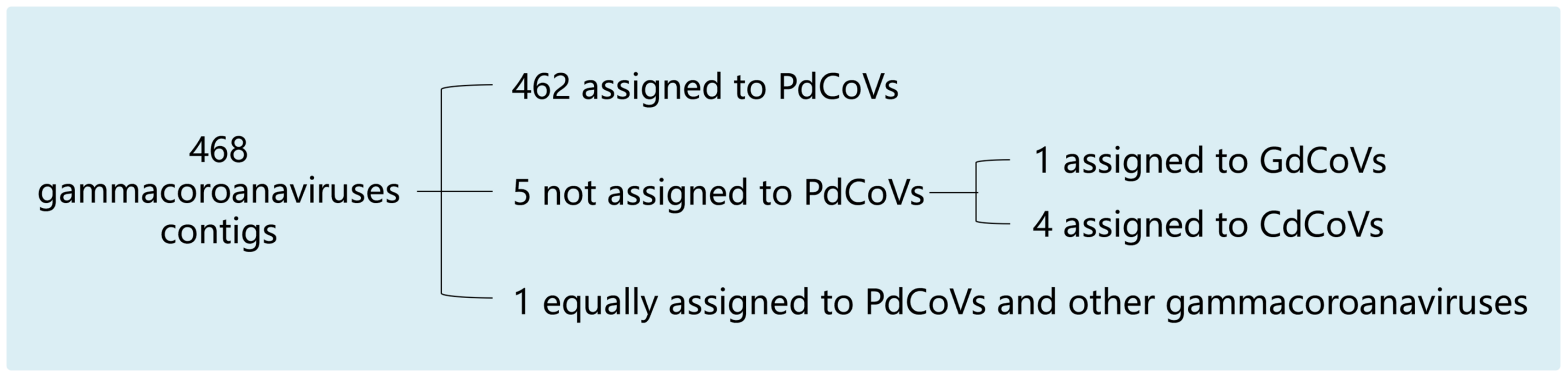
Assignment of 468 contigs to PgCoVs and other gammacoronaviruses based on sequence similarity.

Of the 26,662 contigs, 468 contigs mapped to either the CoV/PG/FS/2024genomic sequence or representative strains of the six gammacoronavirus species via the standalone BLAST (*E*-value threshold = 0.001). As shown in Figure 2, among the 468 contigs, one exhibited equal homology to PgCoVs and other gammacoronaviruses, while five contigs showed lower homology to PgCoVs than to other CoVs. In contrast, 462 contigs demonstrated higher homology to PgCoVs than to other CoVs. These data suggested that PgCoVs were more prevalent than that other avian gammacoronaviruses in the pigeon fecal samples collected for the HTS (*p* < 0.01, by the *Chi-square* test) and confirmed the presence of other avian gammacoronaviruses in these samples.

### Genomic structure of CoV/PG/FS/2024

**Figure 3:**
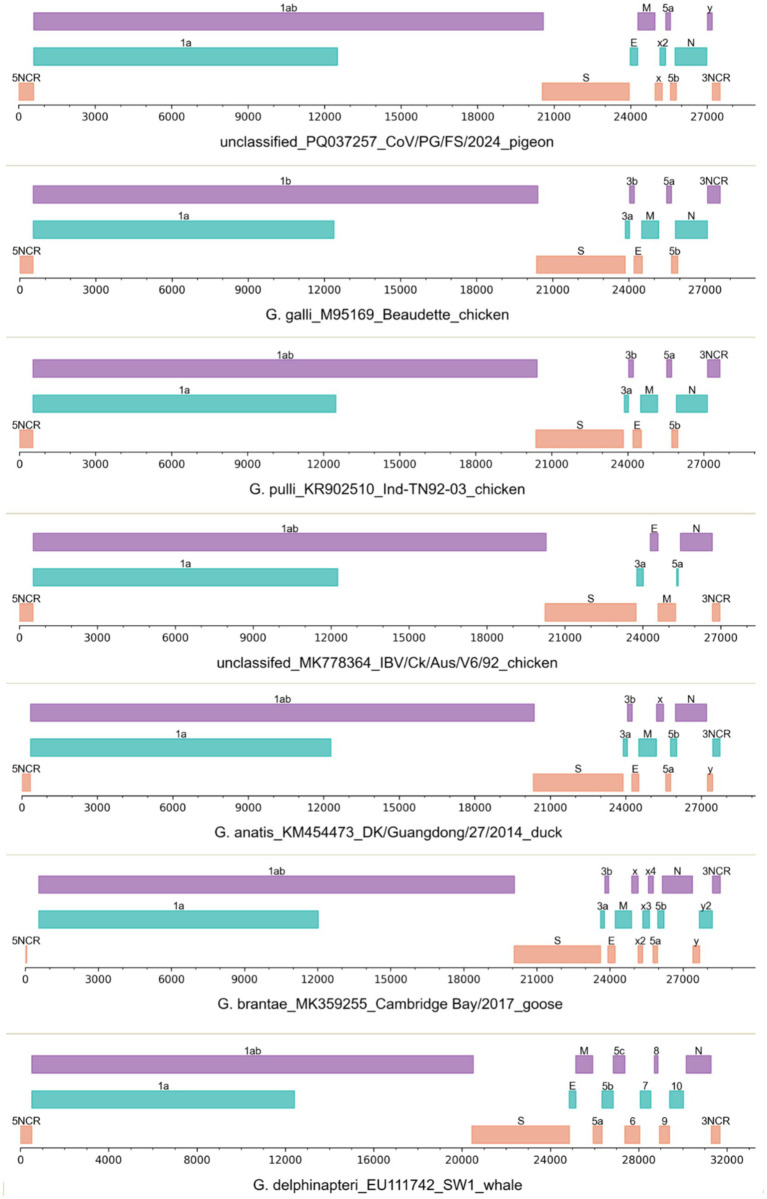
The genomic structures of seven gammacoronaviruses. The viruses were labeled with their species names, GenBank accession numbers, strain names, and host names.

The complete genomic sequence of CoV/PG/FS/2024 is 27,538 nt in length. Three-quarters of the genome at the 5′-proximal is occupied by two large ORFs, ORF1a and ORF1ab, which encode the polyproteins ppla and pplab (Figure 3). The latter is a C-terminally extended version of the former, generated via a −1 ribosomal frameshift at the last nucleotide of the TTTAAAC motif, located 30-nucleotide upstream of the ORF1a termination site (Woo et al., 2023). The polyproteins ppla and pplab are cleaved into 15 mature products, commonly termed nonstructural proteins (NSPs). Most NSPs are unique enzymes involved in one or more essential steps in viral replication (Woo et al., 2023). They constitute the replication–transcription complex that mediates RNA synthesis, ribosomal frameshifting, 3′-terminal capping, and 3′-terminal polyadenylation. Among the NSPs, NSP12 is an RNA-dependent RNA polymerase (RdRp) containing the NiRAN domain, which functions as an NMPylase participating in RNA capping. NSP14 is a 3′ → 5′-exoribonuclease, which is required for RdRp fidelity (Woo et al., 2023). Certain NSPs, such as NSP3, are involved in immune escape. Like other gammacoronaviruses, CoV/PG/FS/2024 does not encode NSP1 (Woo et al., 2023).

**Figure 4:**
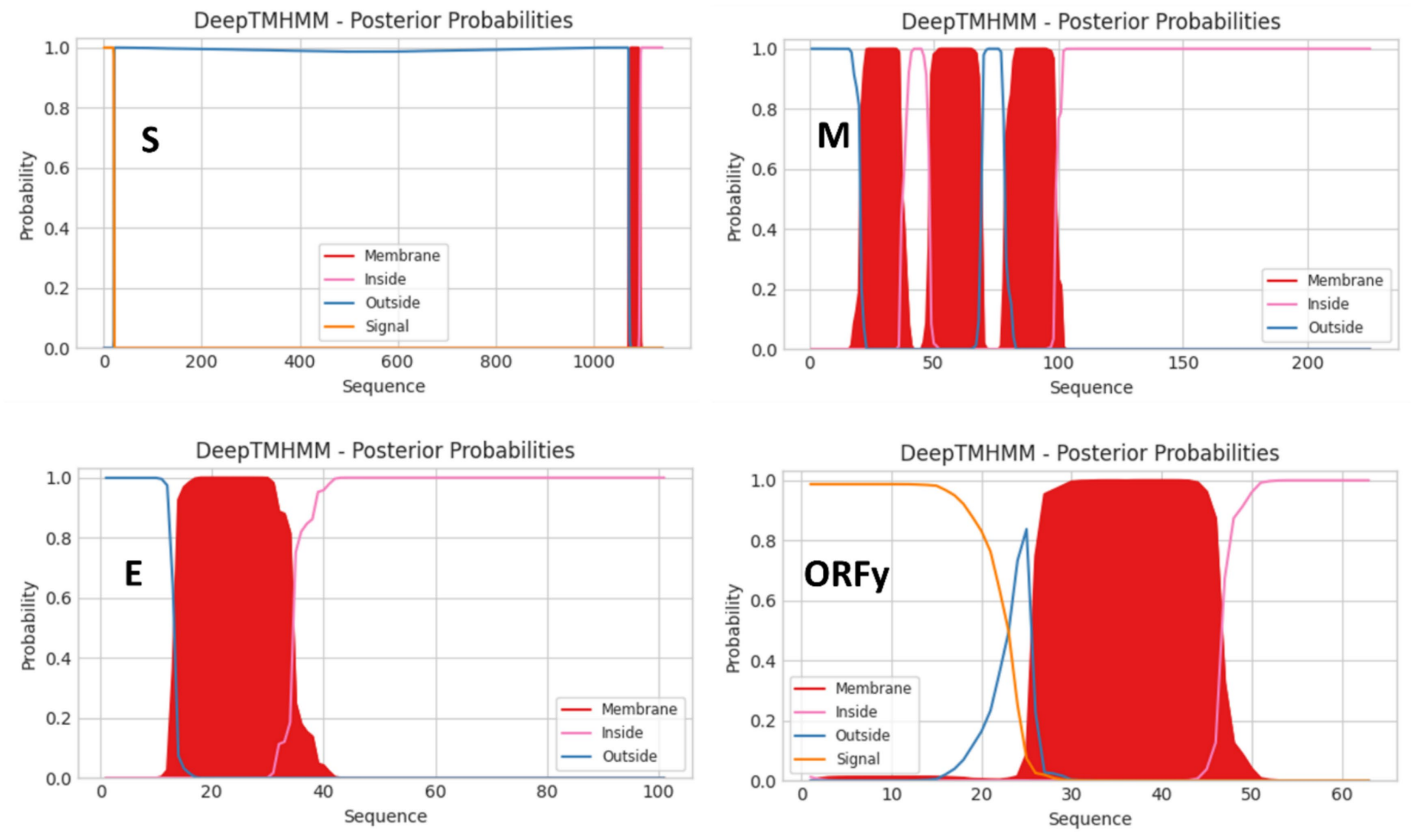
Topologies of the S, M, E, and ORFy proteins of the virus CoV/PG/FS/2024 predicted by DeepTMHMM platform.

The 3′-proximal quarter of the CoV/PG/FS/2024 genome putatively encodes the viral structural proteins S, E, M, and N (Figure 3) (Woo et al., 2023). The viral S protein is a large homo-trimeric type I membrane glycoprotein (Figure 4), which mediates receptor-binding and membrane fusion. The viral M protein is an integral type III membrane protein (Figure 4), which shows a predicted triple-spanning NexoCendo topology. It associates with the inner leaflet of the membrane to form a matrix-like lattice, which increases the thickness of the viral envelope. The viral E protein is a small pentameric integral membrane protein with ion channel activities (Figure 4), plays a role in virion assembly, and could be a virulence factor. Besides its obvious function in binding the genomic RNA, the viral N protein is involved in RNA synthesis and translation, displays RNA chaperone activity, and acts as a type I interferon antagonist.

CoV/PG/FS/2024 differs from certain avian gammacoronaviruses in lacking the accessory proteins 3a and 3b, which are encoded by some members of *Gammacoronavirus* (Figure 3). Furthermore, CoV/PG/FS/2024 putatively encodes an additional protein by ORFx2, a feature not found in other gammacoronaviruses, except for *G. brantae*_MK359255, which encoded much more accessory proteins than other gammacoronaviruses (Figure 3). Analysis of these accessory proteins using the online DeepTMHMM platform suggested that ORF5a, ORF5b, ORFx, and ORFx2 of CoV/PG/FS/2024 do not encode membrane proteins, whereas ORFy encodes a type I membrane protein (Figure 4). This is consistent with a previous study (Jonassen et al., 2005).

The genomic structure of certain IBVs (namely CgCoVs) in Australia, such as MK778364_Ck/Aus/V6/92, also differs from that of other avian gammacoronaviruses in lacking ORF3b, ORF5b, ORFx, and ORFy (Figure 3). In contrast, two species of gammacoronaviruses, *G. galli* and *G. pulli*, exhibited no differences in genomic structure (Figure 3).

Nucleotides 27,373–27,413 within the 3′-noncoding region (NCR) of CoV/PG/FS/2024 contain the s2m motif, which is conserved among CoVs and some astroviruses (Robertson et al., 2005). In CoV/PG/FS/2024, the s2M motif has the sequence aaugccgaggccacgcggaguacgaucgaggguacagcauu, and its function remains unknown.

### Phylogenetic analysis of gammacoronaviruses

**Figure 5:**
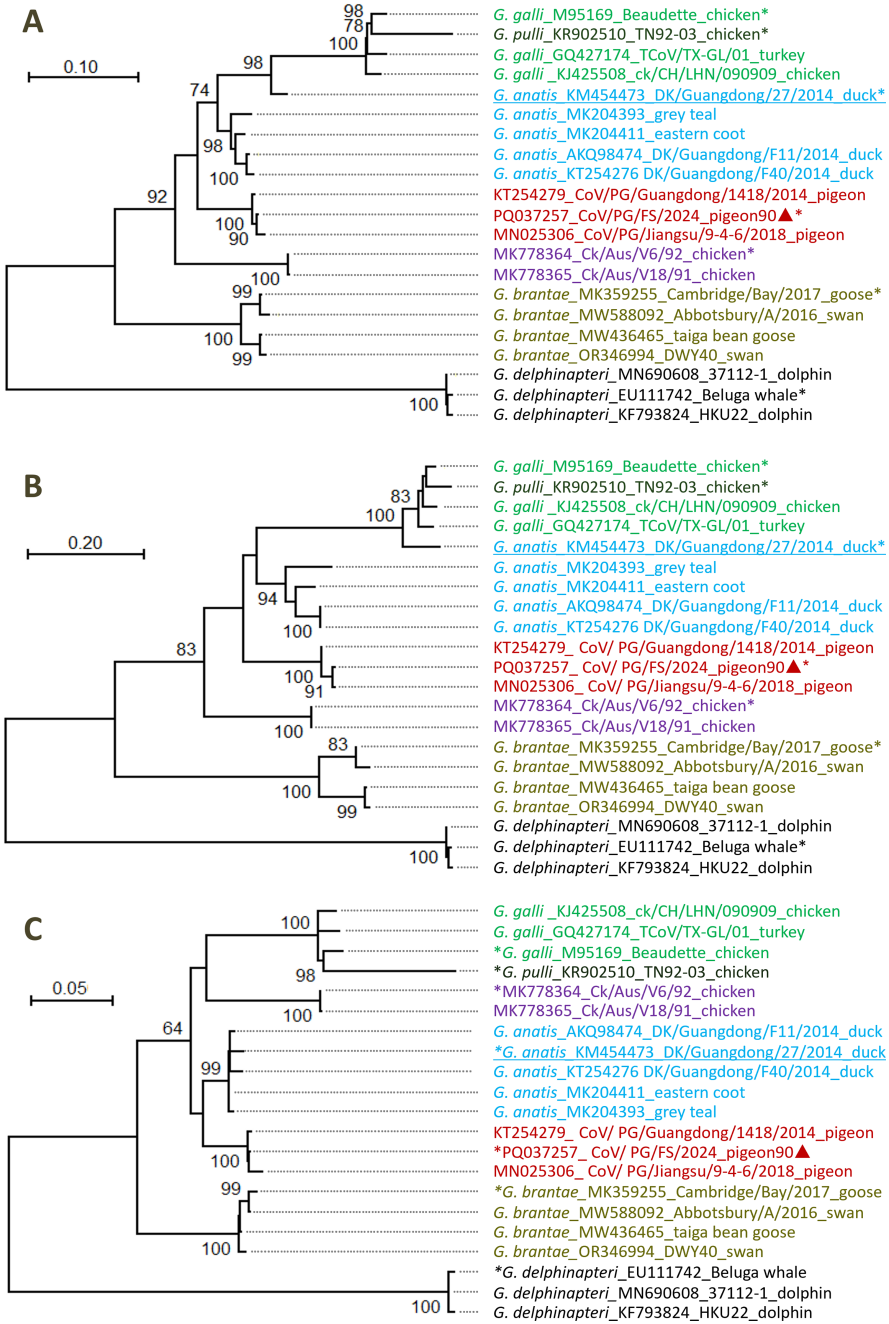
Phylogenetic relationships among certain gammacoronviruses. **(A)** According to the amino acid sequences of the five domains of 3CLpro, NiRAN, RdRp, ZBD, and HEL1. **(B)** According to the amino acid sequences of 3CLpro domain. **(C)** According to the amino acid sequences of the domains of NiRAN, RdRp, ZBD, and HEL1. Different virus species were marked with different colors. The sequences marked with asterisks represented the relevant classified or unclassified species. The sequences marked with triangles were reported by this study. The sequences with genomic recombination were underlined.

The phylogenetic relationships among four PgCoVs, namely CoV/PG/FS/2024 and three PgCoVs reported previously (Zhuang et al., 2020), and the representative strains of gammacoronaviruses were calculated based on their combined amino acid sequences of the five domains of 3CLpro, NiRAN, RdRp, ZBD, and HEL1. The results suggested that, based on the branch lengths that represent genetic distances between the viruses, PgCoVs and certain Australian IBVs, such as MK778364_Ck/Aus/V6/92 and MK778365_Ck/Aus/V18/91, constitute two separate and distinct phylogenies in the tree (Figure 5A), and thus could represent two novel species within *Gammacoronavirus*.

The DgCoV of *G. anatis*_KM454473 was not located in the phylogeny of DgCoVs (*G. anatis*) according to the amino acid sequences of the five domains (Figure 5A). This is because *G. anatis*_KM454473 is a recombinant, as its 3CLpro domain is from a strain of *G. galli* (Figure 5B), while its remaining four domains are from *G. anatis* (Figure 5C).

### Taxonomic analysis of gammacoronaviruses

Differences in the combined amino acid sequence of the five domains of 3CLpro, NiRAN, RdRp, ZBD, and HEL1, which were abbreviated as the 5DS-differences below, between the four PgCoVs (e.g., CoV/PG/FS/2024) and previously classified gammacoronaviruses shown in Figure 5A were ≥8.3% (Table 2). The 5DS-differences between the aforementioned two Australian IBVs and the classified CoVs were ≥12.2%. Consequently, based on the species demarcation criterion of CoVs established by ICTV (5DS-differences >7.5%), PgCoVs and those Australian IBVs represent two novel species of gammacoronaviruses.

**Table 2:** Differences in the combined amino acid sequences of five domains of seven classified or putative species of gammacoronaviruses*.

Virus species	*G. delphinapter*	*G. brantae*	*G. galli*	*G. pulli*	*G. anatis*	PgCoVs	AusIBVs
*G. delphinapter*	0.4–0.8%						
*G. brantae*	30.1–30.5%	0.8–4.5%					
*G. galli*	29.8–30.6%	19.8–20.2%	3.0–3.3%				
*G. pulli*	33.7–33.9%	24.4–24.5%	7.7–8.4%	0.00%			
*G. anatis*	29.7–30.2%	16.2–18.2%	9.7–13.6%	14.5–18.1%	0.8–7.9%		
PgCoVs	29.8–30.3%	17.2–17.9%	14.8–14.2%	19.2–19.4%	8.4–11.2%	0.8–1.5%	
AusIBVs	30.4–30.7%	18.4–18.8%	16.5–16.7%	20.8–21.0%	12.2–14.2%	12.9–13.4%	0.00%

*AusIBVs: certain Australian IBVs, such as MK778364_Ck/Aus/V6/92 and MK778365_Ck/Aus/V18/9.

5DS-differences between the two species, *G. galli* and *G. pulli*, could be as low as 7.70%, slightly exceeding the ICTV demarcation criterion (Table 2).

### Genomic variations of the quasi-species of CoV/PG/FS/2024

Viruses exist as quasi-species, such that their genomic sequences vary and diversify across sites within a host or a region (Dudouet et al., 2022; Santoni, 2024). Variation and diversity at genomic sites of CoV/PG/FS/2024 can be estimated using *H* values and the HTS data, because each genomic site was sequenced at high depth (mean = 11,557×).

**Figure 6:**
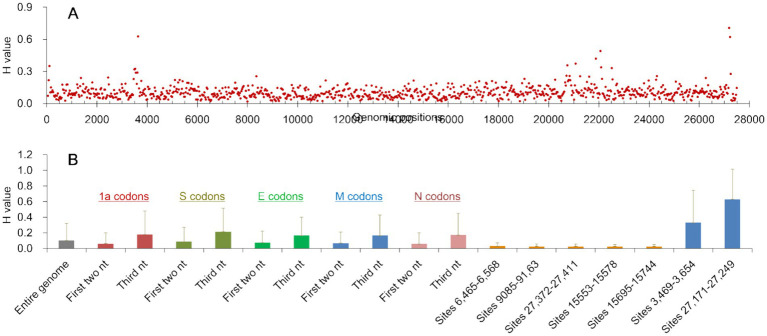
Information entropy (*H*) of the genomic sites of CoV/PG/FS/2024 calculated from high-throughput sequencing data. **(A)** The average *H* values of every 30 nucleotides of the entire genome; **(B)** The means and standard deviations of the *H* values of the entire genome and some relevant sites of the genome. nt: nucleotide(s).

*H* values across all genomic sites of CoV/PG/FS/2024 were 0.104 ± 0.217, ranging from 0.000–1.888, indicating high variability (Figure 6A). On average, each genomic site exhibited ~1.3% variation within the quasi-species.

*H* values were significantly lower on the first two nucleotides (0.061 ± 0.142) than on the third nucleotide (0.178 ± 0.297) of the codons in the viral 1a gene (*p =* 0.00, by the *t* test), as was observed in the viral S, E, M, and N genes (Figure 6B). This difference can be explained by the fact that variations at the third codon position typically results in synonymous mutations and are thus under low selection pressure.

*H* values at the first two codon positions were significantly lower in the 1a and N genes (0.061 ± 0.142) than in the S and E genes (0.086 ± 0.186) (*p =* 0.00, by the *t* test). This is because the viral S and E proteins experience weaker negative selection and stronger positive selection than the viral 1a and N genes.

The *H* value distribution exhibited three highly conserved regions in the genome: the nucleotide sites 6,465–6,568 (*H* = 0.029 ± 0.040), 9,085–9,163 (*H* = 0.027 ± 0.033), and 27,372–27,411 (*H* = 0.029 ± 0.031) (Figure 6B). These regions map to the C-terminal of the viral NSP3 gene, the C-terminal of the NSP5 gene, and the aforementioned s2m motif. Their *H* values were significantly lower than the genome-wide average (*p* = 0.00, by the *t* test). Furthermore, the *H* value distribution revealed two highly variable regions in the genome: the nucleotide sites 3,469–3,652 (*H* = 0.329 ± 0.416) and 27,171–27,229 (*H* = 0.672 ± 0.381) (Figure 6B). These sites map to the N-terminal of the NSP3 gene and the ORFy gene. Their *H* values were significantly higher than the genome-wide average (*p* = 0.00, by the *t* test). The differences above in H values indicate that selection pressures vary across the viral genome at both the sites and regional levels. For example, the viral s2m motif is subject to stronger negative selection pressure and weaker positive selection than certain other regions (Robertson et al., 2005).

### Genomic recombination of gammacoronaviruses

**Figure 7:**
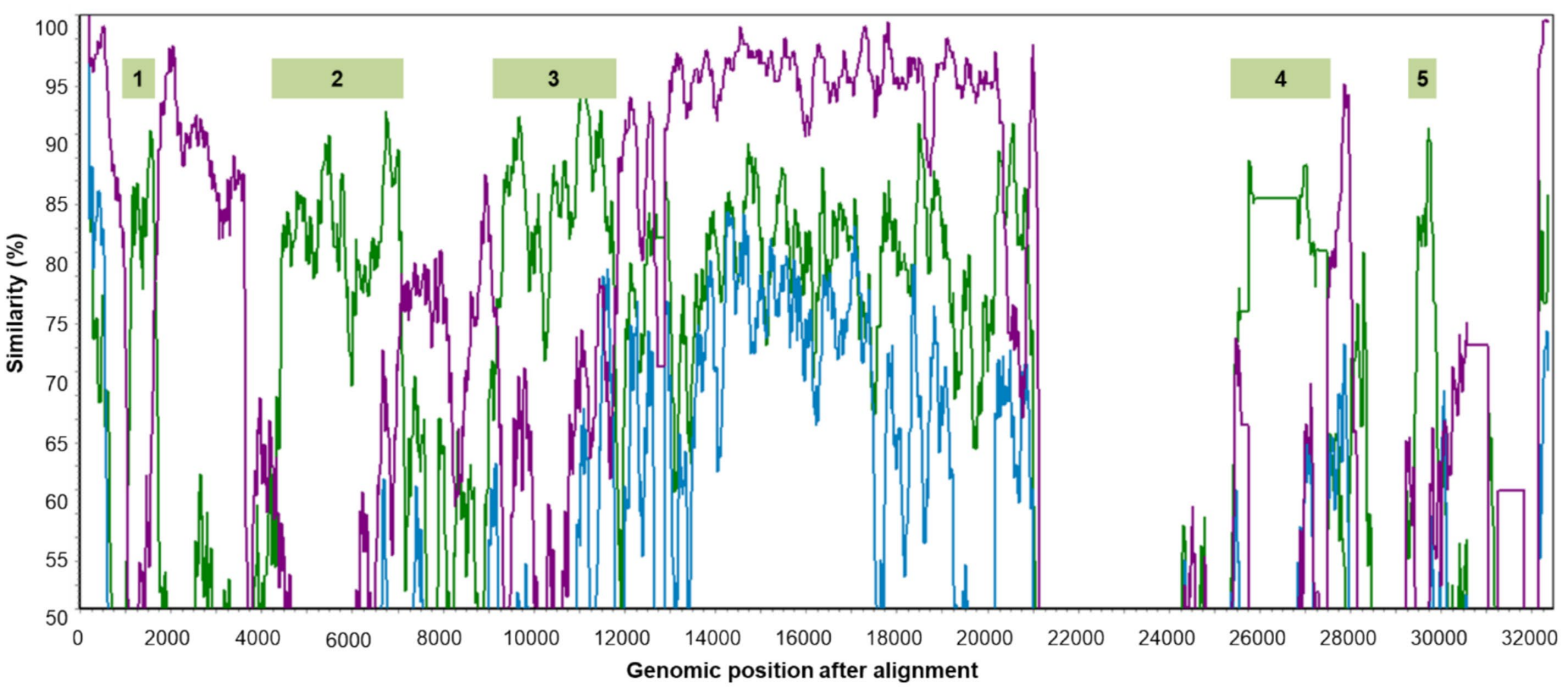
Five genomic regions of *G. anatis*_KM454473 probably from genomic recombination. The green, blue, and purple lines show the genomic similarity plots of *G. anatis*_KM454473 against *G. galli*_M95169, *anatis*_MK204393, and *G. brantae_*MK359255, respectively.

Seven genomic sequences representing the five classified species of gammacoronaviruses, CoV/PG/FS/2024, and certain Australian IBVs marked with asterisks in Figure 5 were aligned with the online MAFFT platform. Ten potential genomic recombination events were identified from these sequences using four or more methods incorporated in RDP5 (*p* < 0.05, by the relevant permutation tests). Eight of these events, which were all associated with *G. anatis*_KM454473, were supported by the software SimPlot, suggesting that *G. anatis*_KM454473 is a recombinant of a DgCoV (major parent: *G. anatis*_MK204393) and a CgCoV (minor parent: *G. galli*_M95169). The relevant similarity plots calculated by SimPlot showed that five genomic regions (sites 977–1,597, 4,026–6,705, 8,697–11,241, 23,713–25,131, and 26,422–26,018) of *G. anatis*_KM454473, totaling 26.5% of the genome, were likely derived from the CgCoV, while at least 55.5% of the genomic sites were likely derived from the DgCoV (Figure 7).

### N-linked glycosylation sites in the viral S protein

Among the seven representative CoVs mentioned above, CoV/PG/FS/2024 exhibited significant differences (≥88.9%) from other six avian gammacoronaviruses in the N-linked glycosylation sites of the viral S protein (Table 3). The difference in the N-linked glycosylation sites was 10.0% (3/30) between the *G. galli* representative strain (Beaudette) and the *G. pulli* representative strain (Ind-TN92-03), significantly lower than the difference (48.6%) between Beaudette and certain Australian IBVs, such as MK778364_Ck/Aus/V6/92.

**Table 3:** Differences in the N-linked glycosylation sites on the viral glycoprotein S of seven gammacoronaviruses.

Sequence 1	Sequence 2	Difference
*G. galli*_M95169	*G. pulli*_KR902510	3.70%
*G. galli*_M95169	MK778364_IBV/Ck/Aus/V6/92	42.86%
*G. galli*_M95169	*G. anatis*_KM454473	83.33%
*G. galli*_M95169	CoV/PG/FS/2024	88.68%
*G. galli*_M95169	*G. brantae*_MK359255	60.00%
*G. galli*_M95169	*G. delphinapteri*_EU111742	95.00%
*G. pulli*_KR902510	MK778364_IBV/Ck/Aus/V6/92	45.71%
*G. pulli*_KR902510	*G. anatis*_KM454473	82.98%
*G. pulli*_KR902510	CoV/PG/FS/2024	88.46%
*G. pulli*_KR902510	*G. brantae*_MK359255	62.50%
*G. pulli*_KR902510	*G. delphinapteri*_EU111742	94.92%
MK778364_IBV/Ck/Aus/V6/92	*G. anatis*_KM454473	90.38%
MK778364_IBV/Ck/Aus/V6/92	CoV/PG/FS/2024	90.91%
MK778364_IBV/Ck/Aus/V6/92	*G. brantae*_MK359255	57.50%
MK778364_IBV/Ck/Aus/V6/92	*G. delphinapteri*_EU111742	93.33%
*G. anatis*_KM454473	CoV/PG/FS/2024	80.39%
*G. anatis*_KM454473	*G. brantae*_MK359255	86.27%
*G. anatis*_KM454473	*G. delphinapteri*_EU111742	93.44%
CoV/PG/FS/2024	*G. brantae*_MK359255	82.69%
CoV/PG/FS/2024	*G. delphinapteri*_EU111742	93.75%
*G. brantae*_MK359255	*G. delphinapteri*_EU111742	95.16%

### FSMs in the evolution of PgCoVs

During the divergence between *G. anatis*_KM454473 and CoV/PG/FS/2024, which belong to the same subgenus *Igacovirus*, the viral 1a gene accumulated 22 insertions or deletions of nucleotides (indels), 5 of which were FSMs, with 1.0% of the nucleotide sites in this gene were involved in the FSMs (Supplementary Text S1). Meanwhile, during the divergence of the two viruses, the viral S gene accumulated 41 indels, 18 of which were FSMs, with 16.5% of the nucleotide sites in this gene were involved in the FSMs, during the divergence of the two coronaviruses.

During the divergence between a dolphin CoV, *G. delphinapteri_*EU111742 and CoV/PG/FS/2024, which belong to different subgenera, the viral 1a gene accumulated 143 indels, 58 of which were FSMs, with 15.1% of the nucleotide sites in this gene were involved in the FSMs (Supplementary Text S1). Because CoV/PG/FS/2024 was too distinct from the dolphin CoV in their S gene sequences, FSMs could not be identified with confidence and were not analyzed thereby.

### The epidemiological survey

**Figure 8:**
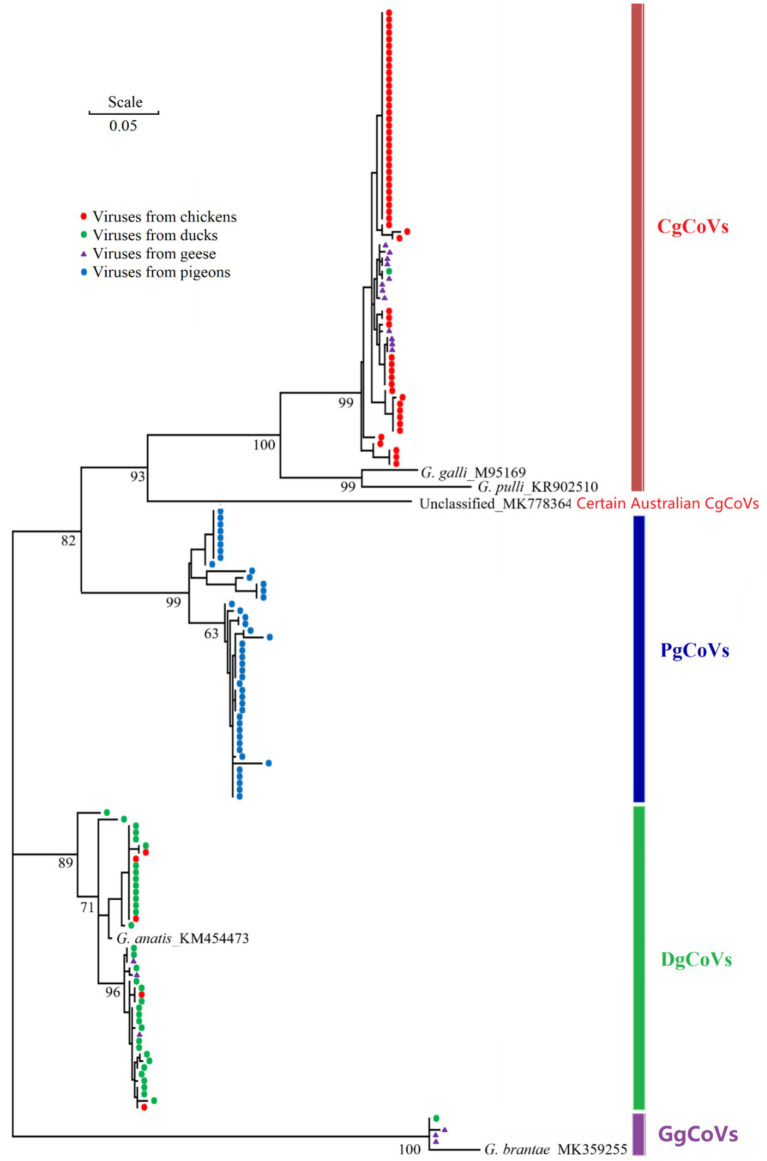
Phylogenetic relationships of 159 poultry gammacoronaviruses based on the partial sequences of the virus nsp12 (RdRp) gene. The five reference sequences were labeled with their species names and GenBank accession numbers.

Of the 174 flock fecal samples for the epidemiological survey, 159 tested positive via the nested RT-PCR assay. Sanger sequencing of amplicons of these positive samples yielded partial RdRp gene sequences of 159 orthocoronaviruses. Phylogenetic analysis classified these 159 orthocoronaviruses into 69 CgCoVs, 43 DgCoVs, 4 GgCoVs, 43 PgCoVs, and no deltacoronaviruses (Figure 8).

As shown in Table 4, from the viral perspective, the CgCoV positive rate in chicken flocks (91.67%) was significantly higher than that in goose flocks (65.00%), which was in turn significantly higher than those in duck flocks (2.00%) and in pigeon flocks (0.00%). The DgCoV positive rate in duck flocks (70.00%) was higher than that in goose flocks (15.00%), which was in turn significantly higher than those in chicken flocks (8.33%) and in pigeon flocks (0.00%). The GgCoV positive rate in goose flocks (15.00%) was significantly higher than those in duck flocks (2.00%), chicken flocks (0.00%), and pigeon flocks (0.00%). The positive rate of PgCoVs in pigeon flocks (97.73%) was significantly higher than that in other poultry flocks (0.00%).

**Table 4:** Positive rates of four coronaviruses in the flocks of four species of poultry.

Host	Positive rates and the number of positive samples versus the total number of samples tested given in parentheses			
	CgCoV	DgCoV	GgCoV	PgCoV
Chicken	91.67% (55/60)	8.33% (5/60)	0% (0/60)	0% (0/60)
Duck	2.00% (1/50)	70.00% (35/50)	2.00% (1/50)	0% (0/50)
Goose	65.00% (13/20)	15.00% (3/20)	15.00% (3/20)	0% (0/20)
Pigeon	0% (0/44)	0% (0/44)	0% (0/44)	97.73% (43/44)

From the host perspective, in chicken flocks, the CgCoVs positive rate (91.67%) was significantly higher than that of other gammacoronaviruses (≤8.33%). In duck flocks, the DgCoV positive rate (70.00%) was significantly higher than that of other gammacoronaviruses (≤2.00%). Interestingly, in goose flocks, the CgCoV positive rate (65.00%) was significantly higher than that of other gammacoronaviruses (15.00%), including DgCoVs, PgCoVs, and GgCoV.

Both the CgCoV positive rate in chicken flocks and the PgCoV positive rate in pigeon flocks exceeded 90%. They were significantly higher than the DgCoV positive rate in duck flocks (70.00%), which was in turn significantly higher than the GgCoV positive rate in goose flocks (15.00%).

The significant differences above in the positive rates in this section were determined based on the *Chi-square* test with *p* < 0.01.

### Detection of the tissue samples

RT-PCR testing of tissue samples of heart, liver, spleen, lung, kidney, brain, intestine, and serum collected from 20 randomly selected pigeons revealed positive results only in intestinal tissues (four pigeons) and kidney tissues (three of the same four pigeons) were positive for the RT-PCR assay. The positive results were confirmed using Sanger sequencing.

### Evaluation of a real-time RT-PCR assay

**Figure 9:**
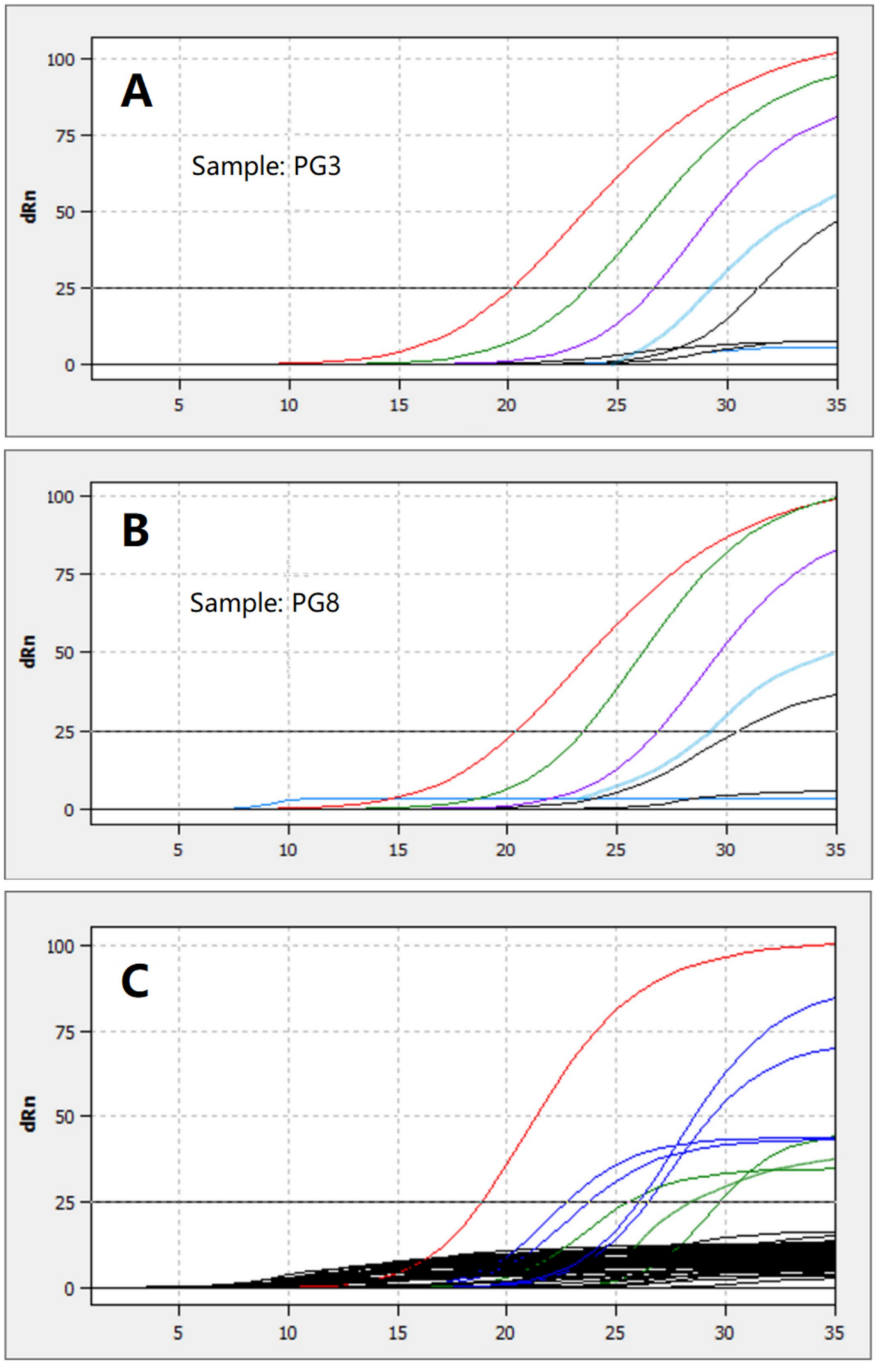
Detection of the RNA extracted from two PgCoV-positive samples and serially diluted from 1:1 to 1:100000 **(A,B)** and detection of 8 PgCoV-positive samples (the red, blue, or green lines in **C**) and 45 other samples using the TaqMan real-time RT-PCR (the black lines in **C**).

RNA extracted from four randomly selected PgCoV-positive samples from the survey described above was serially diluted 10-fold in pure water and detected using the TaqMan assay developed in this study. The assay detected the virus at 1:10000 dilution, as partially shown in Figures 9A,B.

All 8 randomly selected PgCoV-positive samples tested positive using the TaqMan real-time RT-PCR assay established in this study for PgCoV detection. In contrast, none of the 20 randomly selected samples positive for other gammacoronaviruses (8 CgCoVs, 8 DgCoVs, and 4 GgCoVs) or the 30 randomly selected samples negative for gammacoronaviruses, which were all from the survey described above, tested positive in the TaqMan real-time RT-PCR (Figure 9C).

The above detection steps were repeated three times, with consistent results obtained.

## Discussion

HTS is increasingly utilized to discover new avian viruses (Domańska-Blicharz et al., 2023; Lu et al., 2024). However, it remains challenging to interpret the epidemiological significance of virus HTS data. This study employed the distribution of HTS contigs to denote relative prevalence of deltacoronaviruses, PgCoVs, and other avian gammacoronaviruses in pigeon flocks (Figure 2). It also employed information entropy values, which were calculated from the HTS data, to characterize the nucleotide variability across sites and regions in the PgCoV genome. Thus, this study demonstrates HTS contig distribution among viruses and information entropy values can be utilized for interpreting the epidemiological significance of virus HTS data.

The CoV/PG/FS/2024 genome sequence revealed in this study is reliable, as evidenced by the high Q20 and Q30 values of the HTS clean reads and an average sequencing depth of 11,557-fold across the genome. Although PgCoVs were first identified in 2005 and found to be dominant in pigeons in the 2010s (Jonassen et al., 2005; Zhuang et al., 2020), the lack of a complete genome has hindered their recognition as a distinct species within the genus *Gammacoronavirus*.

This study provided robust evidence that PgCoVs and certain Australian IBVs, which were exclusively distributed in Australia (Rafique et al., 2024), represent two novel *Gammacoronavirus* species. Their classification is supported not only by the official ICTV demarcation criterion based on sequence differences but also by host or geographic distribution, phylogenetic relationships, and N-linked glycosylation sites of the viruses, as revealed by this study. It is further supported by genomic structure because the accessory proteins encoded by PgCoVs and certain Australian IBVs differ from those of other gammacoronaviruses, though the functions of the accessory proteins remain unclear.

This study suggested that the current classification of *G. pulli* warrants re-evaluation. Although 5DS-differences between the two species, *G. galli* and *G. pulli*, slightly exceed the ICTV’s demarcation criterion for novel CoV species (Table 2) (Domańska-Blicharz et al., 2023), this single metric is insufficient to justify its classification as a distinct species. Currently, only one sequence in GenBank is assigned to *G. pulli*, and this sequence does not represent a distinct phylogeny in the phylogenetic tree (Figure 5). Furthermore, *G. pulli* exhibits little differences from *G. galli* in overall genomic sequences, genomic structure, and the N-linked glycosylation sites of the viral S protein (Figure 3; Tables 2, 3). Moreover, relying solely on fixed genetic distance thresholds for classification is problematic, as genetic distances both within and between species tend to accumulate over time. A robust classification system should therefore integrate multiple lines of evidence, including genetic distances, phylogenetic relationships, genomic architecture, and host tropism. Together, our findings suggest that both the taxonomic classification of species within *Gammacoronavirus* and the species demarcation approach require refinement.

Genomic recombination events are frequent within a CoV species because various strains within a species can infect the same host (Kim et al., 2024; Sayama et al., 2024; Zhang et al., 2023). Recombination events are likely less frequent between CoV species, as each CoV species is typically dominant in a specific host, reducing cross-species transmission. This study showed that inter-species genomic recombination events may be more prevalent in DgCoVs or *G. anatis* than in other *Gammacoronavirus* species, consistent with a recent report (François et al., 2023). This could result from waterfowls having greater opportunities for contact with diverse gammacoronaviruses shed by other birds into water bodies. This study suggested that the virus *G. anatis*_KM454473 is a recombinant and should not be selected as the representative strain for its species.

Information entropy analysis in this study showed that most genomic sites of the PgCoV are variable and prone to nucleotide substitutions, which are minor mutations. This study also demonstrated that indels, FSMs, and genomic recombination events, which can significantly change some biomedical features of viruses, contribute to the evolution of gammacoronaviruses. Therefore, both minor and major mutations are important for gammacoronavirus evolution.

Beyond PgCoVs, other gammacoronaviruses (e.g., CgCoVs, DgCoVs, and GgCoVs) and deltacoronaviruses can also infect pigeons at relatively low prevalence (Lau et al., 2018; Zhuang et al., 2020). The HTS data and the epidemiological survey in this study further confirmed that PgCoVs are significantly more prevalent in pigeons than other gammacoronaviruses and deltacoronaviruses. The prevalence of CgCoVs, DgCoVs, GgCoVs, and PgCoVs in their correspondingly host species at the poultry flock level was significantly higher than that at the poultry individual level by multiple folds (Lau et al., 2018; Zhuang et al., 2020). While this conclusion is not surprising, the prevalence of domestic animal viruses at the host flock or herd level is important because domestic animals are usually raised, transported, and sold at the flock or herd level. The epidemiological survey also demonstrated that cross-species transmission of avian gammacoronaviruses exist, particularly in geese. Although no cross-species transmission of avian gammacoronaviruses in pigeons was found through the epidemiological survey, this could result from the fact that PgCoVs could be significantly more abundant than other gammacoronaviruses in the pigeon flock samples, and hence only PgCoVs were detected through the survey.

The evidence from this study supporting the PgCoV replication in intestinal and kidney tissues of pigeons indicated the potential pathogenicity of PgCoVs in the digestive and urinary systems of Pigeons. A recent epidemiological survey did not find that PgCoVs is more prevalent in diseased pigeons than in healthy pigeons (Łukaszuk et al., 2025). It is valuable in the future to isolate pigeon coronavirus using embryo chicken or pigeon eggs and investigate the pathogenicity of the virus through the inoculation of specific-pathogen free pigeons with the purified viruses.

Evaluation of the TaqMan real-time RT-PCR assay for PgCoV detection confirmed that the assay is specific, relatively sensitive, and reproducible. Consequently, this assay could be used for future detection and research of PgCoVs.

Together, this study employed robust methodologies, such as HTS, bioinformatics analysis, epidemiological surveys, and sample detection, to characterize the genomic sequence, epidemiological distribution, evolutionary features, taxonomy, host distribution, and tissue distribution of PgCoVs. It provided multi-faceted evidence supporting that PgCoVs and some Australian IBVs represent two novel species of gammacoronaviruses and that the classification of the species *G. pulli* warrants re-evaluation. This study expands our understanding of PgCoVs and other avian gammacoronaviruses, thereby improving approaches for their risk assessment, detection, and control.

## Data Availability

The genomic sequence reported in this study is available in GenBank (PQ037257). The other data that support the findings of this study are available from the corresponding author upon reasonable request.
