# Supplementary material for: Investigation of the genome, taxonomy, and distribution of pigeon gammacoronavirus: insights into its relationships with other avian gammacoronaviruses
Source: Front Microbiol. 2025 Sep 3;16:1647728. doi: 10.3389/fmicb.2025.1647728 (PMC12441043; doi:10.3389/fmicb.2025.1647728)
Supplement: Supplementary file 1 [file Data_Sheet_1.pdf]

Supplementary information of the article

Investigation of the genome, taxonomy, and distribution of pigeon gammacoronavirus: Insights into its relationships with other avian gammacoronaviruses

Content

1. The python scripts for calculating indels in nucleotide sequences (Page 1)
2. The python scripts for calculating the information entropy values (Page 3)
3. The python scripts for drawing virus genomic structures (Page 4)
4. Alignment of three pairs of viral gene sequences (Page 7)

1. The python scripts for calculating indels in nucleotide sequences

```
import csv
import tabulate

def read_sequences(file_path):
    sequences = {}
    with open(file_path, 'r') as file:
        current_id = ""
        current_seq = ""
        for line in file:
            line = line.strip()
            if line.startswith('>'):
                if current_id:
                    sequences[current_id] = current_seq
                current_id = line[1:]
                current_seq = ""
            else:
                current_seq += line
        if current_id:
            sequences[current_id] = current_seq
    return sequences

def count_variations(seq1, seq2):
    variations = {}
    start_index = None
    insertions = 0
```

```
    deletions = 0
    for i in range(min(len(seq1), len(seq2))):
        if seq1[i] != seq2[i]:
            if seq1[i] == '-':
                deletions += 1
            elif seq2[i] == '-':
                insertions += 1
            if start_index is None:
                start_index = i
        else:
            if start_index is not None:
                variations[(start_index, i)] = (insertions, deletions)
                start_index = None
                insertions = 0
                deletions = 0
    if start_index is not None:
        variations[(start_index, len(seq1) - 1)] = (insertions, deletions)
    return variations

def print_variations_table(variations):
    table = []
    for start, end in variations.keys():
        insertions, deletions = variations[(start, end)]
        Affected_sites = insertions - deletions
        if Affected_sites != 0:
            table.append([start, end, Affected_sites])
    print(tabulate.tabulate(table, headers=['Start', 'End', 'Affected_sites'],
tablefmt='github'))

file_path = "C:\\Users\\Acer\\Desktop\\CoV1aD.fasta"
sequences = read_sequences(file_path)

seq1 = sequences['PdCoV1a']
seq2 = sequences['EU111742']

variations = count_variations(seq1, seq2)

print_variations_table(variations)
with open('C:\\Users\\Acer\\Desktop\\1aD.csv', 'w', newline='') as csvfile:
    csvwriter = csv.writer(csvfile)
    csvwriter.writerow(['Start', 'End', 'Affected_sites'])
    for start, end, Affected_sites in variations.items():
        csvwriter.writerow([start, end, Affected_sites])
```

2. The python scripts for calculating the information entropy values of genomic sites

```
from Bio import SeqIO
from Bio.Align import PairwiseAligner
import concurrent.futures

input_fasta_path = '/home/dell/tan/RQ16/CoV6.fasta'
output_fasta_path = '/home/dell/tan/RQ16/CoV6output.fasta'
sequences = list(SeqIO.parse(input_fasta_path, 'fasta'))
def calculate_similarity(seq1, seq2):
    aligner = PairwiseAligner()
    aligner.mode = 'local'
    alignment = aligner.align(seq1, seq2)
    matches = sum(1 for a, b in alignment if a == b)
    similarity = matches / max(len(seq1), len(seq2))
    return similarity

to_remove = {}
with concurrent.futures.ThreadPoolExecutor() as executor:
    def compare_sequences(i, j):
        if sequences[i].id not in to_remove and sequences[j].id not in to_remove:
            similarity = calculate_similarity(sequences[i].seq, sequences[j].seq)
            if similarity > 0.98:
                if len(sequences[i].seq) < len(sequences[j].seq):
                    to_remove[sequences[i].id] = True
                else:
                    to_remove[sequences[j].id] = True

    for i in range(len(sequences)):
        for j in range(i + 1, len(sequences)):
            executor.submit(compare_sequences, i, j)

filtered_sequences = []
for seq in sequences:
    if seq.id not in to_remove:
        filtered_sequences.append(seq)

with open(output_fasta_path, 'w') as output_file:
    SeqIO.write(filtered_sequences, output_file, 'fasta')

print(f'Processed {len(sequences)} sequences and wrote {len(filtered_sequences)} sequences
to {output_fasta_path}')
```

3. The python scripts for drawing virus genomic structures

```
import pandas as pd
import matplotlib.pyplot as plt
import numpy as np
from matplotlib.font_manager import FontProperties
```

the genomic structure information should be input as a CSV file with four columns: “chart_title”, “start”, “end”, and “name”, which refer to the name of the relevant virus, the start site of a gene, the end site of a gene, and the name of the gene, respectively.

```
# the file path should be replaced with your csv file
csv_file = 'C:\\Users\\Acer\\Desktop\\orffig_KM454473.csv'
df = pd.read_csv(csv_file)

if 'chart_title' in df.columns:
    chart_name = df['chart_title'][0]
else:
    raise ValueError("CSV 文件中不存在名为'chart_title'的列
")

rectangle_height = 1
rectangle_height_in_inches = rectangle_height / 2.54

fig, ax = plt.subplots()

ax.set_yticks([])
ax.set_yticklabels([])

for index, row in df.iterrows():
    if index % 3 == 0:
        y = 0
    elif index % 3 == 1:
        y = rectangle_height_in_inches * 2
```

```
        else:
            y = rectangle_height_in_inches * 4

        if index % 3 == 0:
            color = (0.906, 0.478, 0.325)
        elif index % 3 == 1:
            color = (0.020, 0.647, 0.620)
        else:
            color = (0.506, 0.243,0.596)


        ax.fill_between([row['start'], row['end']], y, y + rectangle_height_in_inches, color=color, alpha=0.6)

        text_x_position = (row['start'] + row['end']) / 2
        text_y_position = y + rectangle_height_in_inches + 0.2
        ax.text(text_x_position, text_y_position, row['name'], ha='center', va='top', fontproperties=FontProperties(size=10, family='Arial'))

title_y_position = -rectangle_height_in_inches * 1
plt.title(chart_name, loc='center', y=-0.5, fontproperties=FontProperties(size=14, family='Arial'))

ax.spines['top'].set_visible(False)
ax.spines['right'].set_visible(False)
ax.spines['left'].set_visible(False)
ax.spines['bottom'].set_color('black')

ax.set_xlim(left=0)

ax.xaxis.set_major_locator(plt.MaxNLocator(integer=True))
ax.xaxis.set_major_locator(plt.MultipleLocator(5000))
ax.xaxis.set_minor_locator(plt.MultipleLocator(1000))
```

```
fig.set_size_inches(30.48 / 2.54, 4 / 2.54)

fig.subplots_adjust(left=0.1, right=0.9, bottom=0.1, top=0.9)

ax.xaxis.set_major_formatter(plt.FuncFormatter(lambda x, pos:
'{:.0f}'.format(x)))
ax.xaxis.set_major_locator(plt.MaxNLocator(integer=True))

# the output figure file path should be replaced with yours
plt.savefig('C:\\Users\\Acer\\Desktop\\orffig_KM454473.png'
', dpi=300, bbox_inches='tight')
```

4. Alignment of three pairs of viral gene sequences

Those sites affected by frame-shift mutations are marked with color

3.1 Alignment of the 1a gene of a PdCoV and a DdCoV

```
PdCoV_1a    atggcaaatagccagaagaagggagtatctcccaatcaaaagaatgtcatctttgtagtg
DdCoV_1a    atggccccaggccttagcaagggagtatctcccaataaaaagaatgtcatttttgtagtg
            *****    .***  *. ****************** *************.*********

PdCoV_1a    aagggcattccacctgccttgtgtgacgcattgttcttttatacgtcacacattccaagc
DdCoV_1a    aaagacattcagccagcattgtgtgatgctttgtttttctatacatcacacgttccacgc
            **.*.***** .** ** ********.** *****.**.*****.******.***** **

PdCoV_1a    gactttgctgatgcatttgcttttcgtgagcaatttgatcgcaatttgcagaaaggtcgc
DdCoV_1a    gatttcgctgatgcgcataccgttcgtgaacggtttgatcgcaatttgcaaagcggtcgc
            **.**.********.. *.*. *******.*..*****************.*. ******

PdCoV_1a    gctttcaagtttgagactgtttgtggccctgtcatgttacagggtgtaccaacagtacca
DdCoV_1a    gtttttaagtttgagactgtgtgtggccctgtgtatttgcagggtgtaccctcagtccca
            *.***.************** ***********    **.***********  **** ***

PdCoV_1a    cctggtgtgaaagctcttgatgctaattctaagcttgctgatttagagcaagtttttggt
DdCoV_1a    ccaggtgtcaaggctttggatgctgaatctaagcttgttgatttggaaaatatttttggt
            ** ***** **.***.* ******.* **********.******.**. * .********

PdCoV_1a    gtgtcaccactgtcacgccgttatagagaactccttaaaaccgctgcccagtggtccctt
DdCoV_1a    gtttcacctctgtctcggcgttatagagaccttttgaagacggcaccacagtggtcgttg
            ** ***** ***** ** *********** **..* **.** **  * ******** .*

PdCoV_1a    aatgttgagtctcttgacgagcaggcaagtaagttggacactatttttaattcttctgaa
DdCoV_1a    aatgttgaatctcttgatgcccaggcggaaaaattggacactctttttagttcttcagag
            ********.********.*  *****... **.********* ******.****** **.

PdCoV_1a    attttgtggctacaggttgctgccaaagtacaagtgtctgcaatggcacttagacttatg
DdCoV_1a    attttgtggcttcaggttgccgctaaggtccaagtgtccgctatggcactgcgcagactt
            *********** ********.**.**.** ********.** ********  *      *

PdCoV_1a    gcaagtgaagtagtcaagaaaattgttagtacacttggtaatgaattttctaccatcttt
DdCoV_1a    gttggtgaggttactgcaaaagtcatggattcacttggctcaaatttgagcgctctgttc
            *. .****.** .... .***.*..* ..* *******.   .* **   ..*. * **.
```

```
PdCoV_1a        tctgttgttgttaatcaggtgaagaagatcattgacaagtgtctcactgtttttgagaat
DdCoV_1a        caagttgttaaggaaacaatggccagaatctttcaaaaggctttggccatttttgagagt
                .  ******.  .*   ..**.  *..*** ** * ***  *.* .*..*********.*

PdCoV_1a        gttgaagacctaccacaacgcatagctgccctaaaggctgcctttgcagcatgtgtgagg
DdCoV_1a        gtgagtgaattgccacaacgtattgctgcacttaagatggcttttgccaagtgtgcaagg
                ** .. ** .*.********.** ***** ** ***.. **.***** . .****..***

PdCoV_1a        cgcatgactattgtgattgtggataaggctctttgtattaaagagttagcaggtacttct
DdCoV_1a        tcaattactgttgtggttgttggacatgccctcactattagagagtttgctggaacttgt
                .  ** ***.*****.**** *.  * **.**.  *****.****** ** ** **** *

PdCoV_1a        cttgctagtgtgaatgcagctcttgcaacatggtgcaagactctgcccactggttttatg
DdCoV_1a        cttgcaagcattaatggtgcagttgcaaaattctttgaagaacttccaactggctttatg
                ***** **..* ****  **  ****** **  * ..*..  ** ** *****.******

PdCoV_1a        ggtgctaagatttttgacaacttggctttttataaagaagcggttgtcaaacttgttagc
DdCoV_1a        ggcgctaaggtcttttcaacccttgccttctttaaagaagcagcagtaaagattgtagaa
                **.******.*.***   * *.* **.**.* *********.*. ** **. **** ..

PdCoV_1a        aatgttgcaaatgcacctagtggtgtttcaggttatgtggtgattggtaaggcgcgttgt
DdCoV_1a        aatataccaaatgcgccaagaggtactaaaggttttgaagtcgttggcaatgccaaaggt
                ***.*  *******.** ** ***..*  ***** ** .** .****.** **  .  **

PdCoV_1a        acacaagttgttgtacgcggtatgcgctcagacctcaccttgttagatcaaaaagccaat
DdCoV_1a        acacaagttgttgtccgtggcatgcgaaacgacttaactctgcttgaccaaaaagctgaa
                ************** **.**.*****    ***.* **..**.* **.********..*

PdCoV_1a        gttcccgttgaaaaagagggatggagtgctgtttttgatggtaaactaagttacgtgttt
DdCoV_1a        gttcctgtggaaccagaaagttggtctgcaattttggagggacatctttgctatgtcttt
                *****.** ***  ***..* ***  *** .**** ** **  * **  *.**.** ***

PdCoV_1a        aagagtggtgattgttattatgcagcaccacttgcaggtaacgtagcgctcagtgatgtc
DdCoV_1a        aagagtggtgatcgcttttatgcggcacctctttcaggaaattttgcattgcatgatgtg
                ************.*.* ******.***** *** **** **. * **..*  .******

PdCoV_1a        tattgttgtgaacgcgtagtttaccttgcagatggttttacacctgaaataaatgatgga
DdCoV_1a        cattgttgtgagcgtgtggtcctccttagtgatggctacactcctgaaattaatgatggt
                .**********.**.**.**.. ****.  *****.* .** ******** ********
```

```
PdCoV_1a    ttgttgcttgctgcattgtatacatctaatgtagctgttgatgttttggctgcattgcgc
DdCoV_1a    ttgcttttggcagcgctttatacgtcatctactgtggctgatgtgttggctgcgctgaag
            ***.* .* ** **..* *****.**   *..  *.  *.****** ********..** .

PdCoV_1a    aaaggagagccatttaagtttttgggccactcttttgtgtatgttaagaatgcagctgtg
DdCoV_1a    aagggtgagccatttaagtttttgggacactcgtttgtctatgtcaaggacgcaactgtg
            **.** ****************** ***** ***** *****.***.*.***.*****

PdCoV_1a    tcttttactttggcaaaaggtgccactgtaggtgatgttcttaaactttttgttaaagaa
DdCoV_1a    tctttcactttggcaaagggtgcatctatagcagatgttcttaaactttttgttaaagaa
            *****.***********.*****  **.***  ****************************

PdCoV_1a    gcatctgatgtttggtctacttttgatgagaagtctcatgaattttggactagagcttac
DdCoV_1a    gctgccgatgtttggtctgtttttaacgaaaagtctcatgaattttggactagagctcac
            **  *.************..****.*.**.**************************.**

PdCoV_1a    aataagtgtcgtgacttgacagaacttgtcaaaacacactattgtaaagctcaagtgact
DdCoV_1a    aataaatgtcgtaaccttaaagaccttgtggcgacacactgttgtaaggcacaggtgact
            *****.******.**.* * *** *****  . .*******.******.** **.******

PdCoV_1a    tttgttgtacttgctgttgttttgggctctactacatgggagctttgtaagcaagttatg
DdCoV_1a    tttgttgtgcttgctatagctcttggtgtgacagtttggaaactcgtcaaacaggttttg
            ********.******.* *.*.* **.  .  ** ..  ***.*.**.   .**.**.*** **

PdCoV_1a    tttaaagtggcaggtctttttctgcagttgtagatttttgtaagagagcttgggatggg
DdCoV_1a    tttaaagtggctggtctttttacagccgttgtggatttttgtagtaaggcttggaatggg
            *********** ********* * ** *****.**********. *..******.*****

PdCoV_1a    ttttgtcaaaaactagcccatgtgaagcttgtaattaatgaagttggctgtgtgttgcat
DdCoV_1a    ttttgtgaccaactaatccatgccaaactctttgtgaatgaattgggctgtgttttggtt
            ****** *  *****..*****. **.**. * .* ****** * ******** ***  *

PdCoV_1a    ggaattaaagaacattgtttccaacttctttttgatgccttacacgcactttacaagact
DdCoV_1a    ggtgttaaaaaccactgttttcaactcttgcttgtagcagtccatgctttttataaaacc
            ** .*****.* **.*****.*****..* .***  **  * **.** .****.**.**.

PdCoV_1a    tttgacaagtgcgccataggtagaatttttgcaggtaacctgctgttttgggcaggaggt
DdCoV_1a    ttggaaaagtgtgccataggtagaatttgggccggtgatctactttttttgggctggtgga
            ** ** *****.****************  ** ***.*.**.** ******** ** **
```

```
PdCoV_1a    gttcacaaagtagatgaggacaactctgaagtttggtttgatgccattgatactgtacaa
DdCoV_1a    gctcacaaagtggaggaagacaatggtgaagtttggtttgacgctgtatacactgtacaa
            *.*********.** **.*****.  ***************.**..*  *.*********

PdCoV_1a    gttcaggatgaaggtacaattgtcgaaaacaatgtgcctgactttgtagcctgccctgat
DdCoV_1a    gttgacgaacaaggcactgtcattgaagacaacgtgcctgactttgttgtttgtcatgat
            *** * **  ****.** .*..*.***.****.************** *..**.* ****

PdCoV_1a    gtttctataccagagcgtcagaaaggctatcttgtgcagattatggctgatggtaaaact
DdCoV_1a    gtgtctatacctgagcgtcaaaagggatatatagtgcaggtttctgctgatggtaaaaat
            ** ******** ********.**.** *** * ******.** . ************* *

PdCoV_1a    tatatgtttttagatttcagcaagattcaaacacatactatgcacctatgtcccaagtg
DdCoV_1a    tatatgtttttaggtaccagcagggtgttaacacctattatgccccaatgtctcaggtt
            **************.* .*****.*.* . ***** **.***** ** *****.**.**

PdCoV_1a    ggtgctttgaatgttgtttgtagagctggtggtaagacagtaacatttggagatgaggaa
DdCoV_1a    ggtgcagttaatgttgtttgtaaggcaggtggtaaaactgtgacttttggtgaagaccag
            *****  * *************..** ********.** **.** ***** ** **  *.

PdCoV_1a    gtaacagaactcccaccaccagatgtgaagatgatatatatagacattgagtgttgtgga
DdCoV_1a    gtcaaagagctaccaactccagatgttaagtgtatttatcttgatattgagtgttgtggt
            ** * ***.** *** * ******** ***   ** *** * **.**************

PdCoV_1a    gaaccgtggactagtatcttcaagagagtttataaagaccctgttgaagtagagactaat
DdCoV_1a    gaaccttggactagtgtctttaagcgtgtttataaagacccagttgaagttgagacatct
            ***** *********.****.*** * ************** ******** *****   *

PdCoV_1a    ctcactgttgaagaattgaaatcttatgtctatggtatgatgtgtgacaacctaaaattg
DdCoV_1a    ttgactgtagaggagttgcgagcagttgtctatgagatgatgtgtgacagtttaaaacta
            .* ***** **.**.*** .* *   ********. *************...*****.*.

PdCoV_1a    ttgccagaagcaccacaaccaccggcatatgaaaatgtttcaattgttgacaagaaaggt
DdCoV_1a    tttccagatgctccaaaacctccgcattatgataatgttgccttggttgataaccagggt
            ** ***** ** *** **** ***   ***** ****** *  * *****.**  *.***

PdCoV_1a    caacttcttaaagttgtagattcttgtcgtctcgtctatgacgagtatgatagtgatggt
DdCoV_1a    cgtgatttgcaaaccattgattcttgtcatttggtttatgtggattatgacagtgatggt
            *.   *.*  **....* **********.*.* **.****  ** *****.*********
```

```
PdCoV_1a    gctgtgtcagatgatgactattgttcagactctgatgtggcagaagatgatgatgaagat
DdCoV_1a    g------------------------cagtttctgaagaagaagaagaagatgtgtcagac
            *                        *** .***** * .* ****** ****    ***.

PdCoV_1a    gtgcaggaagtggataatgatgatgataggctcgcaggacttttgaagaatcctgtgaac
DdCoV_1a    actgaagatgtcaatgaggaagacgaacgattggctggtttgcttaaaaaccctgccaac
            ..  *.** ** .**.* ** **.**  *..* ** ** .* .* **.**.****. ***

PdCoV_1a    tttaaatatccactaccttatgatgatgattatagtgtcttctgtggccaattggtgcat
DdCoV_1a    tttaaatatcctcttccttatgatgatgaatatagtgttttttgtgggcgtctcgttcac
            *********** ** ************** ********.**.***** *. .* ** **.

PdCoV_1a    agggacgctattgatacttatcaccacccatctggtgatgacatgtatgtagtcaacaat
DdCoV_1a    aaatgtgctatagacatttatcattatccatctggtgatgatatgtatgtcattaatgat
            *.. ..***** **.*.******..*.**************.******** .*.**..**

PdCoV_1a    tctgttgaaggtgctgtcaaagcaattcctcagaaggtttttgacttaatggatgattgg
DdCoV_1a    gcttttgatggtgccgttaaggcaattcctcagagtgttgttgatgttttaggagattgg
             ** **** *****.**.**.*************. *** ****. *  *.*. ******

PdCoV_1a    ggtgcagctgtggatgagcaagagagaagactcaacgcagaagagcctgaagtagtagtt
DdCoV_1a    gctgctgctgttgacagtcaagagcgcgctctactcgtagaaactcctgttgagactcct
            * *** ***** **... ****** * .  **   **.****.  ****  * ...  .*

PdCoV_1a    gtaagagatgttgctatactggatgtgaacaagggttcagagaatgtatttcaaaaactc
DdCoV_1a    aaggaagaaact---------------cagaagcctcaaaaggtggaagagcagaaacct
            . ...*** ..*                * ***  *. *.**.  * *   **.****..

PdCoV_1a    aacaccactctacctga-----------------------------aaacagtgagagtg
DdCoV_1a    aaggaaactcctgttgagactcctaaggaagaaactcagaagcctcaaaaggtggaagag
            ** .  ****.  .***                             *** .***..** *

PdCoV_1a    ctgtcagtaatgaaaatgc---------------agaagtggttttggaagagtctacag
DdCoV_1a    cagaaacctaaggaaactcctgttgagactcctaaggaagaaattcagaagcctcaaaag
            * *  * . * *.***. *               **.*. .. **..****  ** * **

PdCoV_1a    ttgaagtagacattgatgaaaaaaccacacctgtacaaaccactattgaaagtcatcctg
DdCoV_1a    gtg--------------gaagagcagaaacctaaggaaactcctgttgaga------ctc
             **              ***.*.   * ****. . ****. **.****.*      **
```

```
PdCoV_1a   ttgacgttgacattgatgaagctacgtctagcatagacggtagtatagaccagtctgcac
DdCoV_1a   ctaaggaagaaactcagaagcct-------------------------------------
           .*.* *  ** *.* * .*.  **

PdCoV_1a   aggaaaaccttgaggatcagagtcctgatgataatcatgtgcaagctgatgtaagtagtg
DdCoV_1a   --caaaaggtggaagagcagaaacctaaggaaactcctgt--------------------
              ****  * **.** ****. ***.* ** * ** ***

PdCoV_1a   aaaaagaggctgttatagaagagtgtgctgtagagcataccgttgaagaac---aacctc
DdCoV_1a   ----tgagactcctaaggaagaaactcagaagcctcaaaaggtggaagagcgaaaaccca
                ***.** .** .*****.  *   . .   ** *  ** *****.*   ****.

PdCoV_1a   aggattttgtttta---------------------------------gtagagtctaaga
DdCoV_1a   aggagactcttttaaataaacaatttctccctgatagttcgtctgatgatgaaccaagga
           ****  .* *****                                 *  **..* *.**

PdCoV_1a   agaggactcctaaatttaaattgaaaccaactaagtgtaaaccaccgaaaagtttagagt
DdCoV_1a   agaagtcttttagatttaaattgaaacctacaaaatgtaaagtgcccaataatgttgagt
           ***.* **..**.*************** ** **.****** ..** ** *.* * ****

PdCoV_1a   tttatacacatgttggtgagcttgatctagtcattgccaaggcaatggatttgtttgaag
DdCoV_1a   attctacatgtgtgggtgatttgtctgtagtcgttgctaaggcaatggactcctatgagg
            ** ****..*** ***** .*   * *****.****.***********.*. * ***.*

PdCoV_1a   actttgtcctagttaatgctgcaaatgagcatatggctcatggtggcggtgtagctaagg
DdCoV_1a   attttgttttggttaatgctgctaatgagcacatgcgtcatgccggtggtgtggcacagg
           *.*****..*.*********** ********.***  ***** .**.*****.**  ***

PdCoV_1a   ctatagcagatttttgtggacctaagtttgttgattattgtgatgcctatgtgaagaaac
DdCoV_1a   ccatagcagacttttgtggtaataagtttgtagattattgtgacacttttattagtaaga
           *.********.********   ********* ***********..*.* *.* *. **.

PdCoV_1a   atggcacacaacaacaactcttagctccttctggtgtagccaatattcaggctgttaaca
DdCoV_1a   atgggccccaacagcaattgttagccccttctggtgttgcgaagatacaagcagttaata
           ****  * *****.***.* *****.*********** ** ** ** **.** *****.*

PdCoV_1a   atgttgtgggacctagacaagggcaagacaacctttttgacaagttggttgaagcctatg
DdCoV_1a   atgttgttgggcctaggcatgggcagcatgacttgtttgataagcttgttgctgcataca
           ******* **.*****.** *****. *..**.* *****.***.* ****  ** **..
```

```
PdCoV_1a    aaagggttttggtcccaaatgcagtaaattatgccttgcctatcttgtcagttggcatct
DdCoV_1a    agaaggttgttgttcctggtgccattaactatgtggtgccggtcttgtcggcaggtattt
            *.*.**** * **.** ..*** .* **.****.  **** .*******.*. **.**.*

PdCoV_1a    ttggtgtagactataaacagtctatagatgctatgcgcaaggctcttgaagggaaaaatg
DdCoV_1a    ttggtgtggattacaaaatgtctattgatgcaatgcgcaaggcattttgtgaccttaaaa
            *******.**.**.***  ****** ***** *********** .** . *.    ** .

PdCoV_1a    ttcgtgtgttactttttactttgaagcaggaacacatcgaatatttcaatgcaagtctta
DdCoV_1a    ttcgtgtgctcttgttttctcttaatcaagaacacattgaatattttaatgccacttgta
            ********.* .* *** **.* ** **.********.********.***** * *. **

PdCoV_1a    agcagaagactgtttatcttacagaggatggtgtttcatataaatcacttgtccttaatc
DdCoV_1a    aacagaagggtgtttacctgacggaagatggcacgtcttttaaaactcttgttttgaaac
            *.******. ******.** **.**.*****... ** * **** * *****..* ** *

PdCoV_1a    ctggtgacacttttggtcacataggtggtgtttttgcgcgtaacaaaactatcttttcag
DdCoV_1a    caggtgacacattgggtcacctcggaggtgtttttgcaaagaacaagacagtttttacag
            * ******** ** ****** * ** ***********. . *****.** .*.*** ***

PdCoV_1a    ctgacgatgtcgttgataaagaagttttgtttgtgccaactaaaaacaaggcagttcttg
DdCoV_1a    cagatgatgttgaggacgaagaagttctcttcactccaacaacggacaaagctgttcttg
            * **.*****.*  **..********.* **... ***** * ..****.** *******

PdCoV_1a    actattatgatcttgatgctcaaaagtatgctttgtatttacgaacacttgcacaaaagt
DdCoV_1a    aatactatggattggatgcgcaaaagtatgtaatatatttgcaaacactttcgcagaaat
            * **.****. .* ***** **********.  *.*****.*.******* *.**.**.*

PdCoV_1a    gggaaatttttcaaaaagacaacattactcaactcgtttggcgagatggtaattgttggg
DdCoV_1a    gggaagtccaatatagggacaattttatcttacttaaatggcgtgatggaaactgctgga
            *****.*..  .* *..*****. ***... ***..  ***** ***** **.**.***.

PdCoV_1a    ttaattcagtagctgtactcctgcaggaagcaaagataaaatttaagggttatcttgcta
DdCoV_1a    ttagttcagctatggtgctccttcaggccgctaagattaggtttagaggttttcttgcag
            ***.*****. .. **.***** ****  ** ***** *..****..**** ****** .

PdCoV_1a    atgcatgggcacagttcttaggtggagaccctacagagtttgttgcatggtgttatgcta
DdCoV_1a    aggcatgggctaaattcttgggtggagaccctacggattttgttgcttggtgctatgcaa
            * ********  *.*****.**************.** ******** *****.***** *
```

```
PdCoV_1a    gtacaaacacacaagtaggtgaattttgtgatgccaatctctgtttgttgaatttggctc
DdCoV_1a    gttgcaatgctaaagttggtgatttttcagactgcaattggcttttggctaacttggcag
            **   **..*  **** ***** ****  **.  ****.  . **** . **.*****

PdCoV_1a    accattttgatgtagattacactaaagcttttatagctagacgtgtaacttgtaagtgtg
DdCoV_1a    aacactttgatgcagattacacaaatgcactgcttaagaagcgtgtgtcgtgtaactgtg
            * **.*******.********* ** ** .*  * .  *..*****. * ***** ****

PdCoV_1a    gagttaatactcagcacgtgtatggcattactgcctgtttgcagcatgttagtgctacta
DdCoV_1a    gtgttaagagttatgaacttagaggccttgaagcttgtattcaaccagtgagggctccta
            * ***** * *.*  *  *  . *** **.  **.*** * **.*  ** ** *** ***

PdCoV_1a    atatgttgaattttatgactcaatatggagattgtgataagtgtggtgagaagtatgttg
DdCoV_1a    accttctacattttaagacgcaatactcaaattgtccaacttgtggtgcaaatagtatgg
            *. * .*. ****** *** *****.  *.*****   *  ******* .**  .*.* *

PdCoV_1a    atgaagtcaccagcattaacttaccatacctgtttatgtttgtatcaggaggacctaaag
DdCoV_1a    atgaagttgttgaagcatcactcccgtacttgttgcttgttgctactgatggtcctgctg
            *******...... ..    .* **.***.****  *  ***.  * *. ** ***.  *

PdCoV_1a    ttgttgaacgtgataacaatgtcgttgctaatatagtctttttaggctctatagaaagcg
DdCoV_1a    cagttgattgtgatgaaaatgctgcaggaaatgttgtttttattgggtccaccaacagtg
            . ***** .*****.* ****..*. *  ***.* **.*** * ** **.*. .* **.*

PdCoV_1a    gacattgttatacacaaacacaacagggtgtctttgataatcttgttaataacagaaagt
DdCoV_1a    gacactgttatacacaagctgttggaaaagcttttgataatttggctcaagataggaaat
            ****.************.*     .... *..*********.* *.* * .*.**.**.*

PdCoV_1a    tttcaacaaaagcatcatatatgactgcttactacacacaagtttcctgt----------
DdCoV_1a    ttggaaagagatcgccttacattactgcaatgtatatgcgcttctctcttaagagtaaaa
            **  ** .*.* *..* **.** *****    **.*..*.  *.**.. *

PdCoV_1a    -----------------------------------------------gaaaaggaggttg
DdCoV_1a    attctctgtctgttgctaaaaagagtaagagcaagagtgatgtagtaaaagaagatgttt
                                                           .**.*.** ***

PdCoV_1a    ttgtagaggctagtagttcaggtgtggaagttgaagacgtttctgattttgagcgctggt
DdCoV_1a    ctaatctcgctacaagttcaaaattcagttttgatgatcttactgattttgaacagtggt
            .*.     ****  ******..  * ..  **** **. ** **********.*. ****
```

```
PdCoV_1a    attcggaaaattactatgagtgtcttactgtggaggatgttccacaagttgaagatg---
DdCoV_1a    atgatagtaacatctatgaaagtcttaaagtgcaggaaacacctgagaatatggatgagt
            **   .. **.  ******. ******  *** **** .. **  *.. *. .****

PdCoV_1a    ccatagattttacggattcagctgattcaattttaccattgactttgcctgttagaggta
DdCoV_1a    atgtgtcattcacaacaaaggaagattccaagttgccactgacacttaaagttagaggta
             ..*.   **.**..    .*  ***** *  **.***.**** .*    **********

PdCoV_1a    tagttaatgaaattaattttaagtcattgaatggttttacatacatcttgaaaccagtat
DdCoV_1a    tcaaatcagttgtcgactttaagtcaaaggatggctttacttataagttaatacctgaca
            * .     *  .*..*.*********  *.****.***** **.*  **.* *** *

PdCoV_1a    ctggagacagatgtgatgttcctgtatattatcctaccttagattccattagtcttagag
DdCoV_1a    ctgatgaaaattcaaaagcaccagtttactatcctaccttggacgctataagtcttaagg
            ***. ** *. *  .* *. ** ** **.***********.**. *.** *******..*

PdCoV_1a    ctatatggataaatggtgataataattatgttgttggccatcctaatagttatagtaggt
DdCoV_1a    caatatgggtggaaggtagtgctaactttatcgttggacatcccaactataatagtaaag
            * ******.*..* ***..*. ***.* *.*.***** *****.**. .* ******..

PdCoV_1a    gtcttcgtatacctactctctatgaggaggcagaacaatttatttcagtaagtgacagcg
DdCoV_1a    ttctgcgtgttcctacattctgggagtgtgcagagagctttgttaaaataggcgaaaaag
             *** ***.* ***** .***. *** . *****. . ***.**  *.**.*.** *. *

PdCoV_1a    ttggtaatgtgacttatggcaaatggtctgccaaacagctaaacaaacccatgctgtctt
DdCoV_1a    ttgatggtgtaactatgggcctctggcgtgcagaacaccttaatagacctaatttggaaa
            ***.*..***.***   ***   ***. *** .**** ** **.*.***.*  .**

PdCoV_1a    ctgttataactgttattaagaa------------cattattggtgtcgcaacacccattt
DdCoV_1a    gaattttcaacgtcgttaagaagacgatggttggaactagtgttgttactacacaatgtg
              .** * * .**..*******             *.** ** ***..* ****    *

PdCoV_1a    caacagttgttaaggaaaca---------gtcactaatacagtgcgacgtagtcttaact
DdCoV_1a    gtaaattaattagtaaagcagctacattcgttgccgataaagtaggagatggtgtagttc
              * * * .***. .**.**         **..*..*** ***. ** .*.** * . ..

PdCoV_1a    ataatgttgtgggtaatgtggtaggttctattttaggttttgtaggtgactcttttaaac
DdCoV_1a    gcaatgtttctgatagaattaaagg--gtgttttgggtctactagggagcattttgaaa-
            ..****** . *.**. .* . ***   *.****.***.*  ****  ..* .*** ***
```

```
PdCoV_1a    gcattcgcggtaaaatctcttttcatctattaaaaaccatattactcttcttgttttact
DdCoV_1a    ---------gaagagtgtctccgcagttcttaaaaaccctctttttcttcgtcttttgtt
                     * *.*.* ***..  ** .* ********* * ** .***** * ****..*

PdCoV_1a    tttgtaaagcaagtttttcaagatttacatgtaattatcgtggtgttgtaaccaaagttg
DdCoV_1a    ttttgaaggctagtgttaagggtttaatggctagctacaagagtgtgttatgtaaagtgt
            ***  **.** *** **  ..* ** *..   **..**.  . .****  **  .*****

PdCoV_1a    ttatggcatcatttttcttaacttggttcttttacacctatagtgcaatgcattttgtag
DdCoV_1a    tatttactgctttacttatattgtggattgtctacactagcgacccagttatatctactg
            *  * .*  * ** .*. ** .  *** *. *.*****. .....  **.*    *.*.. *

PdCoV_1a    gtgtaaaagtattaaatttcttatttgaaggttccttatgtaatagttataaggattatg
DdCoV_1a    gaatacgtgtgttagatttcctatttgaaggttcattttgtagtccttatgcggattatg
            * .** .  **.***.*****.************* ** ****.*  ****.  ********

PdCoV_1a    gtaaagaaaccttcaatatgttgcattattgtggcgacgatgctgtgtgtagagtctgcc
DdCoV_1a    gtaaagagtcttttgatgtgttacgctattgtggaagtgactttactcgtcgtgttcgtt
            *******.  *.**..**.****.*..******** ...**.  .*..  .** * **..*..

PdCoV_1a    tacatgatgtagattcactgcatttgtataaacatgcttatagtatagagcaggtttata
DdCoV_1a    tacatggtaaggattctcctcatttgtataaacatgcctatagcgtagcacagttttata
            ******.*.  .***** *.  ****************.*****..*** .*** ******

PdCoV_1a    agaatgctgtagtacaatttaattttacatggaattggttttactttttgtttttattgt
DdCoV_1a    aagatgcagttaatggtatcagttttacatggaattggctttatatgctgtttctactat
            *..**** ** .     .   *.*.***************.****.  * .*****.**.*.*

PdCoV_1a    ttttcgtaaaaccagttgcactttttattatagtctgttattgtctgaagtatttagttt
DdCoV_1a    tgtttgttaagccagtagcaggttttgttattatttgctactgtataaggtacttggttt
            * **.** **.***** ***   ****.**** .*.**.**.*** *.*.***.**.****

PdCoV_1a    taaatgctgatttttttacagactggtataagtgtctttgactggtgtcttgttactttct
DdCoV_1a    tgagcacaactgttttacaaactggtgtaggattcttagactggtttatccaaacagtgt
            *.*...* .  * *******.******.**.*   **** ******* * *.    **   * *

PdCoV_1a    ttagtaactttaattttatgggtgcaggattttatgtttggatcttatggcaaatatata
DdCoV_1a    ttgctaactttaattttatgggtgcaggattttatttctggctttttataaggtatata
            **.  ****************************** *.*** *.** *.   *..******
```

```
PdCoV_1a    aatatgcacaccatattatttattgtaaggatgtaacctgtgacatatgtaggcgtgttg
DdCoV_1a    tacaggtgcatcatattatgtactgtaaggatataacatgtgaagtgtgtaagcgagttg
             *.* *..**.******** **.*********.**** ***** .*.****.*** ****

PdCoV_1a    cgcgtaacagtcgtcatgaagttagtgttgtagtaaatggacgtaagcagtcagtttatg
DdCoV_1a    cacgtagtaatagacatgaggttagtgttgttgttaacgggcgtaaacagcttgttcacg
            *.****..*.* * *****.*********** ** **.**.*****.***.. ***.*.*

PdCoV_1a    tgtataccaattcaggttttacattttgtaataaacacaattggtattgtaaagattgtg
DdCoV_1a    tctacactaactcaggctatagtttctgtaagagacataattggtattgtagaaattgtg
            * **.**.**.*****.* **  **.***** *.***.************.*.******

PdCoV_1a    ataagtttggtcaccagaacacatttatttcacctgaagtagcgggtgaactttctgaca
DdCoV_1a    ataagtatggccaccaaaatacatttatgtctcctgaagttgctggcgagctttctgaaa
            ****** ***.*****.**.******** ** ******** ** **.**.******** *

PdCoV_1a    aacttaagcgccatataaaacctacttcacacgcttattatgttgtggatactgcacaat
DdCoV_1a    agcttaagcgccatgttaaacctacagcattcgcataccacgtagtggatgatgcatgct
            *.************.* ********  **. *** **..*.** ******. ****.. *

PdCoV_1a    tagttgatgagggttttgtaaactttaactaccgcagttctgaacctggtagtggtgtga
DdCoV_1a    tagttgatga---ctttgttaatttgaagtataatgctgccactccaggtaaagaaggtg
            **********   .***** **.** ** **. ... * *..  ** ****. *. *  .

PdCoV_1a    aaaaacctcaggttaagtgctttacggttgcagaatttttgaaaaatgctgtttttctta
DdCoV_1a    tgcattctgctgttaaatgttttagtgtatctgatttcttaaagaaagctgtttttctta
             . * .**   *****.**.****  **  * ** **.**.**.** *************

PdCoV_1a    aagatgctgtaaagtgtgattttatagaaaacaatggttttattgtttgtaatacacaga
DdCoV_1a    aggatgcacaaaaatgtgagcaaatatctaatgatagttttatagtttgtaatacacaca
            *.*****   ***.***** .  ***   **..**.******* ************** *

PdCoV_1a    gtttgcatgatttagaggccgctaagaatgttgctgtctattatgcacactatttgtgta
DdCoV_1a    gtgcgcatgcattagaggaagcaaaaaatgctgccatctattatgcgcaatgtctctgta
            ** .*****  *******  ** **.****.***..**********.** *.*.* ****

PdCoV_1a    aacctattcttattcttgaccaaattctatataatactctggctacacaaccagtttcaa
DdCoV_1a    aaccaattcttatacttgaccaagtgctttatgacactttggcttgtgaacctgtgtcta
            **** ******** *********.* ** ***.*.***.*****    **** ** ** *
```

```
PdCoV_1a    agaatttggtggacaagatttctggtgttttggctagtatagttagtgtaaata------
DdCoV_1a    agagtcttgttgacaaagtgtgtggtgttttggccggcatccttgatgttgataattact
            ***.*.* ** *****..* * ************..*.**  **..*** .***

PdCoV_1a    ------ttgaaaacataaactataatgctgggtctttgcgtgatgctttgttgtctatta
DdCoV_1a    gtttttctggtaaactcaattataaagccggttctcttcgtgatgccctcttgtctgtta
                  .**. **  * **.***** **.** ***.* ********..* ******.***

PdCoV_1a    ctgacgatgaagaagctgttgatttggctatcttttgtcataattatgatatcgcttata
DdCoV_1a    cggcaggtgaagaggctgttgatatggctatgttttgtcataattatgaccttagttaca
            * *  *.******.********* ******* *****************. *.. ***.*

PdCoV_1a    cgcaagatggttttactaacattgtgccttcatatggtattgatgttagtaaattaacac
DdCoV_1a    ctagtgatggttttactaatgttgttccttcttatgggtttgaccttggtaaactgacac
            *  . **************..**** ***** *****  ****. **.*****.*.****

PdCoV_1a    cgcgtgataaaggatttttagttaacgctgacgcttctattgctaatcttaaagttagaa
DdCoV_1a    cacgtgataaaggttttcttataaatgctgatgcgtgcatagctaatcttaaggttagaa
            *.*********** ***.* .* **.*****.** * .** ***********.*******

PdCoV_1a    atgcgccagcagttgtttggaattaccatgacttgtgtaaattgtctggtacttgtctta
DdCoV_1a    atgcaccacctgtggtgtggaattatcatgaccttataaagttgtctgataattgtctta
            ****.*** * ** ** ********.******.*    **.*******.** ********

PdCoV_1a    aatacttaatctctgctactgtaaaggcaggtggtaagttttatgtaacgagatctcttg
DdCoV_1a    agtttttagtgtctggtgttataaaatcaggagctaaattttatgtaacacgttctgatg
            *.* .***.* **** *..*.****. **** * ***.***********. * ***  **

PdCoV_1a    ttaagcaagttattacttgtcatacagagaaacttattttggaaaagaaagctggtggag
DdCoV_1a    ttaaacaagtcattacttgtctaacacagaagttgttgttagataagaaggccggtggtg
            ****.*****.**********  *** ****..*  * **.** *****.**.***** *

PdCoV_1a    ttattaaaaacactgcttcttatttttattttgtttttaagttctttttagtactttatt
DdCoV_1a    ttcttaaggatactatgtcttggttttggtttatagttaaatttgttgttgtgtgttatc
            ** ****..*.***.. ****. ****. ***.*  ****.**. ** * **.. ****.

PdCoV_1a    tgattgtttctgcaggttg------tgtttattatcatgctaatagtggtggttttatac
DdCoV_1a    ttatttttacagcaggttgtttgtatgtttatcatacagattattatggtgttaagatgc
            * *** ** * ********      *******.**   * * ** .***** *   **.*
```

```
PdCoV_1a   acactatgtatgatgttaacaacacttttcctgttgacaattataaagttatagagaatg
DdCoV_1a   acccgatgtatgatgtgaattcaacctttcctgttgagaattttaaggttatagaaaatg
           ** * *********** **.   **.*********** **** ***.********.****

PdCoV_1a   gtgttattagagatattgtttctgaggacaattgtttctctaataaattttctacttttg
DdCoV_1a   gtgtccttagagacctggtttcagaggacaattgtttttcaaataagttttgggttttg
           ****. *******. * ***** **************.** *****.****. . *****

PdCoV_1a   ataacttttggggaagaccttatgttaatagtagggattgtccaatagttacagcaatta
DdCoV_1a   atgacttttggggcaaaccttatactaatagtagggattgtccaatagttactgctctta
           **.********** *.*******..*************************** **  ***

PdCoV_1a   tagaaggtgcaggcattgtagcagcaggagtgccaggtcatgtccaatgggttttggatc
DdCoV_1a   tagatggtaccggttttgttgcggcaggtgttcctggttatgttcattgggttatggaca
           **** ***.* **. **** **.***** ** ** ***.****.** ****** ****.

PdCoV_1a   gaactatgttcattcatactacccagattgagataaaaccatggtatgcacctagttggt
DdCoV_1a   ataccatgtttatacacactgcacaagcagaacaaaaaccttggtatgcacctagttggt
           . **.*****.** **.***.* **... **.  ****** *******************

PdCoV_1a   ttccacatgataatgtggtaggttatacaactgattctataataactcaaggtgagtttt
DdCoV_1a   tcccccaggaggatgtagttgggtatactattgactctgttattacacagggtgaatttt
           *.** ** ** .****.** ** ***** *.***.***.* ** ** **.*****.****

PdCoV_1a   accaatcaatagccactatgcctgctaggtgtatgtatttagctagtagtggtattcaac
DdCoV_1a   atagctcaattgcgacgtttcctgcacgttgtatgtattttgcaacaagtgcggcgcctc
           *. . ***** ** **  * *****  * *********** ** *  ****  .. *  *

PdCoV_1a   agctttattgttatggaggtgaaaatgatgcccctggtgctatttcttatgagagcattc
DdCoV_1a   aattgtattgttatggtggtgaaaatgatgctccaggtgctatgccatatgagagtgttc
           *..* *********** **************.** ******** .* ********..***

PdCoV_1a   agcctcaccgcgtttacctccaaccaaatggaatacggttttctataccgcaacagttat
DdCoV_1a   aacctcatcgtgatattttccaaccaaataatgttaggttttctattcctcaacaaatta
           *.*****.**.* *  ..***********.. .*  ********** ** *****. *

PdCoV_1a   tatatacaccttatatagttaaattgtcttctgataattactgtaaaggtagtgtttgtg
DdCoV_1a   tgtacacaccctatatagttaagttggcttctgacaactattgtagaggtagtgtttgtg
           *.**.*****.***********.*** *******.**.**.****.**************
```

```
PdCoV_1a   aaaagactaagttaggctattgtttttcatggaaccctcgttgggtgttgtataatgatg
DdCoV_1a   aaaagacaaagcttggttattgttattcatggaaccctcgttgggttctttataacgatg
           ******* ***.* **.******* ******************** .* *****.****

PdCoV_1a   attacactggcctatctggtgtttattgtggttctactattagggaacttttatttgtta
DdCoV_1a   actatgttaagttgcctggtgtttattgtggggccactgttagagaacttgtgttttcta
           *.**...*.. .*..****************  *.***.****.****** *.*** .**

PdCoV_1a   tggttaattcatttttacaggagttaaccctaatatgtatttacacctaactacaatgt
DdCoV_1a   tgttgggctcatttttacaggcgttagccctaatttgtatttgcacctaacaactatgt
           ** * ...************** ****.******* *******.******** ** ****

PdCoV_1a   tttttgtattagctgctgtggtgttttgttttatgattatgatacgctttcaaggtgtgt
DdCoV_1a   ttttggtattgttgtgtgttatggttgtgtttgcactggttattaggtttcaaggtgttt
           **** *****. .    *** .** **   ***... * .* **  * *********** *

PdCoV_1a   ttaaagcgtatgcatcaatagtctttacaatcataactgtatgggttgtgaatgtgttta
DdCoV_1a   ttaaagcctatacaccagtcgtttttgcagtcattatggtttggggcctgaatgtcttta
           ******* ***.**.**.* **.***.**.**** *.  ** **** .  ******* ****

PdCoV_1a   tgttgtgtgtttatagttataatcctttgttgcgattatactagtagcactttattgtt
DdCoV_1a   tgttgtgtgtctatagctataaccctttgtagcggttatgttgttggcactttattgtt
           **********.*****.*****.******** ***.****..*.  *.*************

PdCoV_1a   attgttcactcatggtaagtaagaatgtagctgctattatgcatctatgggtgatcttta
DdCoV_1a   atatgtcactcatggttggacgtaccgtttctacggttatgcacctgtgggttgtattta
           **   *********** .*  .  * .**  **.* .*******.**.***** .* ****

PdCoV_1a   catttgtcttagtagtcccttggtggattacatgtttttatatagtttttgctatttata
DdCoV_1a   cttttataatggttgtaccttggtggttggcctgtgtttatatagcttttattgcttata
           * ***.*  *.** ** ********* * .* *** *********.****..*..*****

PdCoV_1a   tgtataccccttttgttttgtggttttatggtactgcaaaacaaactcgtaaactttatg
DdCoV_1a   tgtacactccatttgccatgtggtgttatggcactgccaaacgaactcgtaagctttacg
           ****.**.** ****..  ****** ******.***** ****.*********.*****.*

PdCoV_1a   atggtagtgagtttgttggtacatatgatatggcagctcaaagtacatttgttatacgta
DdCoV_1a   aaggtaacgagtttgtaggcagttatgatttggcagcccagagtacgtttgttatacgta
           * ****..******** **.*  ****** *******.**.*****.*************
```

```
PdCoV_1a    atgttgagtttgtcaaattgtctaatgagataggtgataagttggatgtttatttgtctg
DdCoV_1a    atgttgagtttgtcaaactttctaatgaagtcggtgaaaagcttgacgtttatttgtcag
            *****************.* ********..* ***** ***.* **.*********** *

PdCoV_1a    cttatgcccgtcttaaatattactctggcactgggggtgaacaggattatttacatgctt
DdCoV_1a    cctatgcacgactaaagtattattcaggcactggcggtgagcaagactatttgcatgctt
            *.***** ** ** **.*****.** ******** *****.**.**.*****.*******

PdCoV_1a    gtcgcgcatggttggcttatgccctagatcaatatcgttctagtggcgtagaagttttat
DdCoV_1a    gtcgtgcctggttggcttatgctttggatcaataccgtgctaatggtgttgaagtgttgt
            ****.** **************..*.********.*** ***.***.** ***** **.*

PdCoV_1a    atacaccaccaaagttttctatgggcgctagtaggttacaagctggttttaaaaagtttg
DdCoV_1a    atacaccacctaagttttcaatgggtgttagtaggttgcaagcaggctttaagaagtttg
            ********** ******** *****.*.*********.***** **.*****.*******

PdCoV_1a    tttcgcctagtagtgtggtagaaaagtgtgtggttatggttcgttatagaggtaatgtcc
DdCoV_1a    tttctcctagtagtgccgttgagaagtgcattgttagtgtatcctataggggtaataatc
            **** **********. ** **.*****..* ****  ** . .*****.******. .*

PdCoV_1a    ttaatggattatggcttaatgattctgtatattgcccaagacatgttatgggtaaatata
DdCoV_1a    ttaatggattgtggttgggtgactccatctactgtccacgtcatgtgttaggtaagttta
            **********.***.* ..***.**..* **.**.*** * *****  *.*****.* **

PdCoV_1a    gtggtactgaatggcaagatgtactcaacttagctaacaatcatgaatttgaaattgtta
DdCoV_1a    gcggtgaccaatggggtgatgtacttaatcttgctaataatcatgagtttgaagtagtaa
            *.***. . ***** . ********.**..* *****.********.******.* ** *

PdCoV_1a    gctcagatggtgcaactttacatgttgtcagtaggaagttacagggtgcagttcttgttt
DdCoV_1a    ctggaaatggtgttactttgagtgttgtcagtaggcgtttgaaaggtgcagttttaattt
             .  *.******. *****. .************* . **. *.*********.* .***

PdCoV_1a    tacaaacagcaactgtaaatgttaacactcctaagtataaatttgttcaagctaaatgtg
DdCoV_1a    tacaaactgcaattgtcaatgctgatacaccgaagtataaatttttgaaagcaaattgtg
            ******* ****.*** ****.*.*.** ** ************ *  **** ** ****

PdCoV_1a    gtgatacttttaccattgcttgttcttataatggtaatgtcgttggcttgtatcctgtta
DdCoV_1a    gtgatagtttcacaatagcttgctcttatggtggtatagttataggactctaccctgtta
            ****** ***.** ** *****.******..*****  **..* ** .* **.*******
```

```
PdCoV_1a    ccatgcgttctaatggaaccgtaaaggcatcattttcttgtggttcctgcggttctgttg
DdCoV_1a    ctatgcgttctaatggaactattagagcatcttttcttgctggagcatgtggttcagtag
            *.*****************..* *..***** ***..*  ***  * **.***** ** *

PdCoV_1a    gttatgtaatgagtggtagtgttgtaaacttttgttacatgcaccacttagagcttccta
DdCoV_1a    gttttaatatagaaaagggtgtagtaaatttttactacatgcaccatctagagttaccta
            *** *.  **... .. .**** *****.****..***********..*****.* ****

PdCoV_1a    atgctatacatacgggtactgattttgcaggtaacttttatggtggttttgttgatgaag
DdCoV_1a    atgcattacacacaggaactgacctaatgggtgagttttatggtggttatatagatgaag
            ****  ****.**.** *****..* ...***.* ************* *.* *******

PdCoV_1a    aaaaagctcagcgtgttgagcctgatctgttaattactaataatatagtagcatggcttt
DdCoV_1a    aggttgctcagaaagttcaacccgataaattagttactaataatattttggcatggcttt
            *..  ****** . *** *.**.***  .***.*************  *.**********

PdCoV_1a    atgctgctataattagtgtaaaagagaacacaataaccactccttcttggcttgaaaaag
DdCoV_1a    atgcagcaattattagtgttagagagagtagtttttcaacaccaaaatggcttgaaagta
            **** ** ** ******** *.*****..*   *  * ** **    **********. .

PdCoV_1a    tgtctgttagtgttcaagattataataagtgggctgctgatagtggtttcacttcttttg
DdCoV_1a    ctactgtcagtattgaagattataataagtgggcagtggataatggttttacatcatttg
            .  ****.***.** ****************** *. ****.******.** ** ****

PdCoV_1a    ttgacaatgtggctattaataaattggctgttttgacttcaattgatgttggtaagattt
DdCoV_1a    taagctgcactgctattactaaattaagtgctataacaggagttgatgtttgtaaactcc
            * ..* .... ******* ******.. **.* *.**   *.******** ****. *..

PdCoV_1a    tgcgtaccattatagttaagcatacccattggggccaggaagctataatgggtttatata
DdCoV_1a    ttcgtactattatggtaaaaagtgcacaatggggtagtgaacctattttgggacaatata
            * *****.*****.** **. .*.* ** *****. . *** ****  **** . *****

PdCoV_1a    attttgaagatgaactaacacctgaatctgtttttaatcaagtaggtggtgttaagttgc
DdCoV_1a    attttgaggatgaaatgacaccggaatctgtttttaatcatgtgggtggtgttaggttac
            *******.****** *.***** **************** **.**********.***.*

PdCoV_1a    agtctacttttgtgaaaagaacattgtcatggttttggtctaggacaatattagctttat
DdCoV_1a    aatcttctgttgtaaagagagccgcatcttggttttggagtacatgtgtgctattttgtt
            *.*** ** ****.**.***.*  ..** *********  ** .   .*..** .**  *
```

```
PdCoV_1a   ttttgtttgtattgagtgcaatggttctttttactgttgtaccctataaatattttattt
DdCoV_1a   gcttgtttgtcttgtgttctatagttctgtttacagctataccatatagatattatttac
            .******** *** ** * **.***** ***** *.*.**** ****.***** * * .

PdCoV_1a   acgtggccgtaatgttgttaattgttgtatttttggctgcatttactataaagcatgtat
DdCoV_1a   atggtgctgttgttttatttgcaactgtgctctttgtgtcgtttactgtgaaacatgtta
           *.*  **.** .* **.** .. ..***..*.** *.  *.******.*.**.*****  

PdCoV_1a   tggctttttttggatacatttttattgccaacattgtgtacggtcatgattggtgtttgtc
DdCoV_1a   tggcatatatggataccttccttttgccgacattgattacggttattatagggg tttgtg
           **** * * ******* **..* *****.******  ******.** ** ** ****** 

PdCoV_1a   tcgaagttcctcttgtgtataatagtt--atgtgcaagattttcttcgtagtttgtttat
DdCoV_1a   ctgaagtgcctttcatctataatactctaattagtcaaattgt-----tattttctttag
           ..***** ***.*..* ******* *.  **  *. *.*** *     ** *** **** 

PdCoV_1a   ttcatggtataatcctgaagttatagacactgttgtaccttggctttttacacctataat
DdCoV_1a   ccaatggtatgatccagtagtctttgacactgtagtaccgtggatgtttctaccattagt
           .. *******.**** * ***. * ******** ***** *** * *** .***  **.*

PdCoV_1a   cttgtatacaactatgaagtgtatacagggttgttgtgctataaattctctgtcgactgc
DdCoV_1a   cttgtacacagcttttaaatgtatacaaggttgttatagtgtaaattcctttaatacttc
           ******.***.** * **.********.*******.*. *.*******..*    *** *

PdCoV_1a   attaattggtgtttaccaagtatttaagctagcctttatggcatatacggcttggaatac
DdCoV_1a   tctgttagtgttgtatcagtttatgaagttgggttttgttatatatacctcttctaatac
            .*. * *   * **.**. *  * ***.*.* .***.* ..******  ***  *****

PdCoV_1a   tggtcacgcatatagtgcaggtaattgggagctattttttgagctgttgcacacaacagt
DdCoV_1a   tcttacagcatattcagaaggtaactgggaactattctttgagttagtgcacacaactgt
           *  *   ******   * ******.*****.*****.******.*. ********** **

PdCoV_1a   tttggctaatgttagtagcaattctattgtaggattgcttgtttttaatgttgctaagtg
DdCoV_1a   gttggctaatgttagtagtaattctttaattggcttaattgtgtttaaatttgctaagtg
            *****************.****** * .* ** **. **** *****  **********

PdCoV_1a   gtgtttaaattattgtaacctcacttatcttaattcatatgtgcttatggctatatttat
DdCoV_1a   gatgctgtattattgtaatgcttcataccttaataattatgttctaatggctgtcattat
           *   .*. **********. .. * **.******   ***** ** ******.*  ****
```

```
PdCoV_1a    taatgtcataggttggttgtttacatgttattttggtgtttattggtggcttaacaaagt
DdCoV_1a    taatggcataggttggatgtttacttgttactttggattctattggtggattaataaggt
            ***** ********** ******* *****.*****  *.********* ****.**.**

PdCoV_1a    ctttggttttacttttggtaagtacacttttaaagttagtgttgaacaatataagtatat
DdCoV_1a    ttttggtttaaccttaggtaaatatagtttcaaagtttcagtagatcaatataggtatat
            .******** **.** *****.**.* ***.******   ** ** *******.******

PdCoV_1a    gtgtgtgcataagataccagcacctaaaacagcttgggatatcttttcaacaaatatact
DdCoV_1a    gtgtcttcataagattaatccacctaaaactgtgtgggaagtcttttctacaaatatact
            **** * ********     ********** *. ***** .******* ***********

PdCoV_1a    tatacagggtataggtggtgataggaaacttcctatagctacagtacaatctaaacttac
DdCoV_1a    tatacaaggtataggtggtgaccgtgtgttgcctatagctacagtgcaatctaaattgag
            ******.**************. * . ..* **************.*********.* *

PdCoV_1a    tgatgttaagtgtactgccgtagtgttaatgcaacttttgactaagcttaatgttgaagc
DdCoV_1a    tgatgtaaagtgtacaactgttgtcttaatgcagcttttgactaagcttaatgttgaagc
            ****** ******** .*.** ** ********.**************************

PdCoV_1a    taattcaaagatgcataagcacttagtagaattgcacaacaaaattttagcttcagagga
DdCoV_1a    aaattcaaaaatgcatgcttatcttgtagggttacacaataaaattcttgcctctgatga
             ********.******.  .*..* ****..**.*****.******.* **.** ** **

PdCoV_1a    tcttgttgaatgtatggatcatttgttaggtatgcttgttaccttgctttgtatagattc
DdCoV_1a    tgttaatgagtgcatggacaatttgttgggtatgcttgtaacactattctgtgtagatag
            * **. ***.**.*****. *******.*********** ** .*..*.***.*****

PdCoV_1a    aactgttgatttgagtgagtattgtgaggatgtgctctctaatgttacagttttacaatc
DdCoV_1a    tactatcgatttaagtgagtattgtgatgatatacttaagaggtcaactgttttacaatc
             ***.*.*****.************** ***.*.**.   *.  . ** ***********

PdCoV_1a    tgtaacacaagagttttctcacataccatcatatgcagagtatgaacgcgctaaagaatt
DdCoV_1a    agttactcaggagttttcacacattccctcctatgcggagtacgaaagagctaaagatct
             ** ** **.******** ***** ** ** *****.*****.*** * ******** .*

PdCoV_1a    gtatgagcgcgttcttgctgaatttaagaacggcaatgttacacaacaagaagttgctgc
DdCoV_1a    ttatgaaaaggttttagctgattctaaaaatggtagtgttacacagcaagagcttgctgc
             *****. . ***.* ***** *.***.**.**.*.*********.*****. *******
```

```
PdCoV_1a    ttatcgtaaggctgctaatatagctaaatctgtttttgatagagatctttcagtacagaa
DdCoV_1a    atatcgtaaagctgccaatattgcaaagtcggtttttgatagagatctggctgttcagaa
             ********.*****.***** ** **.** *****************  * ** *****

PdCoV_1a    gaaattagacgctatggctgagcgtgctatgactaccatgtataaagaagcccgtgttac
DdCoV_1a    aaagttagatagcatggccgaacgtgctatgacaacaatgtataaagaagcgcgtgttac
            .**.*****.. .*****.**.*********** ** ************** ********

PdCoV_1a    tgacagacgtgctaaattagtctcatcattacatgcgcttttgttttctatgttaaaaaa
DdCoV_1a    tgatagaagggcgaaacttgtttcatcattacatgcgctattattttcaatgcttaagaa
            ***.*** * ** ***.* **.***************** **.***** ***.* **.**

PdCoV_1a    gatagattctgataagttaaaatctctctttgatcatgctagagatggtgttgttccact
DdCoV_1a    gatagattctgaaaagctcaatgtactatttgatcaagcaagcagtggtgttgtaccgct
            ************ ***.* **  . ** ******** ** ** ..********* **.**

PdCoV_1a    tgcaactgtaccaatagtttgtagtaacaaacttacgttggttataccagatgcaaatac
DdCoV_1a    ggctactgttccaatagtttgtagtaataagcttactcttgtagtgccagacccagaaac
             ** ***** *****************.**.***** .* ** .*.*****. **.* **

PdCoV_1a    gtggaacaagtgtgtacaaggtagttatgttacctattcaactgtagtatggaacattga
DdCoV_1a    atgggtcaagtgcgtagagggcatgcttgttacttattccacagttgtttggaatattga
            .***. ******.*** *.**.*  . ******.***** ** ** ** *****.*****

PdCoV_1a    ctctgtactagatgctgatggtgttgaacaacaacctatttctgatggtcaaaacttaac
DdCoV_1a    tactgtttttgatgctgatggtgttgaaattcaacctagttctaacgggcaaagtcttgt
            . **** .* ******************   ******* ****.*.** ****...* ..

PdCoV_1a    gtacccaatctttggtgataaggtggcttggcctcttaaagttaatcttgtgcgcaatgc
DdCoV_1a    ctaccacataggtggtgacagaattgcttggccacttaaagttaatcttacgcgtaatgt
             ****  **   ******.*...* ******** ***************..***.****.

PdCoV_1a    tcataataaagtagatgtttcactacagaataatgagttgatgccacaaggtgtaaaaac
DdCoV_1a    tcataataaagtggaggcttctctgcagaataatgaacttatgccccaagggataaagac
            ************.** *.*** **.***********..* ***** ***** .****.**

PdCoV_1a    taaggcttgtgttgctggtgttgatcaaacatcttgtaacgtagagtctaagtgttatta
DdCoV_1a    taaggcttgtattgcagggactgatcaagctcattgtaatgtagagtctaagtgttatta
            **********.**** ** ..*******.* . ******.********************
```

```
PdCoV_1a   tactaatattaatggtagcacagttgtagcagccattacatcatctaatcctaatttgaa
DdCoV_1a   tactaatattaatggtagttctgttgtagctgccattacatctgataatccaaatttgaa
           ******************. * ******** ***********   ****** ********

PdCoV_1a   agtagcatcatttttaagtgatgctggtaatcagatatttgttgacttggatccaccttg
DdCoV_1a   agttgcatcattcttgagtgatgctggcaatcaaatttttgttgatcttgacccgccatg
           *** ********.**.***********.*****.** ********..* **.**.** **

PdCoV_1a   taaatttggtatgaaagttggtggtaaagtcgaagttgtgtacttgtatttccttaaaaa
DdCoV_1a   taaatttggtatgaaagttggtggtaaggttgaggttgtttatctttatttcttaaaaa
           ***************************.**.**.***** **..* *****.********

PdCoV_1a   tacaagatctataattagaggtatggttcttggagccatatctaatgttgttgttttgca
DdCoV_1a   tacaaagtctattattagaggtatggttcttggagccatttccaatgttgttgtattgca
           *****..***** ************************** **.*********** *****

PdCoV_1a   atctaagggacatgaaaccgaagatgttgaagcagtaggtatattatccctttgtgcttt
DdCoV_1a   atctaaaggacatgaaactgaagagcttgagtctgttggtatcttgtcactttgtgcttt
           ******.***********.*****  ****. * ** ***** **.** ***********

PdCoV_1a   ttctgtagatccagctgacacgtatttaaagtatgtggctgcaggtaatcaaccattggg
DdCoV_1a   tgctgtgaatcctgaagagacgtatttgaaatatgtgtctgcaggaaatcaaccacttgg
           * ****..**** *  ** ********.**.****** ******* *********.* **

PdCoV_1a   taattgtgtaaaaatgttaactgtacataatggtactggttttgctataactactaaacc
DdCoV_1a   aaactgtgttaagatgttgactgtgcataatggtaatggttttgccataactactaaacc
            **.***** **.*****.*****.********** *********.**************

PdCoV_1a   cagtcctactagtgaccaagattcatatggtggtgcatcagtatgtctttattgtagagc
DdCoV_1a   cagtccgacacctgaccaagattcttatggtggtgcttctgtgtgtttgtattgtagagc
           ****** **   ************ *********** ** **.***.* ***********

PdCoV_1a   acatgttgcacaccctggtagttcaggtacattagacggacgttgtccatttaaaggttc
DdCoV_1a   acatatagcacacccaggagcttctggtactttggatggtcgttgtccttttaaaggttc
           ****.* ******** ** . *** ***** **.**.** ******** ***********

PdCoV_1a   ttttgtacagatacctgttacagaaaaagaccctgtaggtttttgtcttagaaataagat
DdCoV_1a   ttttgttcaaatacctacttctgaaaaagaccctgttggtttttgtctacgaaataaagt
           ****** **.******..* * ************** ***********  *******..*
```

```
PdCoV_1a   ttgtaaagtttgtcaatgttgggtagggtttggttgccagtgtgatgcacttagacagcc
DdCoV_1a   ttgtaaagtctgtcagtgttgggtaggttttggctgtcagtgtgattcacttagacaacc
           *********.*****.*********** *****.**.********* **********.**

PdCoV_1a   tagtatgcaagttgcatatgaagaacctaacgttagtaccaatgttgacaaaaattattt
DdCoV_1a   aaaaccctcagttcaatcaga------tgctggtgcacctggttttgataagaattattt
            *.   . . ****  **  **      *.  .* *.    *...* ****.**.********

PdCoV_1a   aaacgggtacggggtagcagtgcggctcgactga
DdCoV_1a   aaacgggtacggggtagcagtgaggctcggctga
           ********************** ******.****

PdCoV_S   atgttggcaatgtttgtgttgcagat
DdCoV_S   atgttggcaacgttagttttgttgac
           ***********.*** ** ***.  **
```

3.2 Alignment of the 1s gene between a PdCoV and a DdCoV

```
PdCoV_S   gacagttatgtgtgttgtga---------------ttagtgttgtttgt---------tg
DdCoV_S   gacagttttgtgtgttgctaatccatgtttaacttttaatggtacttctcatggtgtaca
           ******* *********.  *               ***.** *..** *         ..

PdCoV_S   tcaaaacaacctaaccaca---------------------gattattatgatacatcttt
DdCoV_S   tgatggtaatttcactgcacgtgtttgtgatgggcctgctggttattattcttcatcacc
           * * ...**..* **..**                    *.*******  * **** ..

PdCoV_S   agatagaaggcctgatggtgtttataaagatgtgggtgtttttaaagttgtcaaagaagg
DdCoV_S   aattcgtccatctgacggtgtctatagtgttaacggtttttataaacccgtttatacatg
           *.  * *   ..****.*****.****.  * *.   *** *** **** ..**.  * .  * *

PdCoV_S   ttgtatggttagttgtaattgtgtaggcggctctggcactggtgttaggtcaggtcgttt
DdCoV_S   ttgtat---------------------------------------------aaggcatac
           ******                                              *.* *.* .

PdCoV_S   taattttacaaataagccacaat---------------cttttcttatgtatgagctggg
DdCoV_S   ctatccgaaaaatacgacgcagtgtgcgaataaaaagtttctgttttgtatta------
           .  **..  * ***** * *.**.*                .**.* ** ***** *
```

```
PdCoV_S    gtgctatggacctaacagtcagcctgttcagttacaagtcataacacctctaggtaccat
DdCoV_S    -tattaacgacacaacag-aggctttttccattaccatacccagagcc----gttgttat
             *. .**  *** .*****  .**.* *** .**** *  * .*.  .**    * *...**

PdCoV_S    taaatttaatagaaatggtagtggtaccaaccctaccagtaaaattgatgttacgtcacc
DdCoV_S    taaaccggacacaga-------------------atctttcattttgtcgctgggtgatt
            ****.. .*.* *.*                   *.*  * *  *** .*.*. ** *..

PdCoV_S    ttttataac-----------------------gctactggtatacctccagtaccgttaa
DdCoV_S    ttttactcacagctaaacagctgttagtttatggcactattcagtctcttatgtttttgc
            ****  * **                        * .***. *  ..***. .*... **.

PdCoV_S    aaggaggttgtatgtatggttatagagttgtcactaaacctcttatgatttttgttgaca
DdCoV_S    gtaatggtacttggtatattaacgg---------------tgttccggctcttcctgtca
            . .. ***  *  ****. * *..*               * ** .*..*.** .** **

PdCoV_S    acacaattctttattcttctttagaagaaacatatatta---ctaatgtaacttttagtg
DdCoV_S    gttacacctttaataccactttaaatgtcactcatactagctgttatgagagtattggtg
            ..    *...** ** *. *****.* *  ** .***.**    * *** .* * **.***

PdCoV_S    ttccagcaggttgggattgttttagatatgctgctagtgtttattcagaagcaatggcag
DdCoV_S    cacaaac-------------ttttattttacttcactaatcctttctaatgggttagttg
            . * *.*             **** . * *.** *    .*.. *** .* * . *.*. *

PdCoV_S    aatatagaaatggtgcagttataaacgctacagcctgtgacatcaatgatttcactaggc
DdCoV_S    aattttctgctggcaacctttcttcggtctgttgcttgtgaagacaacactattataaact
            *** *   . ***..    ** *  .   **.. **.***** . ***.. * *.*. *. .

PdCoV_S    ---tgcagtgttctctcatgcaatttaatatttcacaggggatgtatccgt---------
DdCoV_S    ctatgcaatgtagtcaccagcgatttaatttttcgactggtttgcattcttatgacagtg
               ****.***  ** *  **.******* ****.    **  **.**.* *

PdCoV_S    ------taacaccccagaatatgatatatgctc---------------------------
DdCoV_S    ttgttcctgtttctggtaatgtcacctacattccttaccctggtgtcggggacaattcga
                  . .. .*. . ***.* *. **...**

PdCoV_S    ---------------atgtagatgttttgccatatgttcaaggtaataaaagcaatggcc
DdCoV_S    gtttggagttgtacagtcttaatgtttcgcttcgttctaaaggtaactatggcgttcatt
                           .* * .******.**. ..* .* *******. * .**. * ...
```

```
PdCoV_S    aaacaatccagtatattaacctagcacctaatatgtcatattctgttactggaccgttcc
DdCoV_S    ataactatacttgtgttaatgcttctctgtacacctattttcgtgtttattgtcaggatg
            * *      .    *.*.****. .   * *.  *.*. *  * *. ****  * * * *  .

PdCoV_S    agtttaatt----------------------------------ttacaggtgtaggag--
DdCoV_S    aacatgattggaatacagaaaagctatgtactgtgtctgactattatgtgcctggtagac
            *..  *.***                                ***..  *.  *.* **

PdCoV_S    ---------------gatttcagtgttctagttttaaatgggccaatggtcaggatttta
DdCoV_S    atttgtatggcagccaacatcaatatgttggcattgtccctcattatactacctgttctt
                           .*.  ***.*.* .*.*.  **.   .    . **.  *    .**.*

PdCoV_S    caatctatcccagttggtctagcttgaaagttggtactggttttgattattttaacatta
DdCoV_S    cgttagggcttagccttagtaatattaataatttgggttttgatactttttgtatta
            *.  *   .  *..**..     **..  * ** .  *..* .  *********  **** ..****

PdCoV_S    ccacttctg--------ttaaatataccacaggtgtacaggatcctgtagatggttgtca
DdCoV_S    ctaatgctaggtctacttataatatagcagaagtcacccagtttcaatgtctttttgtga
            *.* * **.        *  ****** ** *.**   * .* *.* .*.   *  **** *

PdCoV_S    agcttggtttaggttttgtacctgtaacgaatggggttatggaacctgtggttttactgc
DdCoV_S    ttgatgttatggtttttgatgctaca--------ggtggtagtagttggggtcaacctat
                ** * *.* *****   **..*        *** .*.* * .** ***.    **..

PdCoV_S    taggccagggcaaaggtactatgtttgtgttaattataagtttggcaataagttttatta
DdCoV_S    tg---------aatatttttatgttggcctcgatttttcttttggtaatagaatgtat--
            *.         ** .  * .****** *.  *..*** *   *****.****..  * ***

PdCoV_S    tttaaataagctagcaactaaaggtggttctaaaggtaacaatgttaccatagaactaaa
DdCoV_S    ----------------------ggtgtcttgcaaattcctccgcaagttatatatgaaga
                                  **** .*.   **.  *   .        ...*** *   *.*

PdCoV_S    caagtgctattcttattctatttatggttacaatggcactggaatttttacaaatgctag
DdCoV_S    ccagtgtcattcttatgatatttatggtattaaaggcactgg---tcacatctataatgt
            * ****..********  **********  .** ********   *.  .*.   **.  *.

PdCoV_S    tgctgatgtttatagacagaacctaggtggcggcagattagtgcagtcagttagtggtgg
DdCoV_S    cactggttatgacaattacaccttatctacaggtggtttggctatatcaggtggtaatgg
            ..***.*   * *.*.  .* * *.**   *.    **..* **.*.     .**** *.**..***
```

```
PdCoV_S    tgttacatatgccagagttaatataacaggatatggtgtgcagatttataaagtaacacc
DdCoV_S    tctattggcctttcgtaataatggttctttgtatactgtcaaaccgtgttctacagtatc
            * *  ..   . ..  * .  ****.    *    .***.  ***   *.  .  *.*    ..*..*.*

PdCoV_S    ctgtaaaacttttactactaatgtgttgacttttggcagaaaggtagatggttatttctt
DdCoV_S    tactcaggctgttat--------tgtagctaaccagttggcagggttgtatttaccgctc
            .   * *..** ***.         *** * .   ...*.  *.  ***    .*.  ***..  **.

PdCoV_S    tcgtg---------------gtgttaatataacagaaaaaccttttatacaaattgtaca
DdCoV_S    tcatgtgacatagtttttaatcttaatcttggtaatgaaactactcctgttgatggcggc
            **.**                  .* ***** * .   .* .****.  .*.  *..  .** *..

PdCoV_S    gtatatgaatctggtacagtttgtactaatggtacctctagagctcgtagtaagaggtca
DdCoV_S    tgtttagttttaattcaacttttaagaaccgacaatcacagctttatggcagtgagtgt
                *   *   *.*..* **..** **   **.  *       **   ..  .*..*.*.*.  ..**

PdCoV_S    attggttcacttactgtgcaggattgttctaatggtattaccttaggtaataacctttgt
DdCoV_S    tatggtgtatttgccactattggttcttc-------------------------------
              **** .*.**.*...      *.** ***

PdCoV_S    tttacacctaataaacaggttttgactattaagaagtttactacaaactacactgaatat
DdCoV_S    ----------gtgtatttatgctaattcttctgtgttaaatcgtacactgccgagggcag
                      .*.  *.   .* .*.*.* **   * .  *   *....* ***.*    *...*

PdCoV_S    gcacctcttgtgttttcagatgcagctgtagaa---------------------------
DdCoV_S    gcg-------------tagataccactgtagaaccattattggatgtgacggccaatgtt
            **.                .****.* .********

PdCoV_S    ---atacctcttacacttaaaatggctacaacttctgaatttatagcaacagaagctgaa
DdCoV_S    tccataccctataccctgacactggcggtcactactgaatacttacaaacatcttaccag
                *****..  *** ** * * ****  ..  *** ****** .  **   ****       .  *.

PdCoV_S    agagtcagtgttaattgtgaacaatttgtttgttcaggttcacaaaattgtcttaacttg
DdCoV_S    aaagtcactattgactgtgcccggtatgtgtgtggtgagtctcttaaatgtcgtactctt
            *.***** *.**.*.****   *..* *** ***    *.  ** *   ** **** ** ..*

PdCoV_S    ttggcacaatatggtagtgcctgcactaatattataaaatcacttagtgatatgaatgat
DdCoV_S    ttgcagcagtatggcagtttttgtaccagcgttaataatatattagctggtgttaacgat
            ***   .**.*****.*** ..**.**.*...***   **   .*.* .   **.*.* **.***
```

```
PdCoV_S     -------------------------------------ttagaggcggttgatgtttcagc
DdCoV_S     aatgaggataacggcatgctttcttttgtggaggctattaatactggttattctcttaac
                                                  ***. . .****. * *.*.*.*

PdCoV_S     ttttatgcacagctacctcaaa-----ataatgttaatttatcttcagtagtttctcaa
DdCoV_S     ttttctagttttaacagttcaaaccttggtgggtttaatctgtcgttagtctt-------
             ****.  *. . . .* .*****    .*..  *****.*.** *.***  *

PdCoV_S     gagagtgatttgcatttgagcagcataatgccacgaaccactcaagggaggtcaactatt
DdCoV_S     ----------------------acctaattcagcaaagccctc-gggacgttcctttatt
                                  .* **** * .*.** * *** .**.  * **  .****

PdCoV_S     gaagatttattgttttctaaagttacaactgttggtttaccaacagatgatgcttatcaa
DdCoV_S     gaagatttactctttgataaagtggttactgtcggtgtgggtgaagttgatgctaattat
             *********.* ***  ****** ..  *****.*** *.   . ** ******* **.*

PdCoV_S     aagtgtttagatggacaggaaatacgtgatattttc-------------------tgtgc
DdCoV_S     ga-taagtgcatggattcaagaggcggcaagtttactaatgctgctgacttgacatgtgc
             .* *.  *. *****.  .*.* .**  * .*** *                  *****

PdCoV_S     tcagtattataatggaattatggtgttaccacctgttatcactccaggcttgcaagctgg
DdCoV_S     tcagttttataatggtatcatggtgcttcctggagtggtggacccagaccttatgacact
             ***** ********* **.******.* **    ** .* . .****.*.*   ..*

PdCoV_S     ctacaccgctggtcttataggagccatgacttttggtggtataacttctgctgccgctgt
DdCoV_S     ttatacaggctcacttctgggtggcatgtcttttggtggtctttcttctgctgcctctat
             .**.** * .    *** *.** * **** *********** *  *********** **.*

PdCoV_S     accttttgcaacccagattcaggcgcgtattaaccatcttggtattacgcagaccatctt
DdCoV_S     accttttgccacacaggtgcaggcccgtattaattatttggcacttactcagtctgtgct
             ********* ** ***.* ***** ********..**.* *   **** *** *..* .*

PdCoV_S     gctggaaaaccaaaaggagatagctaacaaatttaatgctgctttaaactata-------
DdCoV_S     tttggataatcaaaaccttattgctaattcatttaataatgctcttgaaaaaatacagtc
              .**** **.*****    ** *****.  *******.  ****.* .*  * *

PdCoV_S     -----------------------------------ctatacagggttttcaagcaattga
DdCoV_S     agcacttgatgtggtgtcagctggttttcaagaggttgcaaaaggctttgagactgtttc
                                                .*..* *.**.*** *..* .**
```

```
PdCoV_S   tagtgctcttactaaggttgagagctttgttaatcaacaatcttatgtcgttaatgaggc
DdCoV_S   tgtagcactttctaaggttcaggatgttgttaattcacattctgagattttgaataaact
           *.   ** *** ******** **...  ********.  *** *** * .*.  * ***.*.  .

PdCoV_S   ttttaaagctataggctccaattttggtgctatttctaatgccattaacgacatttatga
DdCoV_S   tatggcgcagcttaatgttaattttggcgccatctcttcttctttgtctgatatctattc
           * * .  .     * ...  ..********.**.**.***   * *.  *    .**.**.***

PdCoV_S   tagacttgaccaagtgactgctaatcaacaaatagaccgtattattactggtcggttagc
DdCoV_S   tcaacttgacgagcttaatgctaacgctcaagttgatagattgataacaggtaggttgac
           * .******* *.  * * ******.     ***.* **.  *   * ** ** *** ****..*

PdCoV_S   agcacttgtgtcacttgtatcggcaaaacagttgcaactgttagaagttaagcagaatcg
DdCoV_S   agcactctctacatatgcagcttctttacagttggctgcatataaagcagaagagtcgcg
           ******.  .   **.  **.* *  *    *******     ..*  .***.  .*.  **    **

PdCoV_S   cattagggctgctgaaaaagtggctgaatgtgttatgtctcaaagcgaccgttatggctt
DdCoV_S   taaattggctttgcagaaagttgaagaatgtgttaaatcacagtctatgcgttatgggtt
           .*     **** .   *.***** *  ********** .** **.    ..   ******** **

PdCoV_S   ttgcggcaatggttcacatattatgaccataccacaagcagcacctaacggtatgctttt
DdCoV_S   ttgtggtaatggcagtcatgttttaaccataccacaaacagcacctaatggtatgttttt
           ***.**.*****.     ***.** *.***********.**********.******.****

PdCoV_S   tatacatgtgtcttatatacctgacacatatgtaacagttaatgccacacctggtttctg
DdCoV_S   tatacactatacctatccccctgcctcatatataactgttgaggcggtaccagggttgtg
           ******.     *.*** .  **** * *****.**** ***.* ** ..*** ** ** **

PdCoV_S   tgt---------taatagcagttatgctttagcacctagaactggtgctggcatattctt
DdCoV_S   tgttaccacacgtactggttcttatggtgttatgcctaaaactggtagtggtattatatt
           ***          ** *.*.   ***** * * ...****.*******.  ***.**   * **

PdCoV_S   taaaaattctgattttccagattcttatattaatggctttgccattactagtagaacact
DdCoV_S   taggg---------------------ctaacggcacgttctttgtaactgcccgtactct
           **...                          ** .....   **.  ...* ***.  .  * ** **

PdCoV_S   ctatcaaccacgtgcaattgaactggctgatatggtgaatcttactacttgcaatgtcac
DdCoV_S   ttatcagcccaaattgctttcttattcagacgtcgttaatttaacgtcgtgtgaagctaa
           .*****.**   .   ..  **     .    * **..* ** ***.* **   * **..* *..*
```

```
PdCoV_S   ttttataaatgtcacttatgacattatcaaagtgccagtgtttccagattt---taattt
DdCoV_S   ttattataatgttagccaaga------tgaaacaccgtttcagcctgagctacctagttt
          ** *   *****.* ..* **      ..**...**. * .  ** ** .*   **.***

PdCoV_S   tactgaagattttgataaatggtggaatgaaacccgccct---------------gaatt
DdCoV_S   tgatgatgagttcgataatatatataccgaactcaacacttctaaagacctcatagactc
          *.  *** ** **.*****   .*.  * .*** .* .* **              ** *.

PdCoV_S   tcctaatatagatttcaatttcacagtacctttccttaatat------------------
DdCoV_S   aatctataaggattttaattataccatccctattcttaatttagaggatgatatatgtag
            ..  *** .*****.**** .** .* *** *.****** *

PdCoV_S   ------------tactagtgaacttgaagatcttagaggagtagtagacaa---------
DdCoV_S   gttgaactcttctattaatgcgctgtggaattacagctctattatagatgagatcaatgc
                      **.**.** .**  ...**. .**    .* .****..*

PdCoV_S   ---cctcaaccagtcttatatagacttgaaagatctgggtgtcttaactacttatataaa
DdCoV_S   tgcgctcaatgacacttacattaacctggaacaacttaataaggttaccaggtacattaa
              *****. *  ****.** .**.**.** * ** ..*.   * **.*  **.** **

PdCoV_S   atggccttggtatgtttggttggccattggttttgctgtaattatttttattcttatttt
DdCoV_S   gtggccttggtacgtttggcttgctataggttttgcatgccttatttttgtgttaatcct
          .***********.******.* **.** ********     ********.* .* **..*

PdCoV_S   aggttgggtctttttcatgactggctgctgtggcggttgctgtggctgctttggaataat
DdCoV_S   atgctggattttctttatgactggttgttgcggatgttgctgtggttgttttggtataat
          * *.***.*.**.**.********.**.**.**  **********.**.***** *****

PdCoV_S   cccacttatgtcacgttgtaataagaagtcatcttattacaccacttttgatgatgatgt
DdCoV_S   acctcttatgtacaagtgcagcaaaaaatcttcgtattacaccacctttgatgatgatgt
           ** *******   .  **.*..**.**.** ** ***********.**************

PdCoV_S   tgtaggcgaacaaatcagacctaaaaagtccgtttga
DdCoV_S   cgtaggtgaacaaatcagacctaaaaagtttgtttaa
          .*****.*********************..****.*
```

3.3 Alignment of the 1a gene of a PdCoV and a dolphin CoV

```
PdCoV_1a   ATGGCAAATAGCCAGAAGAAGGGAGTATCTCCCAATCAAAAGAATGTCATCTTTGTAGTG
DolpV_1a   ATGTCTAAGTACCAAAAGTCAGTAGTTTCTACT------AGAAATGCAAAGGTGGAGGTG
           *** * **  .***.***  .* *** *** *.      *..****. *   * * .***
```

```
PdCoV_1a    AAGGGCATTCCACCTGCCTTGTGTGACGCGTTGTTCTTTTATACGTCACACATTCCAAGC
DolpV_1a    GTTTACACCAGACCAGGCTT---------GCCGTTCCTGGATTACCTGCGCATCATGTAT
            .   .**..  *** * ***         *..****.*  **   ...*.***. .. ..

PdCoV_1a    GACTTTGCTGATGCATTTGCTTTTCGTGAGCAATTTGATCGCAATTTGCAGAAAGGTCGC
DolpV_1a    GGACCAACTAAGAAGTTGGAT---------------------------CAGATAAAGGAC
            *. .. .**.* . .** * *                           **** *..  .*

PdCoV_1a    GCTTTCAAGTTTGAGACTGTTTGTGGCCCTGTCATGTTACAGGGTGTACCAACAGTACCA
DolpV_1a    ACCTCCATTGGAGAAGTTCATTGTGG--GGATAAGGTCCTTAAATTTATCAAGAAC----
            .*.*.**     **...*  ******    .* * **. ...* **.*** *..

PdCoV_1a    CCTGGTGTGAAAGCTCTTGATGCTAATTCTAAGCTTGCTGATTTAGAGC-----------
DolpV_1a    CCCGTAGTGGGTATTGTTTGTAATGAAGGTGATGTACCCTTTTCAGAACTTGAAGAAAAG
            **.*  ***.. ..* ** .*. *.*   *.*  *  *.  **.***.*

PdCoV_1a    ------------AAGTTTTTGGTGTGTCACCACTGTCACGCCGTTATAGAGAACTCCT--
DolpV_1a    TATGGAGGGAATGAGCTTACTAAGCTCTATCAGCACCTTGCTGCTACTCAGTCTATTTGG
                        .**.** . . *. ..*.** ...* .**.*.**.  **  .  ..*

PdCoV_1a    --TAAAACCGCTGCCCAGTGGTCCCTTAATGTTGAGTCT----------CTTGACGAGCA
DolpV_1a    AGCAAAAGCTCTGCCGATTTGGTGGCAAAGGCTGAGTCTGTGGCGCATGTTGAAGGAATT
              .**** * ***** * * * .  . ** *.*******          .* .* **..

PdCoV_1a    GGCAAGTAAGTTGGACACTATTTTTAATTCTTCTGAAATTTTGTGGCTACAGGTTGCTGC
DolpV_1a    GATAGATTGGTTGGATGTTACAACC----------------------------TTCGTAT
            *..*..* .******...**.  ..                            **  *..

PdCoV_1a    CAAAGTACAAGTGTCTGCAATGGCACTTAGA---------CTTATGGCAAGTGAAGTAGT
DolpV_1a    CAATATGTGTGCAACTCTTGCAGTGCTTAAGAACTTCCTTTTTAAGATTGAAGGTCTTGT
            *** .*... *.. ** . ...*..****..         .*** *.. .. *.  * **

PdCoV_1a    CAAGAAAATTGTTAAGACACTTGGTAATGAATTTTCTACCATCTTTTCTGTTGTTGTTAA
DolpV_1a    TAAGAATGTAGCTGAGAGCTATATTGAACCTATGCAGGGTGAGTTTGATGCTTTGCTTGG
            .***** .* *.*.***  . *. *.*     * .  . ..  ***  **.* *  **..

PdCoV_1a    TCAGGTGAAGAAGATCATTGACAAGTGTCTCACTGTTTTTGAGAATGTTGAAGACCTACC
DolpV_1a    TAATTACCTAGCGAGTGTTAAAATTACACCTACTGTTGTTAAGGAGTTGGCAAAATTGGC
            * *      .. ** ..**.* *     *..****** **.**.*  * * *.* .*. *
```

```
PdCoV_1a    ACAACGCATAGCTGCCCTAAAGGCTGCCTTTGCAGCATGTGTGAGGCGCATGACTATTGT
DolpV_1a    CAAGTCCATTCCTATGCTGAAAG------TTGAGAACCATGTGCTGCGTTTGTTCGGAGC
              *..  ***  **..  **.**.*      *** ..  ..****  ***. ** ...   *.

PdCoV_1a    GATTGTGGATAAGGCTCTTTGTATTAAAGAGTTAGCAGGTACTTCTCTTGCTAGTGTGAA
DolpV_1a    GGGTGGTGATTTTGTTGATTGTATCAACAAGTT------------CCTTTCAGATGTGTA
            *. **  ***   *.*  ******.** .****            .*** * ..**** *

PdCoV_1a    T--------------GCAGCTCTTGCAACATGGTGCAAGACTCTGCCCACTGGTTTTATG
DolpV_1a    TTATAAACATCCTGGGCATGTGGAATATCATGGTGTGAAGGTTTCCGCCACGATTTTCTA
            *              ***  *   ..* *******..*..  *.* * *  .*.**** *.

PdCoV_1a    ----------GGTGCTAAGATTTTTGACAACTTGGCTTTTTATAAAGAAGCGGTTGTCAA
DolpV_1a    TCGCAACAACCGTGCAAAGATAAGCCGTGCGTATATCATGCCACCAAAAGTTGTTTGCAA
                       **** *****   . ...  *  ... * .    *.***. ***  ***

PdCoV_1a    ACTTGTTAGCAATGTTGCAAATGCACCTAGTGGTGTTTCAGGTTATGTGGTGATTGGTAA
DolpV_1a    GCCCGATGTGTATTTTGAAGCTACACCTGCTACTCAGTT---TCAAACTGTGATTGTTGA
            .*..* *.   ** *** *. *.*****. *. *   *.   *.* ..  ******* *.*

PdCoV_1a    GGCGCGTTGTACACAAGTTGTTGTACGCGGTATGCGCTCAGACCTCACCTTGTTAGATCA
DolpV_1a    AGGAAGTGGAGAGGAAGT-----------GCATGAGCTCCGCCAAGTTATTGGTGAA---
            .* .  ** * .  .  ****           *.*** **** * *    . *** *..*

PdCoV_1a    AAAAGCCAATGTTCCCGTTGAAAAAGAGGGATGGAGTGCTGTTTTTGATGGTA--AACTA
DolpV_1a    ----------GTACCAGAAGGACAGAAAGGCATTATTGTGCTTTCTGATGGTGCTGCCTA
                      ** ** *  *.* *..*.**    * **.  ***.*******.   .  ***

PdCoV_1a    AGTTACGTGTTTAAGAGTGGTGATTGTTATTATGCAGCACCACTTGCAGGTAACGTAGCG
DolpV_1a    TTTTAAGTACCTAGG---------------------------------------------
              *** **...**.*

PdCoV_1a    CTCAGTGATGTTTATTGTTGTGAACGCGTAGTTTACCTTGCAGATGGTTTT---ACACCT
DolpV_1a    ------------TTCAGATGGAAAGGAATACCTTTCACTCTGGGTAGGTGTGGGATGCCT
                        * .  * ** .** * .** .** * .* ..*.*.* * *   *..***

PdCoV_1a    GAAATA----AATGATGGATTGTTGCTTGCTGCATTGTATACATCTAATGTAGCTGTTGA
DolpV_1a    TAAATACCCTATTTATGTGTCAGAGCAACCTGCAGCAGTTAAATTTGCTGAGGCTGTGTC
             *****    * * *** .*..  **   ***** ..  ** **.*. ** .*****
```

```
PdCoV_1a   TGTTTTGGCTGCATTGCGCAAAGGAGAGCCATTTAAGTTTTTGGGCCACTCTTTTGTGTA
DolpV_1a   AGTGTTGGAATATGAACAAGAAGAAGAGCCGGACATTGCGATTAGATGCAAGTTCAGATG
            ** ****       .*. .***.******.  .*   .  * .* ..*   **.. .*.

PdCoV_1a   TGTTAAGAATGCAGCTGTGTCTTTTACTTTGGCAAAAGGTGCCACTGTAGGTGATGTTCT
DolpV_1a   TTTTAATGGC---------------------------AACCACATTGAAAAAGACCTTGA
           * **** ...                           ...  **.** *.. **. **

PdCoV_1a   TAAACTTTTTGTTAAAGAAGCATCTGATGTTTGGTCTACTTTTGATGAGAAGTCTCATGA
DolpV_1a   TAAGGCAATTGATGAAATTGTATTTGAAACAGAGTGTACCATTGAGGAAGTTCATGCAGT
           ***. .  *** *.**.  *.**.*** ..  .** ***. **** **..  . *   *

PdCoV_1a   ATTTTGGACTAGAGCTTACAATAAGTGTCGTGACTTGACAGAACTTGTCAAAACACACTA
DolpV_1a   GTTTTATACCAGAATGTGTGA---------------------------------------
           .****. **.***.. *...*

PdCoV_1a   TTGTAAAGCTCAAGTGACTTTTGTTGTACTTGCTGTTGTTTTGGGCTCTACTACATGGGA
DolpV_1a   ---------------------------------------------------------ATA
                                                                    . *

PdCoV_1a   GCTTTGTAAGCAAGTTATGTTTAAAGTGGCAGGTCTTTTT------TCTGCAGTTGTAGA
DolpV_1a   TTTTGAAGAGCAAGGTAGCTCTGAAGAAATTCCACTTTTTGAAGAAACTGCCCTTGTGGA
            .** . .****** **  *.*.*** ...    ******       ****  ****.**

PdCoV_1a   TTTTTGTAAGAGAGCTTGGGATGGGTTTTGTCAAAAACTAGCCCATGTGAAGCTTGTAAT
DolpV_1a   TCCTGATTACCGTCTTTTGGATGGTCTC------AGAAAGGTTGTTAGATTAGTCATGTT
           *..* .* *  *  .** ****** .*.      *.*  .*..  *. .  . *..*. *

PdCoV_1a   TAATGAAGTTGGCTGTGTGTTGCATGGAATTAAAGAACATTGTTTCCAACTTCTTTTTGA
DolpV_1a   GGGTGAAGTCGCCGATGCGGAGGACGAAGACAACTATGATTG---------------GAA
            ..******.* * .**.*  * *.*.*. .**  *  ****                .*

PdCoV_1a   TGCCTTACACGCACTTTACAAGACTTTTGACAAGTGCGCCATAGGTAGAATTTTTGCAGG
DolpV_1a   TGATGAAGATGCAGAGTCCCAGCCACCTGATG---------------------TTGAATC
           ** .  * *.***   * * ** * ..***..                     *** *

PdCoV_1a   TAACCTGCTGTTTTGGGCAGGAGGTG---TTCACAAAGTAGATGAGGACAACTCTGAAGT
DolpV_1a   TGAAGAGCAGGTTCAAGATGAGGATGACTCTGTTGAAGTAGATGAAGGTGATTATGAAG-
           *.*   ** * **...*  *..*.**   .*  ..**********.*...*.* *****
```

```
PdCoV_1a    TTGGTTTGATGCCATTGATACTGTACAAGTTCAGGATGAAGGTACAATTGTCGAAAACAA
DolpV_1a    -----AAGACACCTTTAAC------CTAGCTATGGGTGTACCTATGATCCATAGCGATGA
                   **..** **.*.      * **.*  **.** *  **..**.  ... .*..*

PdCoV_1a    TGTGCCCGACTTTGTAGCCTGCCCTGATGTTTCTATACCAG-----AGCGTCAGAAAGGC
DolpV_1a    TGAGCTTGACCTTGTATGTGATGTTGATGATGATGAGCTGGTTGACGAGGGCGAAGAGCC
            ** **..***.*****  . .. .***** *  *. .*..*     .. * *..*.** *

PdCoV_1a    TATCTTGTGCAGATTATGGCTGATGGTAAAACTTATATGTTTTTTAGATTTCAGCAAGAT
DolpV_1a    TGATGTGCGGAGTTCATCA-----GAAGAGACTGACCTTGAGGTTAATGTTCAGTGTAAT
            *. . **.* ** *.** .     *. .*.*** *. *     ***.  *****.. .**

PdCoV_1a    TCAAACACATACTATGCACCTATGTCCCAAGTGGGTGCTTTGAATGTTGTTTGTAGAGCT
DolpV_1a    GATGATGCAGGTTGGTCTGAGGATCCTCTGGTGGTTGATGAGGATTCAGCAGACACCTCT
               .*..** ..*.  *    .  .*.* .**** ** *  *.** . *.  ..*   **

PdCoV_1a    GGTGGTAAGACAGTAACATTTGGAGATGAGGAAGTAACAGAACTCCCACCACCAGATGTG
DolpV_1a    GCTCAAGAAGAAGATGAAAACGAAAGTGCTAGTGCGCCTGAAACCTCTCAAGAGGATATG
            * * . .*.. **  . *  .*.*..**  .. *.. * *** .*.* * *  .***.**

PdCoV_1a    AAGATGATATATATAGACATTGAGTGTTGTGGAGAACCGTGGACTAGTATCTTCAAGAGA
DolpV_1a    GAAACTACAC---------------------------------CTGAGACATCTCATAAT
            .*.*. *.*.                                 **.. *. *.. * *.

PdCoV_1a    GTTTATAAAGACCCTGTTGAAGTAGAGACTAATCTCACTGTTGAAGAATTGAAATCTTAT
DolpV_1a    AAAAACGAAGTTGCTAGTGCAATGGAAACCACATATATTACCAATGACATGGCGGTGTGT
            .   *..*** . **. ** *.*.**.***  . .*.*....* **  **. . . *.*

PdCoV_1a    GTCTATGGTATGATGTGTGACAACCTAAAATTGTTGCCAGAAGCACCACAACCACCGGCA
DolpV_1a    A------------------------------------------------ATCCAGAAGCC
            .                                                * ***  .**

PdCoV_1a    TATGAAAATGTTTCAATTGTTGACAAGAAAGGTCAACTTCTTAAAGTTGTAGATTCTTGT
DolpV_1a    TTTGTTGATGGAGATATAGTGGAAATTGAAAATCAATTTTCTAGTGTTAT----------
            * **  .***     ** ** ** *  .**..****.**..**. ***.*

PdCoV_1a    CGTCTCGTCTATGACGAGTATGATAGTGATGGTGCTGTGTCAGATGATGACTATTGTTCA
DolpV_1a    --------------TGAGGAGGGCGAGAAAAACGCTACCTCAGCTAGTGCAGATATTGTA
                          .*** * *.... .* ...***.. **** *..**   **  * .*
```

```
PdCoV_1a    GACTCTGATGTGGCAGAAGATGATGATGAAGATGTGCAGGAAGTGGATAATGATGATGAT
DolpV_1a    AAATCTGCGGTAG-----------------------------------------------
            .* ****  **.*

PdCoV_1a    AGGCTCGCAGGACTTTTGAAGAATCCTGTGAACTTTAAATATCCACTACCTTATGATGAT
DolpV_1a    -GTCTCGTTGACCCGCGGAGCGATTCCTTGACTGACCAGCAGTCG------------GGT
             * ****. *. *. . **. .**.*. *** .  . *..* .*.            *.*

PdCoV_1a    GATTATAGTGTCTTCTGTGGCCAATTGGTGCATAGGGACGCTAT----------------
DolpV_1a    GTTGGTGACCTTGTGTGTAAACAGGTTGTTAGTAGTGTGGCTACGGTGCAGAGCGAAGTG
            * * .*... *. * ***.. **. * **  .*** *  ****.

PdCoV_1a    --------TGATACTTATCACCACCCATCTGGTGATGACATGTATGTAGTCAACAATTCT
DolpV_1a    GTAGGCGAAGGCAGTCCCGTACATACGCCTGATGCTGACGCGCTCATTGCAA--------
                     *..* *. .   **. *..***.** ****..*. ..* *. *

PdCoV_1a    GTTGAAGGTGCTGTCAAAGCAATTCCTCAGAAGGTTTTTGACTTAATGGATGATTGGGGT
DolpV_1a    ----------------------------------------GCTCCACTGCTAACCAAGGA
                                                    .**. *. * *.*....**

PdCoV_1a    GCAGCTGTGGATGAGCAAGAGAGAAGACTCAACGCAGAAGAGCCTGAAGTAGTAGTTGTA
DolpV_1a    GTAGTGACCAAAGGATTCGAGAGTGCTTCAAATGATGAAGAGTCTGTTAGTGGGGCTGAG
            *.**. .. .* *...  ***** .  .. **.*  ******.***  .  * .*.** .

PdCoV_1a    AGAGATGTTGCTATACTGGATGTGAACAAGGGTTCAGAGAATGTATTTCAAAAACTCAAC
DolpV_1a    AGTGTTTTACCCCTTACTGATTGTGATACTAGTTCATCTAGTA-----------------
            ** * * *  *. *  . ***   .*.*  .*****   *.*.

PdCoV_1a    ACCACTCTACCTGAAAACAGTGAGAGTGCTGTCAGTAATGAAAATGCAGAAGTGGTTTTG
DolpV_1a    ---ATTCTGCCAAACCTCTGCAACAGCGGCAAAAGAGGCTGAAAAAGAGAAAGGCT----
               *.***.** .*   * *..* **.* ..  ** ... .*** . ****. * *

PdCoV_1a    GAAGAGTCTACAGTTGAAGTAGACATTGATGAAAAAACCACACCTGTACAAACCACTATT
DolpV_1a    AAAAAATCTGGTGCTGATGCTGATACTGA-------------------------------
            .**.*.***.  *.*** *. **.*.***

PdCoV_1a    GAAAGTCATCCTGTTGACGTTGACATTGATGAAGCTACGTCTAGCATAGACGGTAGTATA
DolpV_1a    -----TCCCTCGAACTATTCTGGTATAAAGGGGGTTAGG---------GATGATTGTAGA
                 ** ..* . . *. .**..** .* *..*.** *         **.*.* *** *
```

```
PdCoV_1a    GACCAGTCTGCACAGGAAAACCTTGAGGATCAGAGTCCTGATGA------TAATCATGTG
DolpV_1a    GATGAATCAGCTCAGGCTTCGTGTAAGAGTGTATCCTCTGAGGAAGTGACAGATGTTGTT
            **.  *.** ** ****     . *.**..*  .  ..**** **       .**  ***

PdCoV_1a    CAAGCTGATGTAAGTAGTGAAAAAGAGGCTGTTATAGAAGAGTGTGCTGTAGAGCATACC
DolpV_1a    AAAGAAGATGAAGATTGGGGAAAAGCTGTTGATGCTCAGGAATGTA---------AAAAC
             ***  **** *..* * *.*****  *.** *..   *.**.***.         * * *

PdCoV_1a    GTTGAAGAACAACCTCAGGATTTTGTTTTAGTAGAGTCTAAGAAGAGGACTCCTAAATTT
DolpV_1a    CTGGCAGGGCAGACT-------------------------------AAACCCTTCATCTT
             * * **..**.  **                               ..**.*.* * .**

PdCoV_1a    AAATTGAAACCAACTAAGTGTAAACCACCGAAAAGTTTAGAGTTTTATACACATGTTGGT
DolpV_1a    TTATGGAGATTTAC-----------------------AGGAGTTGCTGAAGCAACTTGGC
              ** **.*..  **                        .***** .   * .**   ****.

PdCoV_1a    GAGCTTGATCTAGTCATTGCCAAGGCAATGGATTTGTTTGAAGACTTTGTCCTAGTTAAT
DolpV_1a    GGGTTTGGTTCCATAATC------------------------------------ATTAAT
            *.*.***.*.. .* **.                                    .*****

PdCoV_1a    GCTGCAAATGAGCATATGGCTCATGGTGGCGGTGTAGCTAAGGCTATAGCAGATTTTTGT
DolpV_1a    GCCGCTAATGAAAGATTGCAGCATGGAGGTGGTTTGCTAAAGTTGTAGCGAGTTTGGCA
            **.** *****.  .   **    ***** **.*** * *****.*.*.****...***

PdCoV_1a    GGACCTAAGTTTGTTGATTATTGTGA--------------------------TGCCTATG
DolpV_1a    GGCCAACTGTTTATTAAGAAGTGTGAAGGTATAATTCGCACAAAGGGACCTGTGCCAATG
            ** *    ****.**.*  * *****                          **** ***

PdCoV_1a    TGAAGAAACATGGCACACAACAACAACTCTTAGCTCCTTCTGGTGTAGCCAATATTCAGG
DolpV_1a    GATACACGTATGGTTCAAACTGGTCCTTATAA-----TTGTAGTATGTATGGAGTAAAGG
             . * * ..****. ** * ....  .* * *     ** *.**.*.   ... .*  ***

PdCoV_1a    CTGTTAACAATGTTGTGGGACCTAGACAAGGGCAAGACAACCTTTTTGACAAGTTGGTTG
DolpV_1a    CTATTAGAAATGCTGTTGCACCCAGAGGGAATGAACAGGATATTGAATCCAAGCTTAATA
            **.***. ****.*** * ***.*** ....  ** * .*.  **     ****.* . *.

PdCoV_1a    AAGCCTATGAAAGGGTTTTGGTCCCAAATGCAGTAAATTATGCCTTGCCTATCTTGTCA-
DolpV_1a    CAACTTATTACCATGCT---ATTCAGGATATTGTGAATAAGCCTGAGGTTATTATAACAC
             *.*.*** *  . *.*   .*.* ..**..  **.*** *  *.   * .***. *.  **
```

```
PdCoV_1a    -----------GTTGGCATCTTTGGTGTAGACTATAAACAGTCTATAGATGCTATGCGCA
DolpV_1a    CCTTGTTAGGGGCGGGGATATTTAATGTAGATCCTGAGTTGAGTCTTAAAGCACTCCTTA
                       *.  ** ** ***..******.. *.*.. *  * * .* **  * * .*

PdCoV_1a    AG----------------------------------------------------------
DolpV_1a    GGTATGCCACAAATAATGTGGTTATTATTACCAATGAGAAAAAGCAGTTTGAAATGCTTA
            .*

PdCoV_1a    -----------------------GCTCTTGAAGGGAAAAATGTTCGTGTGTTACTTTTTA
DolpV_1a    AAGCTCATGGTTTAATTGAAGCCACTTTTTCAGGCGTGAAAGCTTCTGCTCCAGGTACTT
                                   .**.**  *** . .** *.*. **. ..*  * .*

PdCoV_1a    CTTTGAAGCAGG-----------------------------------------------A
DolpV_1a    CGGTGAAGAGCGAAAAGGAGTTGGATTGGTCTGATAAAAGAACTGAAGAGGAAGAAGCTA
            *  ***** . *                                               *

PdCoV_1a    ACACATCGAATATTTCAATGCAAGTCTTAAGCAGA----------------AGACTGTTT
DolpV_1a    ATGTCCCAGGTACTTCGGTGAAAGACATAAGCACACCTAGAAGTGAGGCTCAAGTTGTTA
            *... .*...**.***..** *** * ****** *                *...****

PdCoV_1a    ATCTTACAGAGGATGGTGTTTCA-------------------------------------
DolpV_1a    CTCCTGAGGAGAAAATTAATGAAGAACCTAAGCCTGAAAAAGCTGAGGAGGTTAAGAATT
             **.*. .***.* . *. *  *

PdCoV_1a    -----------TATAAATCACTTGTCCTTAATCCTGGTGACACTTTTGGTCACATAGGTG
DolpV_1a    ATTGGAAAAGCGAGTGTACTCGTGAATTGAATCTTGGTG---CCGTCAGAGGATTAAATG
                        *  .  * * **  .* ****.*****   *. *..*  .  **..**

PdCoV_1a    GTGTTTTTGCGCGTAACAAAACTATCTTT-------------------------------
DolpV_1a    GCATCTATAAATGTAAAAAGAGTGTTTTTCATGGTAGTTGTAATAATCCAGAACATAAGG
            *..*.* *. ..**** **.* *.*.***

PdCoV_1a    ------------------------------------------------------------
DolpV_1a    GTTATTGTTGTGTATGTAATGGACATGTGTCGGAGGTAGTGTATTACTCTACCTTTGATG


PdCoV_1a    -----------------------------------------TCAGCTGACGATGTCGTTG
DolpV_1a    GTGTTAATTACAAAACCCATAAATTTAGTAATTTGGGTAAATTAGTGAAAGACGTCATAG
                                                     *.**. .* **.***.* *
```

```
PdCoV_1a    ATAAAGAAGTTTTGTTTGTGCCA-------------------------------------
DolpV_1a    GTGATAAAGTCTATGTTGATGGAGTTTGTGTAGATAACAAACCATTGTTGAGATCCAAC
            .*.* .****.*    ***     *

PdCoV_1a    --------------------------------------ACTAAAAACAAGGCAGTTCTTG
DolpV_1a    CAGGATCAACATTTTTGCGCTTGTATCCAATTTCTGATGCTGATAAACAGCATATTAAAA
                                                  .**.* **   **    .**    .

PdCoV_1a    ACTATTATGATCTTGATGCTCAAAAGTATGCTTTGTATTTACGAACACTTGCACAAAAGT
DolpV_1a    ATTATTATGAGCTAAGTTCAAAACAATATGGTGTGTTTGCTGCAGTTTATGCAAGAGAGC
            *.******** ** ..* *  ** *.**** * *** * .    *.. .  **** .*.**.

PdCoV_1a    GGGAAATTTTTCAAAAAGACAACATTACTCAACTCGTTTGGCGAGATGGTAATTGTTGGG
DolpV_1a    ACCCCATTATTGAAAGTGGTGGTTTGAGATACTTAAAACAGAAAAATAATAACTGTTTTG
            .    *** ** ***. *.....  * *  .* .* .   ..* .*.**..***.****  *

PdCoV_1a    TTAATTCAGTAGCTGTACTCCTGCAGGAAGCAAAGATAAAATTTAAGGGTTATCTTGCTA
DolpV_1a    TGTCTACTGCCTTAGTTATGTTACAGCACTTGAAGCTTGAATTCAAGGGGATAGTTAAAG
            *   * * *.  . **  * .*.*** *  ..*** * .****.*****      **.   .

PdCoV_1a    ATGCATGGGCACAGTTCTTAGGTGGAGACCCTACAGAGTTTGTTGCATGGTGTTATGC--
DolpV_1a    AATTATGGAGCAACTTTTTGGCTGGAGATGCTCGTGGAATTGTAGCTTATACCTATGCAT
            *  .****.    * **.**.* ******.  **    *..  **** ** *.    .*****

PdCoV_1a    -----------------TAGTACAAACACACAAGTAG-----------------------
DolpV_1a    TACTCTATCCTACTGTAAAGTATGGAGATATGGGTGATGCAGAAGAGGTAGTACTTAAGT
                              ****...* *.*...**..

PdCoV_1a    ------------------------------------------------GTGAATTTTGTG
DolpV_1a    ACCTTAATAAGGCAAAATTATGTGCACAATTGACATTAAATATTAAGTGCAAATGTGGTG
                                                            *..*** * ***

PdCoV_1a    ATGCCAATCTCTGTTT------GTTGAATTTGGCTCACCATTTTGATGTAGATTACACTA
DolpV_1a    AAAGAAATCACACTTTTAGTGGTGTAGATGCTGTTTACTGCATTGCTGATGTTTCTACTA
            * .  **** *  ***         *..** .  *.*.**...  *** **   * ** .****

PdCoV_1a    AAGCT---TTTATAGCTAGACGTGTAACTTGTAAGTGTG---------GAGTTAATACTC
DolpV_1a    GACATAAAGCGTTAACGAGCCGCATAGTGTGTGCTTGTGATAATGTTAAATCTAGTATTG
            .*  *     .   **.* ** **..**.. ***.  ****          .* .**.**.*
```

```
PdCoV_1a    AGCACGTGTATGGCATTACTGCCTGTTTGCAGCATGTTAGTGCTACTAATATG-------
DolpV_1a    TGTATGCAGATCTTCCTTTTGTTTTTGTTCATCGTAATGTTACGACTAAGATAAAGTTAG
             *.*.*.. **  . .* .**..* * * ** *.*.  *.  *.* ***** **.

PdCoV_1a    -----------------TTGAATTTTATGACTCAATATGGAGATTGTGATAAGTGTGGTG
DolpV_1a    AGGAAGACTTTGTTTGTGCAAATATTTTTAAGCATGGTTCACATTATTGTGGTACTAATG
                              ..*** ** * *  **  .*  * ***.* .*..   *..**

PdCoV_1a    AGAAGTATGTTGATGAAGTCACCAGCATTAACTTACCATACCTG----------------
DolpV_1a    CAATGTATAGTTATGATTCTATGAGTTCTAAAGCAGGAAAGCCGATTTTAGAATCCAGTT
             .* ****. * ****  ..*. **. .***  .*  * * *.*

PdCoV_1a    --TTTATGTTTGTATCAGGAGGAC---------------CTAAAGTTGTTGAACGTGATA
DolpV_1a    TTTCTGCCATTGTATTTGAAGGATTTTTTAAGGCCAATACCGAAGTCTCAGAGGCGATAG
              *.*..  ******. *.****.               *..****. . **.   .  .

PdCoV_1a    ACAATGTCGTTGCTAA---TATAGTCTTTTTAGGCTCTATAGAAAGCGGACATTGTTATA
DolpV_1a    ATGAGGCCATTGAGAACGGTGTGGATATTCACGGAAGTGAGGAAAACCAGGATGCTGATG
            *..* *.*.***  **   *.*.* . **.  **   *. .****.* .. **  * **.

PdCoV_1a    CACAAACACAACA------GGGTGTCTTTGATAATCTTGTTAATAACAGAAAGTTTTCAA
DolpV_1a    CTGGAAAGCAACATGCGGGAAGTACCTTCACTCCCCCT--------CGAAAAGGCGACAC
            *  .** .*****      ..**..***.. *  .*.*       *..**** .  **

PdCoV_1a    CAAAAGCATCATATATGACTGCTTACTACACACAAGTTTCCTGTGAAAAGGAGGTTGTTG
DolpV_1a    CCGAGGTGCCATTGGGCGTACTGTGGTGGAC----GTCCCCAGTAAAGGTGATGTAGGGA
            * .*.*...***  .  ..  . *. *. **    **..** **.**.. ** ** *  .

PdCoV_1a    TAGAGGCTAGTAGTTCAGGTGTGGAAGTTGAAGACATTTCTGATTTTGAGCGCTGGTATT
DolpV_1a    GTGCGGTGAAAACTGTAGAGAGTGTGAGTAATGTTATCAGTGTTCGTCAGGACCCACATT
              * **. *. * * .**. . * .. *.* * .**.  ** *. * ** .*. ..***

PdCoV_1a    C---GGAAAATTACTATGAGTGTCTTACTGTGGAGGATGTTCCACAAGTTGAAGATGCCA
DolpV_1a    CCCATGTGAATAATTTTGAAGTCCTTAGTGTGCAAAGTTTGCC---CGCTGAGAGTGCTG
            *    * .*** *.* ***.  .**** **** *...* * **    *.***...***..

PdCoV_1a    TAGATTTTACGGATTCAGCTGATTCAATTTTACCATTGACTTTGCCTGTTAGAGGTATAG
DolpV_1a    ATCCTGTTGTAAAGAAAGCTAGTGTAGTTACTATGACGGGTGATTTTAAACCCTTTAAAG
                * **...*   ****..* .*.** .  .. .*. *   ..*.       ** **
```

```
PdCoV_1a    TTAATGAAATTAATTTTAAGTCATTGAATGGTTTTACATACATCTTGAAACCAGTATCTG
DolpV_1a    TAGGAAACACTACTGTTAAACCTGGTAAAGGGCCTCAA-------TTAGAGGAGTATTTA
            * .. .* *.** * ****..*    ** ** ..*  *       * *.*  *****.*.

PdCoV_1a    GAGACAGATGTGATGTTCCTGTATATTATCCTACCTTAGATTCCATTAGTCTTAGAGCTA
DolpV_1a    ACGTTAGATGTGA--------------------------------------TTAAGCAAG
            . * .********                                      ***..   .

PdCoV_1a    TTTGGATAAATGGTGATAATAATTATGTTGTTGG--------CCATCCTAATAGTTATAG
DolpV_1a    CTTGGATAACTGGCGATTACAATGTTGTAATTCGCCGGACATCCACCCTAACAAAGATGT
            .******** ***.*** *.***  *** .** *        ***.*****.*.  **.

PdCoV_1a    TAGGTGTCTTCGTATACCTACTCTCTATGAGGAGGCAGAACAAT--------------TT
DolpV_1a    TGGGAGTTGTAGCA----------ATGGATACAGGCAAAATAGTGTTTGATAGTATGCTT
            *.** **. * *.*           *. . . *****.**.*.*              **

PdCoV_1a    ATTTCAGTAAGTGACAGCGTTGGTAATG----------TGACTTATGGCAAATGGTCTGC
DolpV_1a    AAGGCAGCAAATGCTATTATTGATTATGTAAAGAGACATAACCTTCAGAAGAAGGCAATA
            *   ***.**.** .* ..***.* ***          *.**.* ..* *.* **.

PdCoV_1a    CAAACAGCTAAACAAC-----CCATGCTGTCTTCTGTTATAACTGTTATTAAGAACATT
DolpV_1a    CAAGAAGCAAAGGAAGTTGCTACTATGTTGTCTCCTTATATGGATGTTA-----------
            ***. *** **. **..     *.***.*****.**  ***.. *****

PdCoV_1a    ATTGGTGTCGCAACACCCATTTCAACAGTTGTTAAGGAAACAGTCACTAATACAGTGCGA
DolpV_1a    ----AGGTAGTAAAAGGCATGTTGTTTAATGTCTGG-----AGCTACTTGTTGACTGTAT
                . ** *.** *  *** *.. . . ***. .*     **..*** .*  * **..

PdCoV_1a    CGTAGTCTTAACTATAATGTTGTGGGTAATGTGGTAGGTTCTATTTTAGGTTTTGTAGGT
DolpV_1a    TGCCATTGTTGCAGAAACCTACAGAATTGTATGGTGAATGTC-------------TAAAG
            .*. .*. * .* . **. *   *..* .*.****...* ..             **..

PdCoV_1a    GACTCTTTTAAACGCATTCGCGGTAAAATCTCTTTTCATCTATTAAAAACCATATTACTC
DolpV_1a    AGGTCTTCTAAGGTTGTTGGAGGTGTTAAGGCTT--------------------CTACTT
            .. ****.***.  ..** * ***.  *   ***                    .****.

PdCoV_1a    TTCTTGTTTTACTTTTGTAAAGCAAGTTTTTCAAGATTTACATGTAATTATCGTGGTGTT
DolpV_1a    GGGCTGTCATGATTTTGGCATGCATGTGCTGGTGGAGACAGAGACAAATAGC--------
               .***. *. *****  * *** ** .*   .**  .* * ..** ** *
```

```
PdCoV_1a    GTAACCAAAGTTGTTATGGCATCATTTTTCTTAACTTGGTTCTTTTACACCTATAGTGCA
DolpV_1a    ------------------------------------------------------------


PdCoV_1a    ATGCATTTTGTAGGTGTAAAAGTATTAAATTTCTTATTTG---AAGGTTCCTTATGTAAT
DolpV_1a    --------CGCAGGTGCAGTAAGATTAGCTAACTGGTTTACACTAGGCTATCTCTGTTAT
                    .*.*****.*. *. ****. *  ** .***.    ***.* ..* *** **

PdCoV_1a    AGTTATAAGGATTATGGTAAAGAAACCTTCAATATGTTGCATTATTGTGGCGACGATGCT
DolpV_1a    GATTATAATACGTACAGTGATTATCATTTTAATGTTTTTGATTATTGT---AATGGTACT
            ..****** .  **..**.*  *   .**.***.* **  ********   .*.*.*.**

PdCoV_1a    GTGTGTAGAGTCTGCCTACATGATGTAGATTCACTGCATTTGTATAAACATGCTTATAGT
DolpV_1a    CTCTGTGAATTGTGTCTGTCTGGTGTAGATTCATTACACCTGTATAAGCATGCTAAGACT
             * ***..* * **.**.. **.**********.*.**..*******.****** * * *

PdCoV_1a    ATAGAGCAGGTTTATAAGAATGCTGTAGTACAATTTAATTTTACATGGAATTGGTTTTAC
DolpV_1a    ACA---------TATCAAAAGGTTGTGAC---AACTGATTTTGTAGGCTATGCACCC---
            *.*         *** *.** *.***...   * .*.*****..* *  **  ....

PdCoV_1a    TTTTTGTTTTTATTGTTTTTCGTAAAACCAGTTGCACTTTTTATTA--------------
DolpV_1a    ------------TTATATTTTATATTGCAAGCTGTCTTTTTTAAGAGCGCAGCTATGTTG
                        **.* ***..**  .* **.**. .******  *

PdCoV_1a    --TAGTCTGTTATTGTCTGAAGTATTTAGTTTTAAATGCTGATTTTTTACAGACTGGTAT
DolpV_1a    AGTGGTTTGTTGTT-TATGATTAAGTTCACCATAAATTTTGGTGTTTTTAATACCGGTAT
              *.**.****.** * ***   * ** ... ***** .**.* ****  * **.*****

PdCoV_1a    AAGTGTCTTTGACTGGTGTCTTGTTACTTTCTTTAGTAACTTTAATTTTA----------
DolpV_1a    TTATATTGTGGATTATGTGGTATCCACAATTGCTAATACCATTGACCCTATTATGATCTC
              .*.*. * **.*.     *  ..**  *.  .**.** * **.*...**

PdCoV_1a    ------TGGGTGCAGGATTTTATGTTTGGATCTTATGGCAAATATATAAATATGCACACC
DolpV_1a    AACGATTGTGTGCGGTATGATTAAATTGTATCGTATTGCAAAT-------------CATT
                  ** ****.* **  *  . *** *** *** ******             **..

PdCoV_1a    ATATTATTTATTGTAAGGATGTAACCTGTGACATATGTAGGCGTGTTGCGCGTAACAGTC
DolpV_1a    ATTTTTATGGATGCAAGAACCCTGCTTGTGATAAGTGTCATGCAGCTACTGTATCAGAGC
            ** **  * . **.***.*. . .*.*****.* .*** .    *.*.*       .. *
```

```
PdCoV_1a    GTCATGAAGTTAGTGTTGTAGTAAATGGACGTAAGCAGTCAGTTTATGTGTATACCAATT
DolpV_1a    GAACGGAGCTCAGTTGTGTAGTTGGAAGTAAAAGGTTTCCCTATTATGTGTATGCTAATA
            *    **. *.***  ****** .. .*  . *.*.  .*   **********.*.***

PdCoV_1a    CAGGTTTTACATTTTGTAATAAACACAATTGGTATTGTAAAGATTGTGATAAGTTTGGTC
DolpV_1a    GTGTGGCTAAGATGTGCGGTAAGCATATGTGGTGCTGCAAGAATTGTGATGTATTTGGTG
              *   .** .  * **...***.**.*  ****..**.**..*********.  .******

PdCoV_1a    ACCAGAACACATTTATTTCACCTGAAGTAGCGGGTGAACTTTCTGACAAACTTAAGCGCC
DolpV_1a    AAGGTAATACTTTTATTTCCACAGCTATTGTGGGTGACCTCACCGATAAGATTAGAAGAA
            *  . **.** ********  * *  .* *.****** **.  *.**.**.  ***..  *

PdCoV_1a    ATATAAAACCTACTTCACACGCTTATTATGTTGTGGATACTGCACAATTAGTTGATGAGG
DolpV_1a    AAGTCAAGGCTACGTCAGAAGCAT---------TGGCTATTGCTACATTGATAGAAGAGG
            * .* **.  **** *** * ** *         *** **.***   ***..* ** ****

PdCoV_1a    GTTTTG---------TAAACTTTAACTACCGCAGTTCTGAACCTGGTAGTG-GTGTGAAA
DolpV_1a    GAGATAATTATGAACTCCATTATAAATTAAATGGTGCTGAAGGTAGCCGTGTGTATGATA
            *   *.          *  *.* *** *   ...** *****  *.*.  *** **.*** *

PdCoV_1a    AAACCTCAGGTTAAGTGCTTTACGGTTGCAGAATTTTTGAAAAATGCTGTTTTTCTTAAA
DolpV_1a    AAAACC-------------------------AATTTTGGAATTGTGCTATACCAGTGTCT
            *** *.                         ****** ***  .****.* ..   *

PdCoV_1a    GATGCTGTAAAGTGTGATTTTATAGAAAACAATGGTTTTATTGTTTGTAATACACAGAGT
DolpV_1a    GACGCGTCAAAGTGTGGTAAGTTTGAACCGGACAATTTTATTACCTATAATGCCAGTTCA
            **.**  .********.*     * ***   .*...*******...*.****.*  .

PdCoV_1a    TTGCATGATTTAGAGGCCGCTAAGAATGTTGCTGTCTATTATGCACACTATTTGTGTAAA
DolpV_1a    CAAAGTGAGTTGTCATTAGCAAAGCAATGTGCTGTGTATTACGCTCAAGTGTTGTCTAGA
            .  . .*** **.   .  .  ** *** *   ****** *****.** **    **** **.*

PdCoV_1a    CCTATTCTTAT------TCTTGACCAAATTCTATATAATACTCTGGCTACACAACCAGTT
DolpV_1a    CCAACCATTATTGTGCCTCTTTCTCAGGAGGAATATACTA----------GTGGTAAGGT
            ** *..  ****      ****  .**..    ***** **          .....  ** *

PdCoV_1a    TCAAAGAATTTGGTGGACAAGATTTCTGGTGTTTTG----------------GCTAGTAT
DolpV_1a    TACTAGAACACAAGTGACGCGTCTTTTAGATCTTTGTGATGATTATTACGGCATCAGTGA
            *   ****. ...  ***.  * .**.*.*  .****                ...***.
```

```
PdCoV_1a    AGTTAGTGTAAATATTGAAAACATAAACTATAATGCTGGGTCTTTGCGTGATGCTTTGTT
DolpV_1a    TGCTGAGGTAAAAATGTCTGTTATTAAGAGTAATACTGGAGCAACA--------------
             *.*.. ***** **    . .** **  .****.****. *  ..

PdCoV_1a    GTCTATTACTGACGATGAAGAAGC------------------------------------
DolpV_1a    --CTATCAGCAGCAGTGCAGCAATATGTTGCAAAGATAGTGCCTAGTCAAGTTATGAATC
              ****.* ...*..** ** *..

PdCoV_1a    ----TGTTGATTTGGCTATCTTTTGTCATAATTATGATATCGCTTATACGCAAGATGGTT
DolpV_1a    TTGATGTAGACTTTTTATTGTTTTGTGATCGTTATGGTCTAACCCCAAGTCAGGCTGCAT
                *** **.**  .  * ****** ** .*****.* * .*..  *  **.* **  *

PdCoV_1a    TTACTAACATTGTGCCTTCATATGGTATTGATGTTAGTAAAT------------------
DolpV_1a    ATAATAATGCAGTGCCCAATAGCACCTGTAATTTGGATGCATATGAGGGCGAGCAGTTTT
             ** ***... *****.    ... .  *.** * ..*. **

PdCoV_1a    ------TAACACCGCGTGATAAAGGATTTTTAGTTAACGCTGACGCTTCTATTGCTAATC
DolpV_1a    TGCGTGTCAAAGAGCAAGTTAAGAGTGCCTTTGTACAATTTGGTGTTAAGTATGTTGATT
                  * * *  **. * ***..*  ..** **  *  .**..*.*     **.*.**.

PdCoV_1a    TTAAAG------TTAGAAATGCGCCAGCAGTTGTTTGGAATTACCATGACTTGTGTAAAT
DolpV_1a    TTAAAATGGCTTTGAATGATAGCAAGATTGCTATTTTGCAATATGATTTTCTTAAGAGCT
            *****.      * *. .**.    ... *.*.*** * * **. **  ..*  . *. *

PdCoV_1a    TGTCTGGTACTTGTCTTAAATACTTAATCTCTGCTACTGTAAAGGCAGGTGGTAAGTTTT
DolpV_1a    TGCCAGGTAATTATCTGCCAAGTCTTCTTATGAGGATCAGACAAGCTGGTGTTACTCTT-
            **.* **** **.***   * ...*  *. . .  *... * *.** **** **  .**

PdCoV_1a    ATGTAACGAGATCTCTTGTTAAGCAAGTTAT---------------------TACTTGTC
DolpV_1a    --------AGAGTTACTACTAGTCAAATTATGCAGCAAGATACACTAGGGGGTGTGCGTG
                    *** .* .*..**. ***.****                     *.. .**

PdCoV_1a    ATACAGAGAAACTTATTTTGGAAAAGAAAGCTGGTGGAGTTATTAAAAACACTGCTTCTT
DolpV_1a    TTACAATGGTAGAAGATAGAGTTAGCAAGACCCAGAAGATGCCTAAGAAGAAGGGTGGTA
             ****. *. *   . *  .*  *. **..*. . ....*  .***.** *  * *  *

PdCoV_1a    ATTTTTATTTT-----------GTTTTTAAGTTCTTTTTAGTACTTTATTTGATTGTTTC
DolpV_1a    ATCCACCTTCTTGGACTCGGAGGTTCTTCAACTTTATGCAAGAGTCCTTTAAGATGAGTC
            **.. . **.*           ***.** *..*.* * .*. * *.. ** .. **  **
```

```
PdCoV_1a    TGCAGGTTGTGTTTATTATCATGCTAA---------TAGTGGTGGTTTTATACACACTAT
DolpV_1a    TTATAGGTGTATTGCTTGGTGCCTTAATGATTAGCCTAATTGCCCCCACATTTACACCGA
            *   .* ***.**  **. ... .***         **.* *.  .. .** .****..

PdCoV_1a    GTATGATGTTAACAACACTTTTCCTGTTGACAATTATAAAGTTATAGAGAATGGTGTTAT
DolpV_1a    GTGTAGTGGAATTGGCA--------GCTAGTGGCTATAAGGTTATTGATAATGGAGTTAT
            **.*..**  * ...**        *.*.......*****.***** ** ***** *****

PdCoV_1a    TAGAGATATTGTTTCTGAGGACAATTGTTTCTCTAATAAATTTTCTACTTTTGATAA---
DolpV_1a    TAGGCCTATTGTAGAGACTGATAACTGCTTTGCTAATAAATATGCTGGCTTTAATGATTG
            ***.  ******    .  **.**.**.**. ********* * **. .***.**.*

PdCoV_1a    ---------CTTTTGGGGAAGACCTTATGTTAATAGTAGGGATTGTCCAATAGTTA----
DolpV_1a    GTATTATGGTTTTGTGGGTAAGTATCCCTACAATTCTGTAGACTGTCCAGTTGTTGTTGC
                     .***  *** *... *. .  .***  *. .**.******.* ***.

PdCoV_1a    --CAGCAATTATAGAAGGTGCAGGCATTGTAGCAGCAGGAGTGCCAGGTCATGTCCAATG
DolpV_1a    GTTAACCACAATTATGGATGCCAATATGAAGGGTGCTATAGTTCCTGGTTATGC------
              .*.* *. ** . .*.*** ...** . .*  ** . *** ** ***.***.

PdCoV_1a    GGTTTTGGATCGAACTATGTTCATTCATACTACCCAGATTGAGATAAAACCATGGTATGC
DolpV_1a    ---------TGGTAATATGGCTTGGCTTAATGGTCAAATTG---TTCACTATAGGCATGA
                     * * * **** ..   * ** *. .**.****   *  * .   **.***

PdCoV_1a    ACCTAGTTGGTTTCCACATGATAATG---------TGGTAGGTTATACAACTGATTCTAT
DolpV_1a    ATTTTCATGGTTTACAGGCAGCAGTGCTGAGGGCATTGTTGGCTATACACGTGATATCGT
            *..*   ****** ** .....*.**         * ** **.******  **** ...*

PdCoV_1a    AATAACTCAAGGTGAGTTTTACCAATCAATAGCCACTATGCCTGCTAGGTGTATGTATTT
DolpV_1a    TGTCTATGGAGAAGACTTTATTAAGTCTCTTGCTTTGATTAGCGCTAGG----TGTGTTA
             .*   * .**. ** ***  . *.**  * **. . **   .******    ***.**

PdCoV_1a    AGCTAGTAGTGGTATTCAACAG-CTTTATTGTTATGGAGGTGAAAATGATGCCCCTGGTG
DolpV_1a    AATTGATGATGGTGTCTGAAAGACTTTATTGTTACGGTGGTAGTAATGATGCCATAGATG
            *..*..*..****.*...* ** ***********.** ***.. ********* . *.**

PdCoV_1a    CTATTTCTTATGAGAGCATTCAGCCTCACCGCGTTTACCTCCAACCAAATGGAATACG--
DolpV_1a    CATTGCCCTTTACTGCTATTCAACCTCATGTTGTTTATAATGATGCTATAGGCGTTGGTA
            *  * .*.* *.  . .*****.*****.  .*****.  . *  * *  ** .*  *
```

```
PdCoV_1a   -GTTTTCTATACCGCAACAGTTATTATATACACCTTATATAGTTAAATTGTCTTCTGATA
DolpV_1a   AGCTTGTAGTACCTGAACAACTTTTATATAAGCCTTATATTGTGTATACACAAGCTCGTG
            *.** . .****  ****..* ******* .******** **  *  ...   ** .*.

PdCoV_1a   ATTACTGTAAAGGTAGTGTTTGTGAAAAGACTAAGTTAGGCTATTGTTTTTCATGGAACC
DolpV_1a   AATATTGTCGCGCGGGTGTTTGTGAGCAAGCTAAGGAAGGTTATTGTATTAATTTTAATG
           * **.*** . *  .**********. *..*****  ***.****** **   *  **.

PdCoV_1a   CTCGTTGGGTGTTGTATAATGATGATTACACTGGCCTATCTGGTGTTTATTGTGGTTCTA
DolpV_1a   GTGAGTGGGCATTGTTTAACCAGCATTACACATCTAAAGATGGTGTGTATTGTGGGGAAA
            * .  ****..**** ***. *  *******   .  *  ****** ********    *

PdCoV_1a   CTATTAGGGAACTTTTATTTGTTATGGTTAATTCAT---TTTTTACAGGAGTTAACCCTA
DolpV_1a   CACCAATGAGTGTTGTTATGAGCATTATTAATGCTTATATTTATCAGGGAACCACCACTA
           *  . * *..  ** *  * . .** .***** * *   *** *  .***...* * ***

PdCoV_1a   ATATGTATTTACACCTAACTACAATGTTTTTTGTATTAGCTGCTGTGGTGTTTTGTTTTA
DolpV_1a   GTTTCTTTAATCGCTTTTGTGTTTTGAGTGTGATGATGCTCATAGCGCTAGTAGCGCTTG
           .* * * *   *.*.*   *..  **  * * .*. *. .... *.* *. *    .**.

PdCoV_1a   TGATTATGATACGCTTTCAAGGTGTGTTTAAAGCGTATGCATCAATAGTCTTTACAATCA
DolpV_1a   TTTATGTAGTTAAGTTCCAAGCATTTTTCAAAACCTATGCAGGTCTTGCGTGTGCTATTT
           *   *.*..*  . **.****   * **.***.* ******    * *. * *.* **.

PdCoV_1a   TAACTGTATGGGTTGTGAATGTGTTTATGTTGTGTGTTTATAGTTATAATCCTTTTGTTG
DolpV_1a   TATTGAGTTGGGGATTTAATGCTATTATGCTTTTGTCTTATAGTGTGAACCCAATTGTTG
           ** . .  ****   * ****.  *****.* *   .*******   **.**  ******

PdCoV_1a   CGATTATACTAGTAGCACTTTATTGTTATTGTTCACTCATGGTAAGTAAGAATGTAGCTG
DolpV_1a   TTATGCCTTTAATAGTAGTGTATATGTATGTGGCCTTAACGGTTACAACTCCTACTGCTA
           .  **  . .**.***.* * ***   ***    * .* *.*** *  *    *.. ***.

PdCoV_1a   CTATTATGCATCTATGGGTGATCTTTACATTTGTCTTAGTAGTCCCTTGGTGGATTACAT
DolpV_1a   TTATAATGCACGTTGCTTTTCTTGTAACAATTGTGCCTATGCTACCTTATAGCCTTATGG
           .*** *****. *     *  *. * *** ****..  .*. * ****.  *  ***..

PdCoV_1a   GTTTTTATATAGTTTTTGCTATTTATATGTATACCCCTTTTGTTTTGTGGTTTTATGGTA
DolpV_1a   CTATCTATGGTGCTTATGTACTATTGATGTATACTAGTGTGGCTTGTTGGTTCGTTAAGA
            * *.***.  *.** **.  * *  ********.  * * *.**  *****.  *.. *
```

```
PdCoV_1a   CTGCAAAACAAACTCGTAAACTTTATGATGGTAGTGAGTTTGTTGGTACATATGATATGG
DolpV_1a   CCAAAGTGTCCTCAGGAAAGTTATTTGAAAAGGGAGAATTTGTTGCAGATTTTGATACTG
           *.. *. ..   *  * **..* * *** .. .* **.*******  .  * *****. *

PdCoV_1a   CAGCTCAAAGTACATTTGTTATACGTAATGTTGAGTTTGTCAAATTGTCTAATGAGATAG
DolpV_1a   CAGCACGTTCAACCTTTTTGATTAATAATAGCGTGTACGTCAAGCTTGTAAATGAGGTAG
           **** *.    ** *** * **  .****. .* ** .*****..*  . ******.***

PdCoV_1a   GTGATAAGTTGGATGTTTATTTGTCTGCTTATGCCCGTCTTAAATATTACTCTGGCACTG
DolpV_1a   GCGATAAATTCCAAAAGTATCTTTCTGGTTATGCTAGGCTGAAGTATTATTCAGGTTCTG
           *.*****.**  * .  ***.* **** ******. * ** **.*****.** **. ***

PdCoV_1a   GGGGTGAACAGGATTATTTACATGCTTGTCGCGCATGGTTGGCTTATGCCCTAGATCAAT
DolpV_1a   GTGGTGACCAGGAGTGCTTAGATGCATGCAGAGCAACATTAGCTAATGCTCTTGAGAATT
           * ***** ***** *..*** **** **. * ***  .**.*** ****.** **  * *

PdCoV_1a   ATCGTTCTAGTGGCGTAGAAGTTTTATATACACCACCAAAGTTTTCTATGGGC---GCTA
DolpV_1a   TTAAGAACACACAAGTAGAGGTGCTGTATACGCCACCTAGATTTGGTGTTAATTTGGTTA
            * .   .*   . *****.** .*.*****.***** *..***  *.* ...   *.**

PdCoV_1a   GTAGGTTACAAGCTGGTTTTAAAAAGTTTGTTTCGCCTAGTAGTGTGGTAGAAAAGTGTG
DolpV_1a   CGAGGCTACAAGCAGGTATAAAGAAGATGGTCGCTCCTTCATCTGCTGTAGAACAGTGTG
             ***.******* *** * **.*** * **. * ***     **. ****** ******

PdCoV_1a   TGGTTATGGTTCGTTATAGAGGTAATGTCCTTAATGGATTATGGCTTAATGATTCTGTAT
DolpV_1a   TAGTATCTGTTGTGCATGGTAACACACAGCTTAATGGTTTGTGGTTGAATGATTATGTGT
           *.**  . ***   .**.* ...*     ******** **.***.* ******* ***.*

PdCoV_1a   ATTGCCCAAGACATGTTATGGGTAAATATAGTGGTACTGAATGGCAAGATGTACTCAACT
DolpV_1a   TATGCCCTCGTCATATACTTGGTAAGTATACTGGTGAGCAATGGAGGGATGCACTTATTA
             *****  * ***.*  * *****.**** ****.   ***** ..****.***.* .

PdCoV_1a   TAGCTAACAATCATGAATTTGAAATTGTTAGCTCAGATGGTGCAACTTTACATGTTGTCA
DolpV_1a   ATGCTAATAATTTTGATTTCCATATTTTGTAT---AAGGGTATGGAACTTCAAGTTGTGG
             *****.***. *** **. * *** *  ..   .* ***....  .* ** ***** .

PdCoV_1a   GTAGGAAGTTACAGGGTGCAGTTCTTGTTTTACAAACAGCAACTGTAAATGTTAACACTC
DolpV_1a   GCCGTGAATTGGTAGGCGCACTTTTAAAATTGAAAGTTTCTATGGTAAATGCTAATACAC
           *. * .*.**.  .**.*** **.* .  **. **..  * *. *******.***.** *
```

```
PdCoV_1a    CTAAGTATAAATTTGTTCAAGCTAAATGTGGTGATACTTTTACCATTGCTTGTTCTTATA
DolpV_1a    CTAAGTATAAGTTTGCCAAAGCTAGAATTGGTGATAACTTTTCCATTGCTTGTGCTTATA
            **********.****.. ******.*  ******** .*** *********** ******

PdCoV_1a    ATGGTAATGTCGTTGGCTTGTATCCTGTTACCATGCGTTCTAATGGAACCGTAAAGGCAT
DolpV_1a    ATGGACATGTTTCTGGTTTGTACACTGTCACACTCCGAGAAAATGGAACACTTAAAGGTA
            ****  ****. .***.*****. ****.**  * **    ********  * **.*

PdCoV_1a    CATTTTCTTGTGGTTCCTGCGGTTCTGTTGGTTATGTAATGAGTGGTAGTGTTGTAAACT
DolpV_1a    GTTTCATGTCAGGATCGTGTGGTAGTGTTGGTTATAATGTGACTAATGAAGGTGTTGAAT
              **. . *  ** ** **.***  **********.  .*** *..*.. * *** .* *

PdCoV_1a    TTTGTTACATGCACCACTTAGAGCTTCCTAATGCTATACATACGGGTACTGATTTTGCAG
DolpV_1a    TTGTTTATATGCACCATTTGGAACTTCCAGGGTGCGTGCATGGTGGTAGTGACCTTCATG
            **  ***.********.**.**.***** ..   ..*.***.  **** ***..**   *

PdCoV_1a    GTAACTTTTATGGTGGTTTTGTTGATGAAGAAAAAGCTCAGCGTGTTGAGCCTGATCTGT
DolpV_1a    GCATATTTTATGGCGGTTATGTAGACGAAGAAGTACTTCAGAGGATACCTCCTGCACCAG
            *.*  ********.**** *** **.******. * .**** * .*    ****  *..

PdCoV_1a    TAATTACTAATAATATAGTAGCATGGCTTTATGCTG-------------CTATAATTAGT
DolpV_1a    CTAATTCACGTAATATTGTTGCGTGGTTGTATGCTGCAGTCTATAATAACTGTGATTGGT
            . * * *  .****** ** **.***.* *******             **.*.***.**

PdCoV_1a    GTAAAAGAGAACACAATAACCACTCCTTCTTGGCTTGAAAAAGTGTCTGTTAGTGTTCAA
DolpV_1a    TTGTGAAA-------------------TATGGACCTAAGCAAGT---TATGTCTGTAGAA
             *. .*.*                   * * *.*.*.*. ****   *.*   ***  **

PdCoV_1a    GATTATAATAAGTGGGCTGCTGATAGTGGTTTCACTTCTTTTGTTGACAATGTGGCTATT
DolpV_1a    GATTTTAATGAGTGGGCATCTGGATATGGTTTTACCAAATTTGAGTACCACCTTGCTTTT
            **** ****.*******  ***.  .******.**.   ****   ** *. * *** **

PdCoV_1a    AATAAATTGGCTGTTTTGACTTCAATTGATGTTGGTAAGATTTTGCGTACCATTATAGTT
DolpV_1a    GATGTGTTTTCAGCAGCAACTGGAGTTTCTGTGGAGCAGATGCTTGCAGCCATAAAGGAG
            .**. .**  * *.  ..***  *.**  *** *.  **** .*    .**** * .*

PdCoV_1a    AAGCATACCCATTGGGGCCAGGAAGCTATAATGGGTTTATATAATTTTGAAGATGAACTA
DolpV_1a    TTGGCCGATGGGTGGAATTATGCCCCTGTTTTGGGTTCATTCCATCTTGATGATGAATAT
              *  .. . . ***....* *   **.*  ******.** . **.**** ******.
```

```
PdCoV_1a   ACACCTGAATCTGTTTTTAATCAAGTAGGTGGTGTTAAGTTGCAGTCTACTTTTGTGAAA
DolpV_1a   TCTCCTGAAATGATTATGCAACAAACTAGTGGCATTGTTCTACAG---AGCAATGTAAAG
            * ****** . .** *  * ***.. .****..**.  .*.***   * .  ***.**.

PdCoV_1a   AGAACATTGTCATGGTTTTGGTCTAGGACAATATTAGCTTTATTTTTGTTTGTATTGAGT
DolpV_1a   AAGGCCACTAAAGGTTTTCTATCTAGGTTGGGTTATTGGCTTGTTATGGTAATATTGACA
           *...*  .   * * ***. .****** ...  *     .*  ** ** * .******

PdCoV_1a   GCAATGGTTCTTTTTACTGTTGTACCCTATAAATATTTTATTTACGTGGCCGTAATGTTG
DolpV_1a   GTGTATGCATCTTGTAATGTAGTTCCACTTGA------TATGTTGCCACAGCTGATTATG
           *..   *. ..** ** *** ** ** . *.*     *** *   ..    *.**  **

PdCoV_1a   TTAATTGTTGTATTTTTGGCTGCA------TTTACTATAAAGCATGTATTGGCTTTTTTG
DolpV_1a   AGTGTTGGTGTAGGTTTTGCTATAATGTTTGTTACTTTGAAGCATCAACATTTCTTTATG
              .*** ****  *** ***..*       ***** *.******  *.   ..*** **

PdCoV_1a   GATACATTTTTATTGCCAACATTGTGTACGGTCATGATTGGTGTTTGTCTCGAAGTTCCT
DolpV_1a   ACAACTGCCATAATTCCCTATGTTGTTACTAGTATTTTGGGTATGGCACTTGAGAAGCCT
           .  **  .. ** * **     *   *** . .**  * ***.*    **.**..  ***

PdCoV_1a   CTTGTGTATAATAGTTATGTGCAAGATTTTCTTCGTAGTTTGTTTA--------------
DolpV_1a   GTGTTTTATAATGGATGGTACTTTAGCTTTTTCCATGACTTTTTGAAGTATGTTGGAATT
            *  * ******.* *.    .  ...***.*.*.*...** ** *

PdCoV_1a   ----------------------------TTTCATGGTATAATCCTGAAGTTATAGACACT
DolpV_1a   GAGGCTACACATCAATATGCTTGGATGCTTTTACCATTTGCGCTCTATGTGGCATATAAT
                                       ***.*. .* *.  *.. * ** ..* *.* *

PdCoV_1a   GTTGTACCTTGGCTTTTTACACCTATAATC---------------------------TTG
DolpV_1a   GTAGCAAAGGGCGTTGGTTCAACATTGATTAATTTCAATGCTGGTGGTATTAGCTTATGG
           ** *.*    *  **  * ** *  *.**.                           * *

PdCoV_1a   TATACAACTATGAAGTGTATACAGGGTTGTTGTGCTATAAATTCTCTGTCGACTGCATTA
DolpV_1a   AATTCGCTTGTGTATGGTATGCGATGGGGTAGTACACTTCGCTTTTTGTACACAGCACTG
            ** *. .*.** *  ****.*.. *  ** **.*  *  ..*.*.***  ** ***.*.

PdCoV_1a   AT------TGGTGTTTACCAAGTATTTAAGCTAGCCTTTATGGCATATACGGCTTGGAAT
DolpV_1a   ATGTCAGATAATGTTTTTGAGACCTTTGGGTCATCTGGTGAGTTTATAT----------
           **      *..***** . *... ***..*..* *.  *. * . ****.
```

```
PdCoV_1a    ACTGGTCACGCATATAGTGCAGGTAATTGGGAGCTATTTTTTGAGCTGTTGCACACAACA
DolpV_1a    -----TAATATGCATATGGCATGTGTTGGTCTGTTTTCTTCGGACCC----CGCATCAGG
                 * *.....***  *** **. * *   *.* *.**. ** *.    *.**. * .

PdCoV_1a    GTTTTGGCTAATGTTAGTAGCAATTCTATTGTAGGATTGCTTGTTTTTAATGTTGCTAAG
DolpV_1a    ATATATTATAATGTGGATAGTAAAGCAGGTGAACTATGTAGGTTACCTAGC---------
            .* *    ****** ..***.**  * . ** *  **      * ..**..

PdCoV_1a    TGGTGTTTAAATTATTGTAACCTCACTTATCTTAATTCATATGTGCTTATGGCTATATTT
DolpV_1a    -----------------TTACTTTACTGAATTGAGCTATTACACTTTTGTATTT---TAC
                             * **.*.*** * .* *..*  **... .**.*. .*   * .

PdCoV_1a    ATTAATGTCATAGGTTGGTTGTTTACATGTTATTTTGGTGTTTATTGGTGGCTTAACAAA
DolpV_1a    AGTTGTGTATTATGCTATATGTTTTCATGCTATTTTGGACTGTTCTGGTTTACTAATAAG
            * * .***  ** *.*.  ***** ****.********  * * .****   .***.**.

PdCoV_1a    GTCTTTGGTTTTACTTTTGGTAAGTACACTTTTAAAGTTAGTGTTGAACAATATAAGTAT
DolpV_1a    GTGCTTGGCACGACTTTTGGTAAGTATCCTTATAAAGTAAGTGCTGGACAATTCAAATAT
            ** .****. . **************. *** ****** ****.**.***** .**.***

PdCoV_1a    ATGTGTGTGCATAAGATACCAGCACCTAAAACAGCTTGGGATATCTTTTCAACAAATATA
DolpV_1a    ATGATGCTTAACGGTATGAGAGGACCTAGGTCAACTGCCGATGTGCTTATTACTAACATG
            ***    *  *... **.  ** *****.. **.**   ***.* .** . ** **.**.

PdCoV_1a    CTTATACAGGGTATAGGTGGTGATAGGAAACTTCCTATAGCTACAGTACAATCTAAACTT
DolpV_1a    AGGTTAGCTGGTATTGGTGGTGAAAGAACCATTACCGTGTCTACTGTGCAATCTAAATTG
                **   ***** ******** **.*   ** *..*. **** **.*********.*

PdCoV_1a    ACTGATGTTAAGTGTACTGCCGTAGTGTTAATGCAACTTTTGACTAAGCTTAATGTTGAA
DolpV_1a    ACTGATGTGAAATGTGCTACTGTGGTTTTGATGCAGTTGCTCACAAAGCTAAATGTGGAA
            ******** **.***.**.*.**.** **.*****..* .* ** ***** ***** ***

PdCoV_1a    GCTAATTCAAAGATGCATAAGCACTTAGTAGAATTGCACAACAAAATTTTAGCTTCAGAG
DolpV_1a    GCCAATTCTCGTTTACACAAGCATTTGGTACAAACACATAATGATATTTTAGCAGAGAGT
            **.*****  .  *.**.*****.**.*** ** ..**.**..* ********   ...

PdCoV_1a    GATCTTGTTGAATGTATGGATCATTTGTTAGGTATGCTTGTTACCTTGCTTTGTATAGAT
DolpV_1a    GATCCCGCTATTTGCATTGAAAAATTAACTGGTATGCTCATGACTTTGTTGTCTATTGAT
            ****..*.*.  **.** **  * **. . ********..* **.***.* * *** ***
```

```
PdCoV_1a    TCAACTGTTGATTTGAGTGAGTATTGTGAGGATGTGCTCTCTAATGTTACAGTTTTACAA
DolpV_1a    TCAACATTGGACGTGAAGGCATTGTGTGATGAATTACTCGCCAAGGAAAGTGTCTTACAG
            *****  * **. ***. * .*  ***** **  *.*** *.** *  *  **.*****.

PdCoV_1a    TCTGTAACACAAGAGTTTTCTCACATACCATCATATGCAGAATATGAACGCGCTAAAGAA
DolpV_1a    GCTGTTACTGACGAGTTTTGTGCCTTGCCTTCTTATGTGGATTATGAGAAGGCACGTGCT
             **** **  * ******* *  * *.** ** ****..** *****. . **  . *

PdCoV_1a    TTATATGAGCGTGTCCTTTTTGAATTTAAGAGCGGCAATGTTACACAACAAGAAGTTGCT
DolpV_1a    GCTTATGAA------------GAAATTCAGAAGAATAGTACTAATCCCCAAGAAATCAAG
             . *****.            *** ** ***. ...*.*..**  *  ******.*..

PdCoV_1a    GCTTATCGTAAGGCTGCTAATATAGCTAAATCTGTTTTTGATAGAGATCTTTCAGTACAG
DolpV_1a    GCATACAAGAAAGCTATGAATATAGCTAAGTCTGTTCTTGATAGGGATATTGCAGTTCAA
            ** **. . **.***.. ***********.******.*******.*** ** **** **.

PdCoV_1a    AAGAAATTAGACGCTATGGCTGAGCGTGCTATGACTACCATGTATAAAGAAGCCCGTGTT
DolpV_1a    AAGAAACTGGATGCCATGGCTGAGAGAGCTATGAGCACAATGTATAAAGAAGCGAAGAGC
            ******.*.**.**.********* * ******* .** **************  . . .

PdCoV_1a    ACTGACAGACGTGCTAAATTAGTCTCATCATTACATGCGCTTTTGTTTTCTATGTTAAAA
DolpV_1a    AGTGAGAAGAAGACTCGTTTAGTCTCATCTCTTCATGCTCTCTTATTTTCTATGATCAAG
            * *** *.. . .** . *********** .* ***** **.**.********* * **.

PdCoV_1a    AAGATAGATTCTGATAAGTTAAAATCTCTCTTTGATCATGCTAGAGATGGTGTTGTTCCA
DolpV_1a    AGACTTGATTCAGATCAGGTTGCTGATGTTTTTGAAAAAGCAAGGAATGGTGTGGTGCCT
            *.. * ***** *** ** * .    * *.*****  * ** **..******* ** **

PdCoV_1a    CTTGCAACTGTACCAATAGTTTGTAGTAACAAACTTACGTTGGTTATACCAGATGCAAAT
DolpV_1a    TTGGCTAGTGTACCGATTACTTGTTCTAACAAATTAACAATAGTTATTCCAGATATGTCT
            .* ** * ******.** ..****  *******.* **. *.***** ******...  *

PdCoV_1a    ACGTGGAACAAGTGTGTACAAGGTAGTTATGTTACCTATTCAACTGTAGTATGGAACATT
DolpV_1a    CTTTGGGAGAAGGTTGTTGTACATGATTATGTTGTGTACGGTAACATCGTTTGGGATATC
             . ***.* ***  ***   * .*..*******.. **.   * ..* ** ***.*.**.

PdCoV_1a    GACTCTGTACTAGATGCTGATGGTGTTGAACAACAACCTATTTCTGATGGTCAAAACTTA
DolpV_1a    AATGAGGTTGTTGATATGGATGG------------------------TAATCAAATTTTG
            .*.   **  * ***.. *****                        *..***** .**.
```

```
PdCoV_1a   ACGTACCCAATCTTTGGTGATAAGGTGGCTTGGCCTCTTAAAGTTAATCTTGTGCGCAAT
DolpV_1a   CCATGCCGTGTATCTGGTG---AAGCCTCGTGGCCTTTGGAAGTTAGGCTTGTGAGGAAT
            *.*.**  .* *.*****   *.*.  * ******.* .******. ****** * ***

PdCoV_1a   GCTCATAATAAAGTAGAT---GTTTCACTACAGAATAATGAGTTGATGCCACAAGGTGTA
DolpV_1a   GGAAAGCTAGTGGGTGATCTAGCTTTGTTACAGAATAATGAGTTGTTGCCTACGGGCATA
           *   *    . .*  ***   *.**...***************** ****   .**..**

PdCoV_1a   AAAACTAAGGCTTGTGTTGCTGGTGTTGATCAAACATCTTGTAACGTAGAGTCTAAGTGT
DolpV_1a   AAGAAGAGTCCTTGTGTGGCTGGTGCGAACCAGGAAAGGTGTGCTGTGCCCGCAAACGCA
           **.*  *.  ******* *******. .*.**.. *   ***. .**.    * **

PdCoV_1a   TATTATACTAATATTAATGGTAGCACAGTTGTAGCAGCCATTACATCATCTAATCCTAAT
DolpV_1a   TATTATACGAGTTTGAAAGGACAACATTTTGTAGCTGCCATAACATCAGAGGATCCAACT
           ******** *.* * ** **  .     ******* ***** ******   .**** * *

PdCoV_1a   TTGAAAGTAGCATCATTTTTAAGTGATGCTGGTAATCAGATATTTGTTGACTTGGATCCA
DolpV_1a   CTGAAGTATGCCTCTTTTACGGGAGCTTCAGGCAACTTAGTTGTAGTTGAACTTGAACCT
           .****.   ** ** *** ...* * * * **.**.. ..*  * ***** .* ** **

PdCoV_1a   CCTTGTAAATTTGGTATGAAAG---TTGGTGGTAAAGTCGAAGTTGTGTACTTGTATTTC
DolpV_1a   CCATGTAAGTTTGGTGTAAAAACACCAGATGGTGCAGTTAAAGTTATGCATCTGTATTTT
           ** *****.******.*.***.   . *.****. ***..*****.**.*..*******.

PdCoV_1a   CTTAAAAATACAAGATCTATAATTAGAGGTATGGTTCTTGGAGCCATATCTAATGTTGTT
DolpV_1a   GTTAAAAATGTTAAGAATATTGTAAGAGGTATGGTGCTTGGTGCCCTTACTAATGTAGTT
            ********.. *..  *** .* *********** ***** *** *  ******* ***

PdCoV_1a   GTTTTGCAATCTAAGGGACATGAAACCGAAGATGTTGAAGCAGTAGGTATATTATCCCTT
DolpV_1a   GCTTTGCAACATGGTGTAAGT---ACCGAATCAGTGGAAAACTGTGGTATATTAAGCCTA
           *.*******. *.. * * .*   ******   ** ***.      *********  ***

PdCoV_1a   TGTGCTTTTTCTGTAGATCCAGCTGACACGTATTTAAAGTATGTGGCTGCAGGTAATCAA
DolpV_1a   TGTAGTTTTGCTCTTGATGCTAAGGAATGTTATCTTGAATATATTAAGGAAGGAGGTGCG
           ***. **** ** * *** * .  **    ***.* .*.***.* .  * *** ..*  .

PdCoV_1a   CCATTGGGTAATTGTGTAAAAATGTTAACTGTACATAATGGTACTGGTTTTGCTATAACT
DolpV_1a   CCCCTTTCTAATTGTGTTAAGATGCTTACCTTGCATACTGGTTCTGGTGCTGCTGTAACT
           ** .*   ********* **.***.* **. *.**** **** ***** .****.*****
```

```
PdCoV_1a   ACTAAACCCAGTCCTACTAGTGACCAAGATTCATATGGTGGTGCATCAGTATGTCTTTAT
DolpV_1a   GTAAAACCGGCGCCCACACCAGAACAAGATTCCTTTGGAGGTGCTTCTGTGTGCTTGTAT
           .. ***** .  **.**    ** ******** * *** ***** ** **.**..* ***

PdCoV_1a   TGTAGAGCACATGTTGCACACCCTGGTAGTTCAGGTACATTAGACGGACGTTGTCCATTT
DolpV_1a   TGTAGAGCACATATTTCGCATCCAGGTGTTT------------ATGGAGTTTGCCAATTT
           ************.** *.**.** ***. **            *.***  ***.* ****

PdCoV_1a   AAAGGTTCTTTTGTACAGATACCTGTTACAGAAAAAGACCCTGTAGGTTTTTGTCTTAGA
DolpV_1a   AAAGGTAAATTTGTCCAGATACCCGTAGAGGAGAAAGATCCTGTAGGATTTTGCTTACGT
           ******   ***** ********.** . .**.*****.******** *****..*  *

PdCoV_1a   AATAAGATTTGTAAAGTTTGTCAATGTTGGGTAGGGTTTGGTTGCCAGTGTGATGCACTT
DolpV_1a   AATAAAATTTGTACTGTTTGTCAAATGTGGATTGGATTTGGTTGCCAATGTGATTCTTTA
           *****.*******  *********   ***.* **.***********.****** * .*

PdCoV_1a   AG---------------------------------------------------------A
DolpV_1a   AGAAAAGACCCCTTACCCATAGACCCGAAGCCTGCGGCAATGCCAATAAGACCAAAGGAA
           **                                                         *

PdCoV_1a   CAGCCTAGTATGCAAGTTGCATATGAAGAACCTAACGTTAGTACCAA-------------
DolpV_1a   CTGCTGGGAGTAAGGGCCGCGGGGGAAAAACAGCCAGGACAGCGCTGGGTTTCACCATCCT
           * **. .* .*. ..*..**. . ***.*   . * * .**..*...

PdCoV_1a   -----------------------------------------------------TGTTGAC
DolpV_1a   GGACTTGACTTCACGCGCGTAGGGGCCAATTCTGAATCACTGGAACTGCAATCTGTTCCA
                                                                ****

PdCoV_1a   AAAAATTATTTAAACGGGTACGGGGTAGCAGTGCGGCTCGACTGA
DolpV_1a   CAGAATTATTTAAACGGGTACGGGGTCTCAGTGAAGCTCGACTGA
            *.***********************  ***** .**********
```

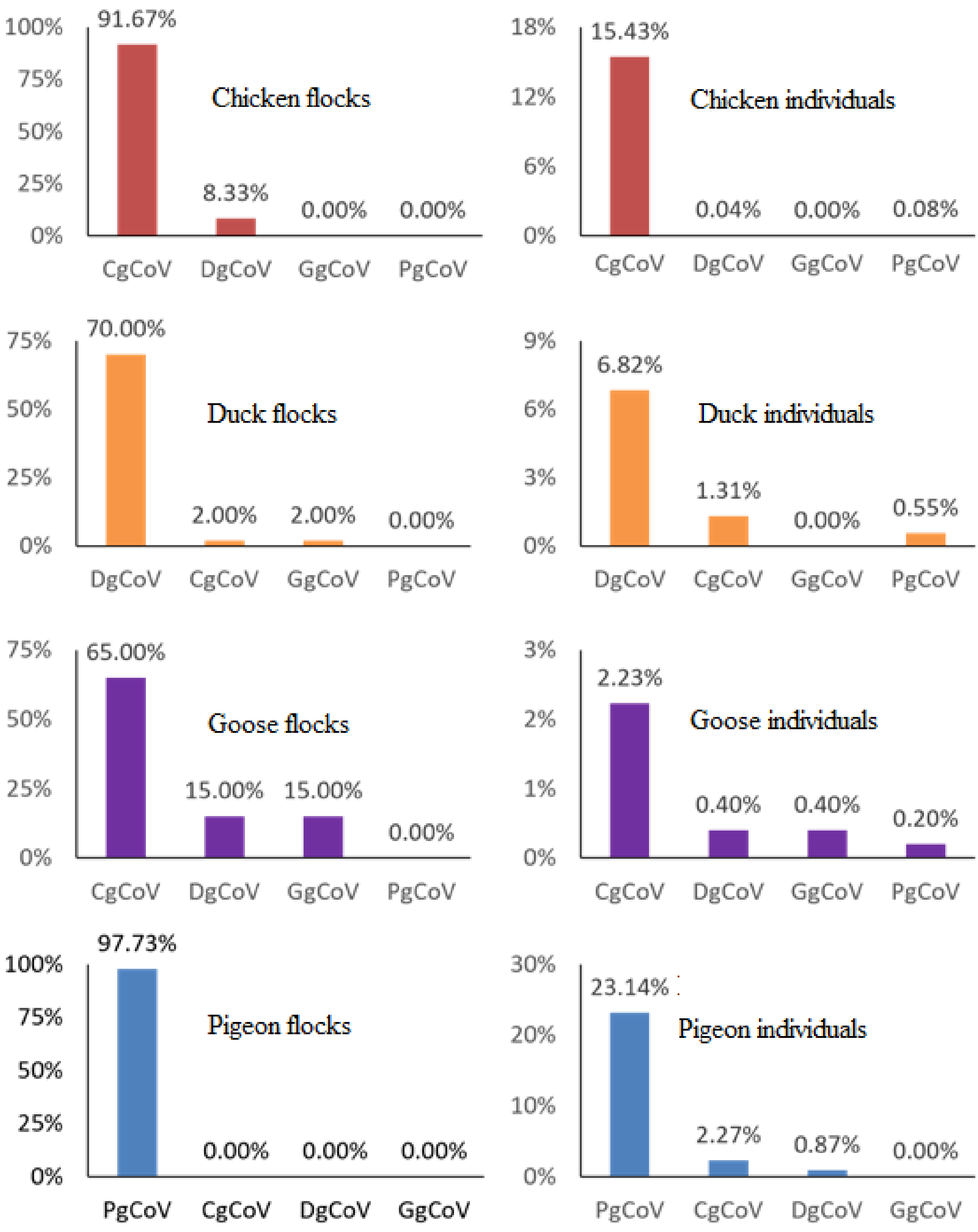

Figure S1 The positive rates in poultry flocks (from this study) and in poultry individuals (from Zhuang, et al., 2020, https://doi.org/10.1111/tbed.13541) of four gammacoronaviruses in four poultry species.
